# The Engineered Drug 3′UTRMYC1-18 Degrades the c-MYC-STAT5A/B-PD-L1 Complex In Vivo to Inhibit Metastatic Triple-Negative Breast Cancer

**DOI:** 10.3390/cancers16152663

**Published:** 2024-07-26

**Authors:** Chidiebere U. Awah, Joo Sun Mun, Aloka Paragodaarachchi, Baris Boylu, Chika Ochu, Hiroshi Matsui, Olorunseun O. Ogunwobi

**Affiliations:** 1 Department of Biological Sciences, Hunter College, City University of New York, New York, NY 10065, USA; 2 Joan and Sanford I. Weill Department of Medicine, Weill Cornell Medicine, Cornell University, New York, NY 14850, USA; 3 Department of Chemistry, Hunter College, City University of New York, New York, NY 10065, USA; 4 Ph.D. Program in Chemistry, The Graduate Center, City University of New York, New York, NY 10016, USA; 5 Ph.D. Program in Biochemistry, The Graduate Center, City University of New York, New York, NY 10016, USA; 6 Department of Biochemistry, Weill Cornell Medicine, Cornell University, New York, NY 14850, USA; 7 Hunter College for Cancer Health Disparities Research, Hunter College, City University of New York, New York, NY 10065, USA

**Keywords:** c-MYC-STAT5A/5B-PD-L1 complex, destabilized 3′UTR (AU-rich elements), mRNA poly U stabilizing elements, triple-negative breast cancer (TNBC), iron oxide nanocage (IO), nonsense-mediated decay (NMD)

## Abstract

**Simple Summary:**

The overexpression of c-MYC is implicated in many cancers, and it drives the tumors’ aggressiveness and metastatic progression, but there is no clinically approved drug that targets MYC. We discovered that the MYC mRNA is stabilized by the poly U sequences on its 3′UTR. We engineered these stable elements into unstable forms in a way such that they degraded the target MYC mRNA through a process called nonsense-mediated decay. We developed the drug 3′UTRMYC1-18 and evaluated its therapeutic efficacy in a metastatic model of c-MYC-driven TNBC in vivo by delivering it with iron oxide nanocages. The constructs inhibited primary and metastatic lung and liver cancers by degrading the c-MYC-STAT5A/5B-PD-L1 complex and achieved significant survival outcomes. The in vivo data strongly suggests that this new drug is therapeutically effective in inhibiting c-MYC-driven triple-negative breast cancer and metastatic tumors. The drug was well tolerated and represents a new arsenal to target the deadly TNBC and will offer hope to patients who need it.

**Abstract:**

c-MYC is overexpressed in 70% of human cancers, including triple-negative breast cancer (TNBC), yet there is no clinically approved drug that directly targets it. Here, we engineered the mRNA-stabilizing poly U sequences within the 3′UTR of c-MYC to specifically destabilize and promote the degradation of c-MYC transcripts. Interestingly, the engineered derivative outcompetes the endogenous overexpressed c-MYC mRNA, leading to reduced c-MYC mRNA and protein levels. The iron oxide nanocages (IO-nanocages) complexed with MYC-destabilizing constructs inhibited primary and metastatic tumors in mice bearing TNBC and significantly prolonged survival by degrading the c-MYC-STAT5A/B-PD-L1 complexes that drive c-MYC-positive TNBC. Taken together, we have described a novel therapy for c-MYC-driven TNBC and uncovered c-MYC-STAT5A/B-PD-L1 interaction as the target.

## 1. Introduction

c-MYC is a master transcription factor that binds the E-box sequences with overexpression in >74% of human cancers [1,2,3,4]. These include lymphomas and breast, lung, brain, prostate, pediatric, pancreatic, colorectal, and ovarian cancers. There are no approved direct clinical inhibitors of c-MYC, which is a significant biological and clinical challenge that needs to be overcome for more effective cancer treatment.

Of TNBCs, 61.27% are driven by c-MYC signaling [5,6]; they are estrogen receptor (ER)-, progesterone receptor (PR)-, and HER2 (ERBB2)-negative. In the USA, China, and Africa, TNBC represents 10.34%, 14.01%, and 36–79% of cases of invasive breast cancer, respectively.

Different methods have been attempted to control MYC, but with limitations. The RNAi, anti-sense oligonucleotide, G-quadruplex, and BRD4 and CDK inhibitors have high off-target effects, low cellular penetrance, unspecific POLII blockading, and indirect MYC inhibition, respectively. Only OmoMyc, a b-HLH-domain MYC mutant with enhanced leucine zipper dimerization, is in a phase 1 clinical study (https://clinicaltrials.gov/ct2/show/NCT04808362, (3 July 2023) [7,8].

To target the pervasive c-MYC expression directly and specifically in human cancers, we have developed an innovative approach based on engineering the genetic codes on the 3′UTR of the MYC gene [9,10,11,12]. We discovered that the 3′UTR of the c-MYC across many c-MYC-driven cancers is enriched with mRNA-stabilizing poly U sequences. We hypothesized that by motif engineering, we can change the stable mRNA poly U sequences to unstable forms and that we can destabilize endogenous MYC, driven by the mRNA de-capping promoter DCP1A, and overwrite the encoded mRNA with the destabilizing message and trigger its degradation via nonsense-mediated decay (NMD), controlled by UPF1 and exonucleases XRN1 and CNOT1 [13,14].

By this means, we achieved direct spatial and temporal control of c-MYC transcripts and protein and degraded the endogenous MYC message through the NMD protein UPF1 and exonucleases XRN1 and CNOT1. The three c-MYC-destabilizing constructs developed specifically destabilized and degraded the MYC transcript and proteins as well as kinases and other transcription factors, including STAT5A/B and PD-L1, which are under MYC control in TNBC cells. This killed the cancer cells via nuclei rupturing and with active caspase 3/7 expression.

In vivo, delivering IO-nanocage complexed with the destabilizing constructs into mice bearing TNBC reduced primary tumor volume by 60–80% and inhibited metastasis. Mechanistically, the destabilization of c-MYC degraded the MYC-STAT5A/B-PD-L1 complex in primary tumors and metastases that were controlled. However, in uncontrolled metastatic tumors, MYC was downregulated, but STAT5A/B was expressed, suggesting that MYC-dependent STAT5A/B-PD-L1 was controlled in the primary and metastatic tumors where our novel approach worked, whereas the nonresponsive metastatic lesions were governed by MYC-independent STAT5A/B interaction.

## 2. Materials and Methods

### 2.1. Cell Lines: Cell Cultures of MDA MB231, BT474, BT474 Clone 5, DAOY, 22Rv1, D283med, MCF7, T47D, U20S, RWPE1

MDA MB231 (RRID: CVCL_0062) was grown in DMEM supplemented with 10% FBS and 1% penicillin–streptomycin. BT474 (RRID: CVCL_0179), BT474 clone 5, MCF7 (RRID: CVCL_0031), and T47D (RRID: CVCL_0553) were grown in DMEM supplemented with 10% FBS and 1% penicillin–streptomycin. C4-2B cells (RRID: CVCL_4784) were grown in DMEM/F12 with 10% FBS, 1% penicillin–streptomycin, supplemented as described in ATCC. DAOY and D283med grew in EMEM, and 22Rv1 RWPE1 (RRID: CVCL_3791) were grown in Gibco keratinocyte serum-free medium, supplemented with bovine pituitary extract (BPE) and human recombinant epidermal growth factor (EGF) with 1% penicillin–streptomycin. Cells were grown to 80% confluency before use. U20S (RRID: CVCL_0042) grew in RPMI media. These cell lines were obtained from ATCC, and they have been authenticated by STR analysis, and the stocks are tested for mycoplasma every 3 months.

### 2.2. Mice Animal Study according to ARRIVE

We performed the animal study according to the institutional board-approved protocol. The NSG mice, 30 females (RRID: BCBC_4142) aged 6–8 weeks old, were purchased from the Jackson Laboratory. Upon receiving the animals, they were allowed to acclimate according to institutional protocol. The MDA MB231 cells were expanded as described above, and on the day of implantation, cells were harvested, washed, and resuspended in phosphate-buffered saline (PBS). The confluency of the cells was 80%. We implanted 10 million cells per ml mixed with 1 mL of Matrigel in the mammary fat pad of each mouse. After 27 days, tumors were engrafted, and on the 28th day, we randomized the mice into 6 groups with 5 mice per cage, and we ensured equal distribution of tumor size within each group. The untreated IO-nanocage-only, vector in IO-nanocage, 3′UTRMYC2-3 in IO-nanocage, 3′UTRMYC1-14 in IO-nanocage, and 3′UTRMYC1-18 in IO-nanocage treatments were administered at 20 µg per mouse intraperitoneally 12 times hourly for 3 days with a one-day dosing break, and dosing continued 12 times hourly for another 6 days. Then, dosing continued for 29 days, with 12 treatments hourly 2×/week until the end of the experiment on day 68 (Supplementary Figure S7A). Measurements of tumor size (length and width), weight, and body condition score were obtained and recorded. Upon the death of an animal, organs were harvested and stored in formalin. Pathological analyses were performed by experts from the Memorial Sloan Kettering Hospital, New York. The pathologists were blinded to the experimental details.

### 2.3. Extraction of RNA from Multiple Cancer Cell Lines

The total RNA from the MDA MB231, BT474, T47D, MCF7, and RWPE1 cells was extracted and analyzed using the RNA Easy Kit from Qiagen (Venlo, The Netherlands) (cat no: 74104). The RNA was stored at −80 °C until use.

### 2.4. Amplification of the c-MYC 3′UTR by qPCR Primers

To identify the mRNA-stabilizing poly U-rich elements on the 3′UTR of c-MYC, cDNA was made from the total RNA using the Qiagen reverse transcription kit (catalog no: 205311). Next, we performed RT-PCR using the cDNA according to the Qiagen manufacturer’s protocol using the following primer sequences; see Supplementary Table S1 (Oligonucleotides).

### 2.5. Sanger Sequencing of 3′UTR of c-MYC cDNA Amplicon

To validate the c-MYC mRNA-stabilizing poly U 3′UTR sequences, the amplified bands were excised under UV light and then extracted with a Qiagen gel extraction kit (catalog no: 28706X4) according to the manufacturer’s protocol. The amplicon was then sequenced with the c-MYC 3′UTR PCR primers by the Sanger sequencing method with equipment produced by the commercial company Psomagen Inc., Brooklyn, NY, USA. Subsequently, we used an online software DNA-to-mRNA translator (http://biomodel.uah.es/en/lab/cybertory/analysis/trans.htm, (1 January 2021)) to identify the stable poly U motifs of c-MYC 3′UTR.

### 2.6. Design of Destabilized 3′UTR of c-MYC

In the design of the destabilized 3′UTR of c-MYC, we replaced the consensus stabilized sequences with the AU-rich elements as already described [12]. Next, we modified some residues on the 3′ end of the 3′UTR to increase stability. Torabi S.F. et al. (*Science* 2020) used structural biology and biochemical assays to delineate the residues of helices of the nucleic acids on the polyA tail of 3′UTR mRNA, which determine their strong, moderate stability as well as their rapid degradation. With this information, we analyzed the loop structures of the stabilized and destabilized 3′UTR of c-MYC (Reuter JS, Matthew DH, *BMC Bioinformatics* 2010). Changes in these residues have a remarkable impact on the stability of transcripts for up to 120 min (Torabi S.F. et al., *Science* 2020). Having established the structure of stabilized c-MYC 3′UTR and the mutated residue of destabilized c-MYC 3′UTR, which increased their stability, we incorporated into these destabilized constructs an upstream 5′ BstB1 restriction site followed by a polyA sequence to stop the RFP transcription of the vector, and then followed this with the DCP1A promoter. At the 3′ end, we added a polyA sequence followed by a BamH1 restriction site.

### 2.7. Synthesis of Destabilized ARE 3′UTR of c-MYC as Gblock

The destabilized 3′UTRs of c-MYC were synthesized as gblocks from Integrated DNA Technologies (IDT Inc., Newark, NJ, USA).

### 2.8. Vector

The destabilized 3′UTRs of c-MYC were cloned into the pLenti-CMVSP6-nEGFP-SV40-PURO (Addgene: #138364).

### 2.9. Design of Gibson Assembly Primers

#### c-MYC and Vector Gibson Assembly Primers’ Design and Assembly

To generate the Gibson assembly primers, the sequences of the synthetic gblocks of destabilized 3′UTR c-MYC and the vector (Sp6 vector) were loaded into the NEB builder (https://nebuilder.neb.com/#!/) (13 June 2024), which generated the primers listed in Supplementary Table S1. The NEB HIFI DNA assembly kit used was NEB cat no: E2621S.

### 2.10. Transformation Recombination of Deficient E. coli (NEB Cat no C3019H)

For the transformation of the deficient competent *E. coli* with the DNA assembly using SOC media, 25 µL of each cell type was incubated on ice with 5 µL of the ligation mix for 30 min. After 30 min, they were heat-shocked in a water bath at 42 °C for 30 s. Tubes were placed on ice for 2 min, and 900 µL of SOC media was added, and then they were incubated at 37 °C and shaken for 1 h at 250 rpm. After this, they were plated on LB Agar plates containing ampicillin, and the plates were incubated overnight at 37 °C.

### 2.11. Colony Picking and Miniprep

We picked the colonies with pipette tips and inoculated them into 5 mL LB media containing ampicillin and grew them overnight at 37 °C while they were shaken at 250 rpm. The pellets were spurned down, and gDNA was extracted using the Qiagen Midi kit (cat no: 12943). We quantified the DNA using a nanodrop machine.

### 2.12. Colony PCR with c-MYC Gibson Primers

The PCR with c-MYC Gibson primers was performed using the PCR cycle described above.

### 2.13. Gel Extraction

The PCR products of the c-MYC 3′UTR Gibson-assembled product was gel-extracted using a Qiagen gel extraction kit (cat no: 28704).

### 2.14. Sanger Sequencing of the Cloned Destabilized 3′UTR of c-MYC Amplicon

The PCR product of the Gibson-assembled c-MYC 3′UTR produced using the Gibson primers as described above was sequenced with Sanger sequencing equipment from Psomagen Inc., Brooklyn, NY, USA. We succeeded in obtaining three positive clones: 3′UTRMYC2-3, 3′UTRMYC1-18, and 3′UTRMYC1-14.

### 2.15. Sequence Alignment of Wildtype cDNA, RNA versus the Destabilized 3′UTR c-MYC cDNA and RNA Sequences

We confirmed the engineered changes of the destabilized ARE 3′UTR of c-MYC versus the wildtype c-MYC cDNA and RNA by aligning the engineered destabilized and cloned sequences on both the DNA and RNA levels with their control wildtype using the software Clustalw Omega (https://www.ebi.ac.uk/Tools/msa/clustalo/, (1 January 2021)).

### 2.16. Transfection/Electroporation of Destabilized 3′UTR of c-MYC into MDA MB231 Cancer Cells and RWPE1 Normal Epithelial Cells

We introduced the destabilized 3′UTR into the cancer cells by electroporating the constructs into 200,000 to 500,000 cells with 50 ng of plasmid containing the constructs using a Bio-Rad electroporation system. We used the preset mammalian protocol set for 293T cells and pulsed the cells in a cuvette 2X Cells were then seeded into 6-well plates and viewed for morphology and red fluorescent protein (RFP) expression after 24 h. After 24 h, the cells expressed RFP, indicating that the constructs were successfully integrated into the cells.

### 2.17. Cell Microscopy

The cells were regularly observed under a light microscope. By day 4, the MDA MB231 wildtype containing the destabilized elements showed ruptured nuclei compared to the control.

### 2.18. Cellular Immunofluorescence

To investigate the c-MYC protein expression changes in the destabilized breast cancer cells, medulloblastoma and osteosarcoma cells were compared to wildtype cells. We seeded the cells on a tissue culture slide or 6-well plate at 2000–3000 per well. The cells were allowed to grow for 1 day, and after they were fixed with 200 µL of 4% paraformaldehyde, they were added to the cell media. The cells were placed in a 4 °C environment for 5 min, after which the paraformaldehyde was decanted. Anti-c-MYC (1:1000, cat no: 67447-1-lg protein tech) in BSA was added to the slide wells, which were then sealed with aluminum foil and kept at 4 °C overnight. After this, the antibody was removed and the secondary antibody Alexa 488 (green) or Alexa 610 (red) was added at 1:10,000, and the wells were covered with aluminum foil and kept at room temperature for 1 h. After 1 h, the secondary dye was washed with PBS (phosphate-buffered saline) 3×, and then the water was wiped off the edges with Kim wipes. Then, a drop of DAPI (Nuceloblue—nuclear stain) was added, and then the wells were sealed with cover slips, and the slides were viewed under a Nikon confocal microscope, Nikon Inc., Melville, NY, USA

### 2.19. Antibodies and Reagents

Anti-c-MYC (1:1000 67447-1-lg protein tech), Alexa 488 (green) (A30052 Thermo Fisher) or Alexa 610 (red), DAPI (Nuceloblue—nuclear stain D3571, Thermo Fisher), anti-GAPDH (ab8245), anti-caspase 7 (9492, Thermo Fisher), a human phospho-kinase array kit (cat no: ARY003C), anti-mouse IR 800CW dye (Li-COR), 4SU thiouridine, 4SU (T4509, Sigma), and anti-STAT5A/5B (ab200341) were used. 3,4-Dihydroxyhydrocinnamic acid (DHCA) by Alfa Aesar, manganese (II) acetate, oleyl amine, oleic acid, and iron(II) perchlorate were purchased from Sigma-Aldrich (St. Louis, MA, USA). p-Xylene, 1-ethyl-3-[3-(dimethyl amino)propyl] carbodiimide (EDC), and N-hydroxy succinimide (NHS) were purchased from Thermo Scientific. Tetrahydrofuran (THF), dimethyl sulfoxide (DMSO), ferric chloride hexahydrate, and phosphate-buffered saline (PBS) were purchased from Fisher Scientific. Cy7.5 dye was purchased from Lumiprobe.

### 2.20. Western Blot

We quantified the c-MYC protein expression changes in wildtype cells compared to the cells containing the destabilized 3′UTR constructs. The cells were harvested and lysed in a cocktail of a protease inhibitor in an MPER buffer. The protein extract was stored at −80 °C until use. The protein was separated into 12% stacked SDS-PAGE gels and separated by the initial run at 75 volts and after 15 min at 120 V until complete separation. The proteins were transferred to a PVDF membrane already charged with methanol at 25 V for 1 h. We blocked the membrane with BSA and subsequently incubated it with anti-c-MYC (cat no: 67447-1-lg protein tech), and the control antibody GAPDH/Actin was incubated overnight in BSA. The primary antibody was removed, and secondary anti-mouse IR 800CW dye (Li-COR) was added at 1:1000, incubated with BSA while covered from light for 2 h. After incubation, the membrane was washed with TBST 3×, and an image was taken on a Li-COR Odyssey chemiluminescent imager, Lincoln, NE, USA.

### 2.21. Cell Viability

To measure if the destabilized constructs affected cell survival, a cell viability assay comparing the wildtype cells with the cells carrying destabilized constructs was performed with a CellTiter-Glo Assay (Promega cat: G7570).

### 2.22. Phosphorylation Kinase Array

We used the human phospho-kinase array kit (cat no: ARY003C) to screen for kinases that were affected in the TNBC cells treated with the destabilized 3′UTRMYC construct compared with the vector and wildtype cells. We followed the protocol as described in the instruction manual.

### 2.23. RNA Seq

To understand the global gene expression changes in the cells treated with the destabilized 3′UTRMYC constructs, we performed RNA Seq on the MDA MB231 RNA from 3′UTRMYC1-18 compared to the wildtype and vector controls.

### 2.24. Quantitative Reverse Transcript PCR

To measure the endogenous c-MYC expression changes upon destabilization of the c-MYC, comparing the wildtype and the vector controls, we performed quantitative PCR analysis using primers that target the endogenous c-MYC exon in a time-dependent manner.

### 2.25. Generation and Structural Analysis of DNA Plasmid–IO-Nanocage Complexes

To develop the IO-nanocages, a galvanic reaction was performed in which manganese ions of templated Mn_3_O_4_ nano cubes were replaced by iron ions to form a lattice structure with a hollow center, as shown in previous publications [12,13,14,15,16]. Water-soluble IO-nanocages were engineered by capping them with 3-(3,4-dihydroxyphenyl) propionic acid (DHCA) before loading them with the DNA plasmids containing the engineered destabilized c-MYC constructs; we also created vector-only control. DNA plasmids were mixed with IO-nanocages with a mass ratio of 1:1 for the complexation based on the concentration of DNA plasmids needed for sufficient efficacy with respect to the optimized IO-nanocage concentration for in vivo experiments.

The structures of the IO-nanocage and the DNA-loaded IO-nanocages generated by the protocol above were analyzed by transmission electron microscopy (TEM), dynamic light scattering (DLS), and zeta potential. The surface charge of the neat IO-nanocage was determined to be −0.5 ±0.4 mV with zeta potential measurement. When the IO-nanocage was loaded with DNA plasmids, the surface charge was decreased significantly to −45 ± 5.0 mV, matching with the charge of the neat DNA plasmid, determined as −39 ± 6.0 mV by zeta potential (Supplementary Figure S1F). Thus, the surface charge analysis indicates that DNA plasmid is certainly complexed with IO-nanocages. Due to the existence of negatively charged carboxyl capping groups of DHCA on the exterior of the coated IO-nanocages with respect to the more neutral core, the charge gradient on the IO-nanocages created by this configuration could also be attributed to the stable complexation between IO-nanocages and DNA plasmids. Furthermore, while the TEM image (in Supplementary Figure S1D) displays IO-nanocages with a consistent cuboidal structure and a well-defined lattice structure with little to no aggregation, the TEM image of the loaded IO-nanocages (in Supplementary Figure S1E,F) displays DNA plasmids wrapped around the surface of the IO-nanocages, supporting importance of the driving force of the charge gradient for the stable complexation discussed above. According to DLS (Supplementary Figure S1G), the hydrodynamic diameter of the IO-nanocage was 28 ± 5.0 nm, with a polydispersity index (PDI) of 0.26, which is consistent with the size observed by TEM, and the low PDI can be constituted as monodisperse. After loading with DNA plasmids, the size of the IO-nanocage increased to 780 ± 150 nm. As the size of neat DNA plasmids is much larger than the size of IO-nanocages, which is 1.306 ± 0.25 μm by DLS, the size increase of IO-nanocages after the complexation with DNA plasmids is plausible. Transmission electron microscopy (TEM) showed that DNA constructs and vectors were wrapped around IO-nanocages and the zeta potential of the DNA construct–IO-nanocage complex shifted to a highly negative value as compared to the neat IO-nanocage due to the wrapping of negatively charged DNA constructs, consistent with the observation in the TEM image. Dynamic light scattering (DLS) also revealed that the hydrodynamic size of the DNA construct–IO-nanocage complex increased to 800 nm due to the wrapping of the DNA construct; this hydrodynamic diameter is smaller than the one of the neat DNA construct, indicating the compacting of the construct on the IO-nanocage (Supplementary Figure S1G).

### 2.26. DHCA-Coated IO-Nanocage Synthesis

To synthesize the IO-nanocages with oleic acid, a modification was made using the previously published method [13,14,15,16,17] Manganese (II) acetate (0.17 g), oleyl amine (0.82 mL), and oleic acid (0.16 mL) were added to p-xylene (15 mL) in a three-necked 50 mL flask with a reflux condenser and sonicated for 10 min. The flask was heated to 90 °C in air under magnetic stirring, and then 1 mL of deionized water was rapidly injected into the flask. The reaction mixture was heated at 90 °C for 1.5 h, producing Mn_3_O_4_ nanoparticles. One mL of 2.4 M aqueous iron(II) perchlorate solution was added, and the mixture was maintained at 90 °C for an additional 1.5 h to produce IO-nanocages by galvanic replacement. After cooling, the IO-nanocages were collected by centrifugation, rinsed with ethanol, and dispersed in THF.

Then, the IO-nanocages were capped with DHCA and transferred to the aqueous phase using the following modified version of a previously published method [12,13,14,15,16]. First, 100 mg of DHCA was dissolved in 5 mL of THF in a three-necked flask (25 mL). The resulting solution was heated to 50 °C after bubbling for 30 s with flowing nitrogen gas. Then, 20 mg of IO-nanocages capped by oleic acid were dispersed in 1 mL of the THF, which was added to the solution. The solution was heated to 50 °C for 3 h, and then cooled to room temperature, and 500 µL of NaOH (0.5 M) was introduced to precipitate water-soluble IO-nanocages. The precipitate was collected by centrifugation and redispersed in 2 mL water, then dialyzed overnight with a 10 K MWCO membrane.

### 2.27. Complexation of DNA Plasmids with IO-Nanocages

To conjugate the DNA plasmids with the IO-nanocages, the IO-nanocages were vortexed for an even distribution of particles. The conjugation was completed with a 1:1 mass ratio between the DNA plasmid and the IO-nanocage, and the samples were incubated overnight at 4 °C. The DNA plasmid complexations with IO-nanocages were then confirmed by TEM imaging, zeta potential, and DLS.

### 2.28. Structural Analysis of DNA Plasmid-Loaded IO-Nanocages by TEM

The DNA-loaded IO-nanocages were then further processed for imaging using a transmission electron microscope as described previously. Uranyl acetate, filtered using a 0.2 µM filter with a 3 mL syringe, was used for staining DNA plasmids for TEM. After 10 µL of the loaded IO-nanocage sample was placed on TEM grids for 10 min, 5 µL of filtered uranyl acetate was added to these grids. After 45 s, the excess uranyl acetate was removed and added again on the grid for two more cycles. Finally, these samples were imaged by a JEOL JEM-2100 transmission electron microscope at 200 kV with an LaB6 gun. Images were captured using a 4 GB Gatan UltraScan camera, model 994 US 1000XP and a Digital Micrograph V.2.1.

### 2.29. Migration Assay

Wound healing assays were performed on the cell monolayer using a sterile 200 μL pipette tip. Cells were washed with 1× PBS, and fresh media were added to each well following the wound. Images of scratched areas were taken at 10× magnification using an AE30 inverted microscope (Motic, Richmond, BC, Canada).

### 2.30. Tissue Immunofluorescence

Tissue slides were stained with anti-c-MYC and STAT5A/B. Briefly, slides were washed with PBS (1×) and primary antibody was added (c-MYC and STAT5A/B) at 1:1000 and dropped onto the tissue to incubate overnight. After this, it was washed off, and the secondary antibody was added after 1 h; then, it was washed, DAPI was dropped on, the cover slip was mounted, and the image was captured.

### 2.31. PD-L1 Immunohistochemistry

The PD-L1 staining of the tumors and tissue was performed by the expert pathologists of the Memorial Sloan Kettering Cancer Center, the core pathology laboratory, in New York.

### 2.32. Prussian Blue Staining and Detection of IO-Nanocage in Tissues

To identify the iron oxide nanocages in the treated animals and the control, Prussian blue staining on the tissue slides with and without the treatment of MYC-incorporated IO-nanocages was performed as follows. First, the tissue slides were deparaffinized with xylene for 10 min, 100% ethanol for 9 min, 95% ethanol for 6 min, 70% ethanol for 3 min, and, lastly, hydrated with MilliQ water for 5 min. Then, the tissue slides were placed in Working Iron Stain Solution (Sigma-Aldrich) for 30 min as the Working Iron Stain Solution was prepared by mixing equal volumes (100 mL) of potassium ferrocyanide solution and hydrochloric acid solution. The tissue slides were then collected and rinsed thoroughly in MilliQ water. The tissue slides were then placed in the working pararosaniline solution (Sigma-Aldrich) for 3–5 min as the working pararosaniline solution was prepared by mixing 1 mL of pararosaniline solution with 50 mL of MilliQ water. After the tissue slides were collected and rinsed thoroughly in MilliQ water, the tissue slides were then rapidly dehydrated through a series of alcohol treatments and cleaned with xylene, as stated above, before mounting. One drop of Per mount Mounting Media (Fisher Scientific, Waltham, MA, USA) was placed on the tissue slide and a cover slip was placed on top of the tissue slide for imaging.

### 2.33. 4SU mRNA Labeling Pulse Chase Experiments

In a 6-well plate, we seeded 1 million cells each of the MDA MB231 WT, vector, 3′UTRMYC2-3, 3′UTRMYC1-18, and 3′UTRMYC1-14 and treated them with 4SU at 1 ug/mL and collected cells at 0 h, 30 min, 1 h, 3 h, 6 h, and 24 h. We collected RNA, made cDNA, and performed RT-qPCR with primers targeting the endogenous c-MYC as well as primers targeting the DCP1A promoter of the vector and the cloned destabilized c-MYC; the control primer was ACTB. We calculated delta Ct (Ct target endo c-MYC or exo destabilized c-MYC-CtActB) and the fold enrichment was 0.5^Ct.

### 2.34. Targeted Sequencing to Detect Genomic Sites of Construct Integration

To determine the sites of the integration in the genome, we extracted the gDNA from tumors and tissues of the animals and then used primers that can detect both the vector and destabilized constructs. This approach allowed us to detect the vector and the constructs together. The extracted gDNA was sent to Psomagen Inc. in New York, and the amplified reads were mapped back to the human genome to detect sites where they are integrated.

### 2.35. Quantification and Statistical Analysis

#### Cell Viability Measurement

After we acquired the cell viability measurement as luminescence, to obtain the percentage survival of each cell, we normalized the treated cells with the untreated cells in multiple replicates and plotted the graphs in GraphPad Prism, and the two-tailed *t*-test was used to determine statistical significance.

### 2.36. Immunofluorescence

The immunofluorescence staining for c-MYC and STAT5A/5B was performed as described above. We obtained the images using a Nikon confocal microscope and ImageJ (ImageJ.net) software, obtained the fluorescence intensity, and subtracted the background intensity. We plotted the multiple replicates data in GraphPad Prism, and the two-tailed *t*-test was used to determine statistical significance.

### 2.37. Transcript Quantification

We obtained the mRNA expression of c-MYC, CNOT1, XRN1, EEF2, endogenous MYC, and the engineered destabilized 3′UTRMYC and GAPDH for each treatment group and control by quantitative PCR. The expression of target genes was obtained as described above (we calculated delta Ct (Ct target-CtActB or CtGAPDH) and fold enrichment as 0.5^Ct, the multiple replicates data were plotted in GraphPad Prism, and the two-tailed *t*-test was used to determine statistical significance.

### 2.38. Tumor and Animal Weight Measurement

We obtained the tumor volumes by daily measurement of the widths and lengths of the tumors with a caliper; this was recorded daily from randomization until the end of the experiment. We used the standard formula for calculating tumor volume (1/2XWXWXL). The data were presented as a daily graph. Daily weight measurements were obtained with a weighing scale in grams and plotted as a daily weight chart. A two-tailed *t*-test was used to determine statistical significance between treatment and control groups.

### 2.39. Mouse Survival Quantification

We recorded the daily survival events of the animals across the treatment and control groups. Alive was scored as 0 while death was scored as 1. These data were plotted as a survival analysis on GraphPad Prism as a Kaplan–Meier curve. Statistical significance was determined by log-rank (Mantel–Cox) testing between treatment and control groups.

### 2.40. IHC Image Capturing and Measurement

The immunohistochemical PD-L1 staining and H&E staining were performed by expert pathologists at the Memorial Sloan Kettering Cancer Center. They were blinded to the details of the experiment. We acquired the images on a Nikon confocal microscope, and ImageJ was used to obtain the staining intensity normalized to the background. These data were plotted on GraphPad Prism, and statistical significance was determined by the two-tailed *t*-test.

### 2.41. Phosphorylation Kinase Assay Quantification

We measured the intensity of the dot blot using MYImageAnalysis (Thermo Fisher Scientific, Waltham, MA, USA) and plotted the data on GraphPad Prism, and statistical significance was determined using the two-tailed *t*-test.

### 2.42. Migration Assay Measurement

The distance of the wound healing assay was measured with a meter rule at 10× magnification using an AE30 inverted microscope (Motic, Richmond, BC, Canada). The data were plotted on GraphPad Prism, and the two-tailed *t*-test was used for statistical significance.

### 2.43. Prussian Blue Stain Quantification

We quantified the absolute number of the Prussian blue stain on the microscopic slides of the stained tissue by counting on ImageJ. These data were plotted on GraphPad Prism, and the two-tailed *t*-test was used for statistical significance.

### 2.44. Number of Experimental Replicates and Statistical Analysis

All experiments were done in multiple replicates. The cell-based experiments were performed a minimum of 3×, as indicated in the legends. All animal data presented represent experiments performed on 5 mice in a cage per group. This includes the pathology work, H&E staining, PD-L1 staining, and c-MYC and STAT5A/5B staining. Spearman correlations were performed to establish correlation. A two-tailed *t*-test was used to determine statistical significance. Log-rank (Mantel–Cox) tests were used to determine statistical significance from the Kaplan–Meier survival curve between treatment groups and the control.

### 2.45. ImageJ

We used the software ImageJ to quantify the immunofluorescence signal of c-MYC and the Western blot.

### 2.46. GraphPad Prism

We used GraphPad Prism software version 10.2 to draw all the bar charts presented and performed all statistical analysis with the software.

## 3. Results

### 3.1. Engineered Destabilized 3′UTR of MYC Degrades c-MYC-STAT5A/5B-PD-L1 Complex to Inhibit Primary and Metastatic Tumors in c-MYC-Driven TNBC In Vivo with Significant Survival Outcome

Sanger sequencing identified the poly U mRNA-stabilizing elements on the 3′UTR of the c-MYC gene that were 99% conserved across breast cancer cell lines (Supplementary Figures S1A–E and S2A). To generate the destabilized c-MYC 3′UTR, we replaced all the stabilizing elements with destabilizing elements, which are CCUC, CCUGC, and ACUUAU (Supplementary Figure S3A). We then engineered the cDNAs to be driven under the control of the mRNA de-capping enzyme gene (DCP1A) promoter (Supplementary Figure S4A,B). We created three c-MYC-destabilizing constructs (3′UTRMYC2-3, 1-18, and 1-14) (Supplementary Figure S5A–C). The logic behind the design [9,10,11,12] was that if we destabilized the MYC 3′UTR and drove its expression by DCP1A, we could degrade the MYC transcript and protein specifically through nonsense-mediated decay by overwriting the endogenous messages with an infidel-exogenous destabilized transcript that was made at a high, copious amount [13,14]. As designed, these constructs triggered nonsense-mediated decay (Supplementary Figure S12A–E) at an extremely high level (*p* = 6.1 × 10^−8^, FDR = 5.3 × 10^−6^), leading to endogenous c-MYC destabilization and degradation.

**Figure 1:**
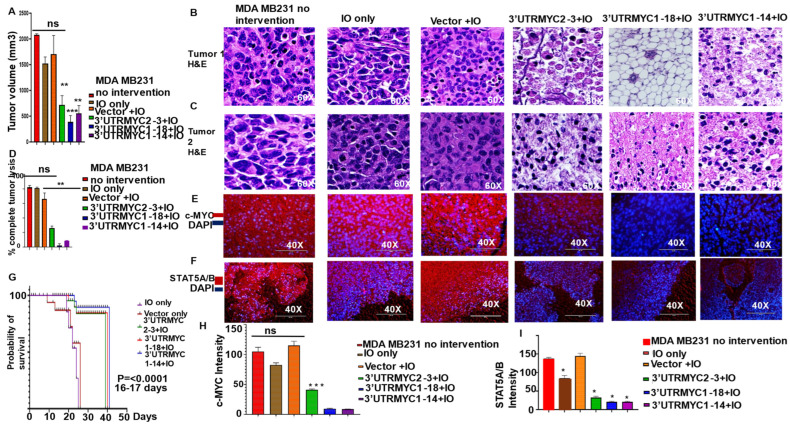
In vivo, IO-nanocage-delivered destabilized 3′UTR of c-MYC degrades c-MYC and STAT5A/5B and shows significant survival outcome and the inhibition of primary and metastatic tumors in c-MYC-driven TNBC. (**A**) Bar chart shows the tumor volume measurement of animals bearing tumors: WT, treated with nanocage only, vector + IO-nanocage, 3′UTRMYC2-3 + IO-nanocage, 3′UTRMYC1-18 + IO-nanocage, and 3′UTRMYC1-14 + IO-nanocage, (N = 5). Paired two-tailed *t*-test (* *p* = 0.0004, WT vs. 3′UTRMYC1-18 + IO, * *p* = 0.0021, 0.0015WT vs. 3′UTRMYC2-3 + IO, 3′UTRMYC1-14 + IO). (**B**) H&E staining of primary tumor images from the different control groups and the treatment groups (N = 5). (**C**) H&E staining of primary tumor images from the different control groups and the treatment groups (N = 5). (**D**) Bar chart of quantification of tumor lysis from the different control groups and treatment groups (** *p* = 0.0032, paired two-tailed *t*-test, WT vs. 3′UTRMYC1-18 + IO). (**E**) Immunofluorescence images of primary tumors from the controls and treatment groups stained with c-MYC (red), DAPI nuclei (blue) (N = 5). (**F**) Immunofluorescence images of primary tumors from the controls and the treatment groups stained with STAT5A/5B (red), DAPI nuclei (blue) (N = 5). (**G**) Kaplan–Meier survival plot of animal experiment. * *p* < 0.0001 (WT, IO-nanocage only vs. 3′UTRMYC2-3, 1-18, and 1-14) (N = 5). (**H**) Quantification of STAT5A/5B in primary tumors from the controls and the treated groups. * *p* < 0.03 (WT vs. IO-nanocage only, 3′UTRMYC2-3). * *p* < 0.01 (WT vs. 3′UTRMYC1-18 and 1-14) (N = 5). (**I**) Quantification of c-MYC in primary tumors from the controls and the treated groups. * *p* < 0.03 (WT vs. 3′UTRMYC2-3, 1-18, and 1-14) (N = 5).

To prove in vivo that our constructs indeed inhibited tumor growth, we administered 20 µg of the constructs and the controls packaged in nanocages intraperitoneally into the mice bearing TNBC tumors in their mammary fat pads (Supplementary Figure S8A–C). The survival analysis comparing the animals that received the constructs (3′UTRMYC2-3, 1-18, and 1-14) with the no-intervention animals shows that the treated groups’ survival improved very significantly (*p* < 0.0001), with 17 days more than the control groups (Figure 1G). The construct 3′UTRMYC1-18 inhibited the tumor the most (Figure 1A–C). The 3′UTRMYC1-18 + nanocage caused an 80% reduction in the tumor volume; the 3′UTRMYC2-3 + nanocage and 3′UTRMYC1-14 + nanocage achieved a 60% tumor volume reduction (Figure 1A–C, Supplementary Figures S4E and S8B) compared to the controls. 3′UTRMYC1-18 achieved near complete tumor lysis (Figure 1D) and completely eradicated the tumors histologically compared to any other group, whereas the 3′UTRMYC2-3 and 1-14 achieved partial tumor response (Figure 1D). We found no change in body weight when comparing the control and treatment groups (Supplementary Figure S8C). This finding strongly shows the therapeutic benefit of directly drugging MYC using this approach.

To validate the loss of c-MYC and its interacting partner STAT5A/5B in vivo, we stained tumor tissues, as shown in Figure 1E,F, with antibodies against c-MYC and STAT5A/5B, respectively. We found that the tumors that responded to the treatment with the constructs 3′UTRMYC2-3, 1-18, and 1-14 showed a loss of c-MYC and STAT5A/B (Figure 1E,F,H,I) compared to the controls. The loss of c-MYC was most profound in the tumors that received the constructs 3′UTRMYC1-18 and 1-14. Taken together, this demonstrated that the loss of c-MYC led to the loss of STAT5A/5B in vivo, validating our mRNA data and indicating that the constructs were effective. It was especially significant that the 3′UTRMYC1-18, 1-14, and 2-3 achieved a complete pathological response, a near-complete pathological response, and a partial response, respectively, in inhibiting the deadly c-MYC-driven TNBC.

### 3.2. Engineered Destabilized 3′UTR of c-MYC Degrades c-MYC Transcript and Proteins in c-MYC-Driven TNBC and Medulloblastoma and Prostate Cancer with Specificity, and It Is Safe for Normal Healthy Epithelial Cells

**Figure 2:**
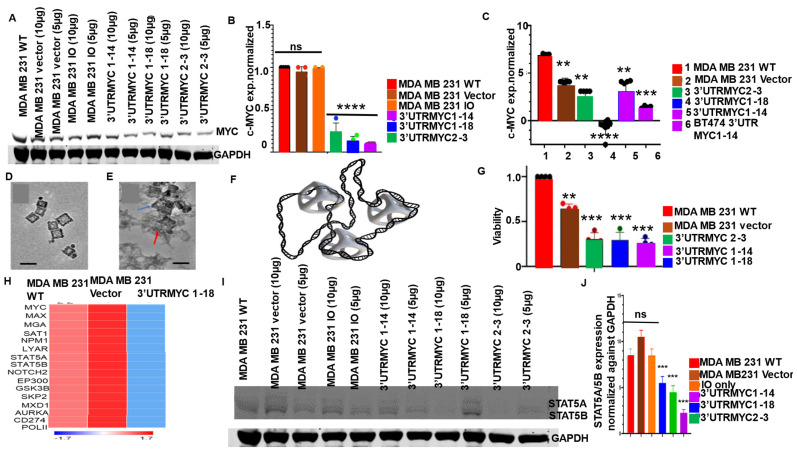
The engineered destabilized 3′UTR of c-MYC degrades MYC transcript and protein in TNBC. (**A**) Western blot shows c-MYC and GAPDH protein expression in MDA MB231 WT (wildtype or no intervention) cells, and cells treated with vector, IO-nanocage only, and 3′UTRMYC1-14, 3′UTRMYC1-18, and 3′UTRMYC2-3 via IO-nanocage delivery. N = 4. (**B**) Bar chart shows c-MYC mRNA expression normalized against GAPDH in MDA MB231 WT cells, and cells treated with vector, IO-nanocage only, and 3′UTRMYC1-14, 3′UTRMYC1-18, and 3′UTRMYC2-3 cells treated via IO-nanocage delivery. Two-tailed *t*-test, **** *p* < 0.00001 (WT, vector, IO-nanocage versus 3′UTRMYC1-14, 1-18, and 2-3). N = 5. (**C**) Bar chart shows c-MYC mRNA expression normalized against GAPDH in MDA MB231 WT, vector, 3′UTRMYC2-3, 1-18, and 1-14, and BT474 cells expressing 3′UTRMYC1-14. Two-tailed *t*-test, ** *p* < 0.001 (WT vs. vector, 3′UTRMYC2-3 and1-14), **** *p* < 0.00001 (WT vs. 3′UTRMYC1-18), *** *p* < 0.0001 (WT vs. BT474 3′UTRMYC1-14). N = 5. (**D**) High-resolution transmission electron microscopy (HR-TEM) image displays IO-nanocages with consistent cuboidal structure and well-defined lattice structure with little to no aggregation. (**E**) Transmission electron microscopy (TEM) image of the loaded IO-nanocages (blue arrow) in displays of DNA plasmids wrapped around the surface of IO-nanocages (red arrow), 20× magnification. (**F**) Diagrammatic illustration of DNA constructs wrapped around the IO-nanocages (1:3). (**G**) Bar chart shows viability of cells treated with constructs. ** *p* < 0.001 (WT vs. vector), *** *p* < 0.0001 (WT vs. 3′UTRMYC2-3, 1-18, and 1-14). N = 6. (**H**) Heat map shows gene expression pattern changes of MYC interacting partners compared to controls and the 3′UTRMYC1-18-treated cells. (**I**) Western blot shows STAT5A/5B and GAPDH protein expression in MDA MB231 WT cells and cells treated with vector, nanocage only, 3′UTRMYC1-14, 3′UTRMYC1-18, and 3′UTRMYC2-3. N = 3. (**J**) Bar chart shows the quantification of STAT5A/5B protein expression normalized against GAPDH in the treated and control groups (*** *p* < 0.001, **** *p* < 0.0001, two-tailed *t*-test). Original western blots are presented in File S1.

To validate the degradation of the c-MYC transcript and proteins in TNBC, the TNBC cells were treated with the engineered destabilized 3′UTR c-MYC constructs packaged in iron oxide nanocages (IO-nanocages) as previously described [15,16,17,18,19] (Figure 2A–F, Supplementary Figure S1F,G). After 4 days of incubation, we found that all three constructs of 3′UTR (MYC2-3, 1-18, and 1-14) degraded c-MYC protein and the transcript in MDA MB231 compared to the controls (Figure 2A,B). This degradation of c-MYC led to the significant loss of viability of these cells that received the constructs compared to the controls (Figure 2G). The loss of c-MYC increased active caspase 7 (Supplementary Figure S4C,D), leading to the impaired migration of these cancer cells (Supplementary Figure S11A,B).

**Figure 3:**
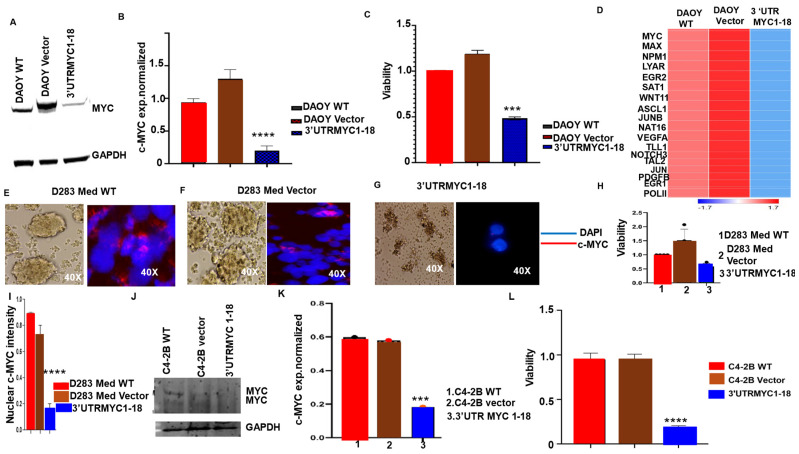
3′UTRMYC1-18 degrades c-MYC in childhood cancers and prostate cancer and impaired their viability. (**A**) Image shows c-MYC expression in DAOY treated with the 3′UTRMYC1-18 and the controls. (**B**) Bar chart shows the quantification of c-MYC protein normalized against GAPDH in the treated and control cells (**** *p* < 0.0001, two-tailed *t*-test). (**C**) Bar chart shows the quantification of the viability in the 3′UTRMYC1-18-treated cells and the control (*** *p* = 0.001, two-tailed *t*-test). (**D**) Heat map shows the expression of c-MYC and its interactome in the 3′UTRMYC1-18-treated cells and controls. (**E**–**G**) Images show c-MYC stain (red) and DAPI (blue) of the 3′UTRMYC1-18-treated cells and controls, quantified in (**H**). (**I**) Bar chart shows the quantification of the nuclear c-MYC stain in the 3′UTRMYC1-18-treated cells and controls (**** *p* < 0.000121, two-tailed *t*-test). (**J**) Western blot image of the c-MYC and GAPDH protein expression in the C4-2B cells treated with the 3′UTRMYC1-18 and the controls. (**K**) Bar chart shows the quantification of c-MYC expression normalized against the GAPDH in the treated cells and controls (*** *p* < 0.001, two-tailed *t*-test). (**L**) Bar chart shows the quantification of the viability of the 3′UTRMYC1-18-treated cells and the controls (**** *p* < 0.0.0001, two-tailed *t*-test). Original western blots are presented in File S1.

To confirm the degradation of the c-MYC transcript and its interactome, we performed RNA Seq of the 3′UTRMYC1-18-treated cells and the controls. We found that the destabilized c-MYC constructs degraded c-MYC mRNA expression (Figure 2H) as well as c-MYC interacting partners with a known high MYC binding site, MAX, MGA, SAT1, NPM1, LYAR, STAT5A/B, NOTCH2, NOTCH3, EP300, GSK3B, SKP2, MXD1, AURKA, CD274, POLII, WNT11, ASCL1, JUNB, NAT16, VEGFA, TLL1, TAL2, JUN, PDGFB, and EGR1, in the cancer cells carrying the destabilized 3′UTRMYC1-18. These findings strongly suggest that this novel technology reliably controlled the c-MYC transcript, protein, and interactome. In the same manner, we investigated the generalizability of the technology in targeting c-MYC across other cancer types such as medulloblastoma and prostate cancer (Figure 3A–L). We found that the 3′UTRMYC1-18 degraded c-MYC in two medulloblastoma lines (DAOY and D283med) and prostate cancer (C4-2B) and impaired their viability (Figure 3A–L). These data suggest the applicability of the technology to control c-MYC-driven cancers regardless of the tissue of origin.

We have previously shown that some kinases are uniquely downregulated only upon the degradation of c-MYC [12]. These kinases are p38α pT180/Y182, JNK1/2/3 pT183/pY185 [20], pT221/pY22, GSK3α/β pS21/pS9 [21,22], PDGFRβ pY751 [22], LckpY394, and STAT5A/B pY694/p699 (Supplementary Figure S6A). We chose STAT5A/5B as there is a paucity of information on c-MYC-STAT5A/5B interaction in cancers. We found that cells treated with the 3′UTRMYC1-18 and 3′UTRMYC2-3 show a loss of STAT5A and STAT5B upon the loss of c-MYC at day 4 (Figure 2I,J). A trend emerges, which is the concomitant loss/reduction in the c-MYC and the STAT5A/B upon MYC destabilization (Figure 1I,J, Supplementary Figure S6A–C).

To prove the safety of the engineered c-MYC constructs to the normal epithelial cells, as we have demonstrated for the ERBB2 [12], we treated the normal RWPE1epithelial cells with the c-MYC constructs in independent 4- and 8-day experiments. We found no loss of c-MYC on either the transcript or protein levels in the treated cells compared to the controls (Supplementary Figure S7A–F,I). In summary, these results show that the engineered destabilized 3′UTR does not affect normal healthy cells; this is supported by our findings for ERBB2 [12]. To confirm the specificity of the MYC constructs and show that it does not affect other oncogenes, we probed for the loss of c-MYC in NCI H1975 destabilized with the engineered 3′UTR of ERBB2 that targets ERBB2. We found no loss of c-MYC in the desARE3′UTR ERBB2 cells (Supplementary Figure S7G–H). Conversely, we found no loss of ERBB2, EGFR, ABL1, RELA, and RELB transcript in c-MYC-destabilized cells compared to controls (Supplementary Figure S6E,F), and the 3′UTRMYC1-18-treated cancer cells tended to restore MYC expression towards the normal female mammary epithelial expression levels (Supplementary Figure S6D). This was compared using the ENCODE data, and, thus, it offers a plausible explanation as to why RWPE1’s normal MYC expression was not affected, as it is at normal levels. We also validated that the constructs are not integrated in hot spots or oncogenic loci [23] (Supplementary Figure S19A–C).

**Figure 4:**
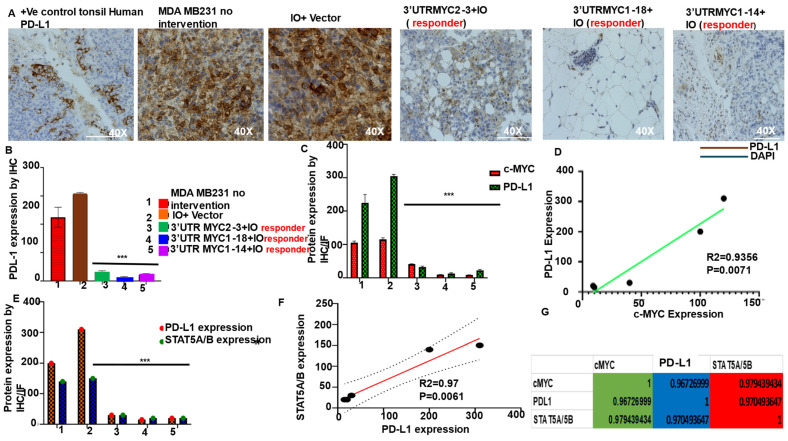
Loss of c-MYC leads to loss of STAT5A/5B and PD-L1 in vivo in responsive tumors. (**A**) Immunohistochemistry staining of PD-L1 in primary tumors from the control and treated groups; positive control is human tonsil; PD-L1 (brown); DAPI nuclei (blue) (N = 5). (**B**) Quantification of PD-L1 in primary tumors from the control and treated groups, *** *p* < 0.0005 (WT, IO-nanocage + vector vs. 3′UTRMYC2-3, 1-18, and 1-14). (**C**) Head-to-head quantification of PD-L1 and c-MYC in the same primary tumors from the control and treated groups, *** *p* < 0.005 (WT, IO-nanocage +vector vs. 3′UTRMYC2-3, 1-18, and 1-14). (**D**) Graph shows the correlation analysis between PD-L1 and c-MYC in the primary tumors, R^2^ = 0.9356, *p* = 0.0071. (**E**) Head-to-head quantification of PD-L1 and STAT5A/5B in primary tumors from the control and treated groups, *** *p* < 0.0005 (WT, nanocage +vector vs. 3′UTRMYC2-3, 1-18, and 1-14). (**F**) Graph shows the correlation analysis between PD-L1 and STAT5A/5B in the primary tumors, R^2^ = 0.97, *p* = 0.0061. (**G**) Table shows the multiple correlation analysis between c-MYC, STAT5A/5B, and PD-L1 in the primary tumors from the control and treated groups.

Tumors upregulate PD-L1 to evade immune cells [6,24]. We analyzed The Cancer Genome Atlas (TCGA) data and show that TNBC has elevated levels of c-MYC, PD-L1, and STAT5A/5B expression (Supplementary Figure S9A–E). To validate the loss of CD274 (PD-L1), we found the loss of PD-L1 transcript by the 3′UTRMYC1-18 (Figure 2H). We stained the primary TNBC tumors, treated and control, with anti-PD-L1 by immunohistochemistry; the positive PD-L1 controls were normal human tonsil. We found a near total loss of PD-L1 in the tumor-bearing animals that received 3′UTRMYC1-18 (Figure 4A) and mild levels of PD-L1 in the 3′UTRMYC2-3- and 1-14-treated tumors (Figure 4A), whereas the untreated controls had very high levels of PD-L1 (Figure 4A). Quantification of the PD-L1 in the treated versus untreated controls showed that the PD-L1 was significantly reduced in the treated group compared to the controls (Figure 4B). Moreover, we quantified c-MYC and PD-L1 expression in a head-to-head comparison between the treated and the untreated groups. We found that a loss of PD-L1 in the treated groups followed the loss of c-MYC in the same tumors. And, the untreated groups with a high c-MYC expression also had a high PD-L1 expression (Figure 4C). Pearson correlation analysis shows a linear relation between c-MYC and PD-L1 expression (*p*-value = 0.0071, Pearson correlation test, R^2^ = 0.925, Figure 4D). This finding demonstrates that the loss of c-MYC led to the loss of PD-L1 following our treatment, suggesting that c-MYC may control PD-L1 expression. It is known that c-MYC binds the promoter of PD-L1 to control it [22,25,26]. The findings support that the direct destabilization and degradation of c-MYC at both the transcript and protein levels leads to the loss of PD-L1 transcript and protein.

It is of interest to know other regulators of PD-L1 in addition to c-MYC. So, we sought to understand if there is a correlation between STAT5A/B and PD-L1. We quantified the STAT5A/5B and PD-L1 expressions in a head-to-head comparison between the treated and the untreated tumors. We found that the loss of STAT5A/5B in the treated responsive tumors followed the loss of PD-L1 (Figure 4E), and again, in the untreated controls, STAT5A/5B and PD-L1 expression were concomitantly high (Figure 4E). The Pearson correlation analysis between STAT5A/5B and PD-L1 expressions found a high correlation (*p*-value = 0.0061, Pearson correlation test, R^2^ = 0.97, Figure 4F). This finding could be interpreted as a strong functional relationship between PD-L1 and STAT5A/5B and could support that STAT5A/5B might be a regulator of PD-L1. We extended our analysis to ascertain if there is a functional correlation between c-MYC, STAT5A/5B, and PD-L1 in the responsive tumors to our therapy. Multivariate correlation analysis indeed supports a functional relationship between the three molecules (at R^2^ = 0.97, Figure 4G), and we speculate that a higher complex of c-MYC-STAT5A/5B might be regulating PD-L1 expression by binding on its promoter.

### 3.3. Nonresponsive Metastatic TNBCs Are Driven by c-MYC-Independent STAT5A/5B and PD-L1 Expression

The 3′UTRMYC1-18- and 1-14-treated tumor-bearing animals inhibited metastatic tumors by 80% and 60%, respectively (Supplementary Figure S10A,B). To understand the molecular mechanism of metastasis inhibition, we stained the treated and control tissues with antibodies against c-MYC and STAT5A/5B and found that in the tissues where we inhibited metastasis significantly, they lost c-MYC compared to the controls (Supplementary Figure S10C,D); however, the STAT5A/5B expression is on (Supplementary Figure S10E,F). We conclude that the inhibited metastatic tumors have c-MYC-STAT5A/B interaction controlled. But, for the 20% and 40% of the tumors (Supplementary Figure S10G) not responsive to 3′UTRMYC1-18 and 1-14 treatment, respectively, they are under the influence of STAT5A/5B independently of the c-MYC. This agrees with the idea that c-MYC and STAT5A/B have a super-enhancer interaction in tumorigenesis [27] and implicates STAT5A/B as a driver of metastasis in TNBC for the 20–40% of tumors that escape c-MYC-dependent treatment.

To understand the molecules active in the nonresponsive metastatic TNBC, we stained the metastatic tumors with anti-PD-L1 and found that the metastatic tumors from the untreated groups had extremely high to moderate levels of PD-L1 expression, while those from the treated groups had mild to low levels of PD-L1 expression (Supplementary Figure S13A,B). Subsequently, to understand the correlation between c-MYC and PD-L1 in these metastatic tissues, the expressions of c-MYC and PD-L1 were compared. We found that there is no correlation between these molecules in the metastatic tissues (Supplementary Figure S13C,D). Also, there is no functional correlation between STAT5A/5B and PD-L1 expressions in the metastatic tissues (Supplementary Figure S13E,F). Lastly, we examined for correlation between the three molecules, and we found a less strong correlation between c-MYC-STAT5A/5B and PD-L1 expressions in the metastatic tissues than in the responsive primary tumor (Supplementary Figure S13G). These results suggest that the metastatic tissues are not driven by the c-MYC-STAT5A/5B-PD-L1 interaction found in the primary tumors, but rather by an independent STAT5A/5B signal.

### 3.4. IO-Nanocages Delivered Destabilized c-MYC Constructs to Tumors, Leading to Effective Targeting of c-MYC in the Responsive TNBC

The IO-nanocages have effectively delivered therapeutic molecules and RNAs to tumor sites (Figure 1, Figure 2 and Figure 3) [15,16,28,29]. To determine the nanocages’ delivery of the constructs to tumors, we stained the tumor tissues with Prussian blue, which labeled Fe elements. By comparing the positive control primary tumors treated by IO-nanocages with vectors (Supplementary Figure S14B) with the negative control (non-treated tumors) (Supplementary Figure S14A), the constructs 3′UTRMYC2-3, 1-18, and 1-14 were found to be robustly delivered into the tumors that responded to the treatment (Supplementary Figure S14C–E), with the relative abundance quantified (Supplementary Figure S14F). We extended the analysis to the metastatic tumors from the treated and the untreated groups, and we found no IO-nanocages in the metastatic tissues from the control groups, including WT clavicular bone metastatic tumors (Supplementary Figure S14G), the metastatic tumors treated by only IO-nanocages (Supplementary Figure S14H), and bone tumor metastatic tumors treated by IO-nanocages with vectors (Supplementary Figure S14I). In the metastatic tumors from the treated group, we found a very small amount of IO-nanocages (Supplementary Figure S14J–L, also quantified in Supplementary Figure S14M). These results are consistent with the outcome that IO-nanocages delivered the therapeutic cargo with a durable inhibition of tumor growth and metastasis.

### 3.5. IO-Nanocage Package Delivered Destabilized c-MYC Constructs, Effectively Targeted c-MYC-STAT5A/5B-PD-L1 in the Lungs, and Inhibited Distant Organ Lung and Liver Metastasis

Distant organ metastasis is a major killer of cancer patients, and TNBC has a preponderance to metastasize to the lungs, liver, brain, and bone. And, there are few to no therapeutics that effectively target metastasis. To evaluate the inhibition of distant organ metastasis, we performed H&E, anti-c-MYC, PD-L1, STAT5A/5B, and IO-nanocage staining of lung tissues from the treated and untreated groups. The lungs from the no-intervention group lost their parenchymal architecture due to metastasis (Supplementary Figure S15A), with a very high level of PD-L1 expression (Supplementary Figure S15B); there was no presence of IO-nanocages, as a negative control (Supplementary Figure S15C, quantified in the Supplementary Figure S15D). We found a loss of lung parenchyma architecture in the control of IO-nanocages with vectors (Supplementary Figure S15E), with elevated levels of PD-L1 (Supplementary Figure S15F), and IO-nanocages were found in the tumors as a positive control (Supplementary Figure S15G–H). The lungs from the treated groups still had some of their lung parenchyma architecture intact (Supplementary Figure S15I,M,Q), with a low level of PD-L1 expression (Supplementary Figure S15J,N,R), and IO-nanocages were found in their lung parenchyma (Supplementary Figure S15K,L,O,P,S,T). The most effective construct, the 3′UTRMYC1-18 group, had the most IO-nanocages in the lung parenchyma, which is consistent with the reduced level of c-MYC and STAT5A/5B in the lungs of mice treated with 3′UTRMYC1-18 (Supplementary Figure S15A,B). Taken together, this demonstrated that our therapy reached distant-organ lungs and reduced c-MYC, STAT5A/5B, and PD-L1 expression, which inhibited metastasis and preserved the lung parenchyma in the treated group.

Moreover, we found that the 3′UTRMYC1-18 inhibited liver metastasis and preserved the liver parenchyma compared to the controls (Supplementary Figure S16A,C,E,G). The IO-nanocages were robustly delivered in the liver of the 3′UTRMYC1-18-treated mice compared to the controls (Supplementary Figure S16B,D,F,H). In the kidneys, we found that 3′UTRMYC1-18 preserved kidney tubule cellular architecture compared to controls (Supplementary Figure S17A–C). These data validate our findings on the inhibition of primary tumors and metastasis to distant organs.

### 3.6. The Engineered Destabilized c-MYC Degrades the Endogenous MYC by Overwriting Its mRNA Message through EEF2 Upregulation and Increased XRN1 and CNOT1 Expression

To understand mechanistically how the engineered destabilized constructs overwrote the endogenous c-MYC encoded messages, we labeled the control and destabilized cells with 4SU (thiouridine) 27 and collected RNAs at four time points to assay the expression of the endogenous and destabilized c-MYC in the same cells and at the same time point (Supplementary Figure S18A). We found that the 3′UTRMYC1-18 outcompeted the mRNA of endogenous MYC (Supplementary Figure S18B) by a factor of 4-fold in the transcript production, and this production of infidel-destabilizing transcript triggered nonsense-mediated decay through the upregulation of UPF1 (*p* = 6.1 × 10^−8^, FDR 5.2 × 10^−6^) (Supplementary Figure S12A–E). To find the ribosomal machinery and how RNA binding proteins (RBPs) orchestrating the 3′UTRMYC1-18 outcompeted the endogenous MYC, we triaged three different RNA Seq data of destabilized cells from different models and found EEF2 (Supplementary Figure S18C). To validate this, we performed Western blotting on the 4SU time-treated samples, both in the wildtype and treated cells. The EEF2 protein expression was relatively higher in 3′UTRMYC1-18 at time points from 30 min to 3 h compared to the wildtype cells (Supplementary Figure S18D–G). We assayed for the expression of 5′-3′ and 3′-5′ exonucleases that degrade the destabilized transcript, and we found that both XRN1 and CNOT1 were markedly upregulated in the 3′UTRMYC1-18-treated cells as compared to the controls (Supplementary Figure S18I–J).

## 4. Discussion

This is a first example of engineering mRNA-stabilizing poly U sequences to unstable forms and overwriting the endogenous c-MYC mRNA message simultaneously. The administration of the constructs complexed with IO-nanocages to mice bearing TNBC caused no adverse reactions, and it did not kill normal epithelial cells expressing basal c-MYC. The IO-nanocages distributed the constructs to the tumors, liver, and lungs to achieve therapeutic efficacy. The constructs were found safely in the non-coding region of the genome and not in oncogenic hotspots. Our dosing regimen relies on the logic of getting the construct to integrate first, with evident tumor reduction, and then spacing out the dosing. The three constructs were effective in vitro but only 3′UTRMYC1-18 was highly effective in vivo in achieving primary endpoint tumor reduction and inhibition of metastasis even in the brain (Supplementary Figure S20A–L). Differential pharmacokinetics might explain this, and further studies are ongoing on this and their impacts on immune cells.

We have uncovered a new paradigm, which is that the primary tumor responsive to this therapy lost c-MYC-STAT5A/5B-PD-L1 interaction upon the treatment, and resistance is driven by STAT5A/5B. Thus, the combination of our MYC constructs with anti-PD-L1 and anti-STAT5A/5B might address PD-L1-negative TNBCs (NCT03164993), which are more prevalent. The treated animals survived for more than 16–17 days, which is equivalent to 680 days, or 22 months, for humans). One mouse day equals forty human days. Atezolizumab plus paclitaxel offered an additional 7.5 months of survival versus placebo plus paclitaxel, while atezolizumab plus chemotherapy achieved 20.6 months of survival vs. placebo plus chemotherapy, which resulted in 19.8 months of survival benefit (Impassion 031, Impassion 130, NCT02489448, MK-3475-119/KEYNOTE-119). Thus, the 22-month survival benefit of these constructs/anti-PD-L1/STAT5A/5B could be superior to the standard of cancer care.

This technology is a platform to safely engineer transcripts and therapeutic genes as shown for c-MYC in cancer treatments [12].

## 5. Conclusions

We report the development of mRNA overwriting therapy based on the engineered destabilization of the poly U stabilizing elements on the 3′UTR of MYC, which triggered nonsense-mediated decay to degrade the MYC transcript and protein, leading to the inhibition of c-MYC-driven TNBC and other cancers. The drugs were well tolerated and safe for the treated animals. This novel therapeutic approach opens a novel avenue to safely target diseased mRNA across many diseases.

## 6. Patents

CUA, KS, OOO, and HM have filed several patents relating to this technology.

## Data Availability

The Sanger sequencing data for the identification of 3′UTR c-MYC poly U sequences and RNA Seq FASTQ files are deposited on Zenodo (DOI: 10.5281/zenodo.10030728). There were no codes developed in this study. The constructs generated in this study will be distributed through CUNY and UTR Therapeutics Inc.

## Supplementary Materials

The following supporting information can be downloaded at https://www.mdpi.com/article/10.3390/cancers16152663/s1, Supplementary Figure S1. Identification of the stabilizing mRNA poly U sequences on the 3′UTR of c-MYC across many cancers. A. Gel image of c-MYC 3′UTR amplified from the cDNA of the MCF7, BT474, MDA MB231, and the T47D, marked in black asterisks. B. Electropherogram of MCF7 3′UTR with poly U sequences marked in black. C. Electropherogram of BT474 3′UTR with poly U sequences marked in black. D. Electropherogram of MDA MB231 3′UTR with poly U sequences marked in black. E. Electropherogram of T47D 3′UTR with poly U sequences marked in black. F. Bar chart showing the zeta potential (mV) of the neat IO-nanocage, neat DNA constructs, and DNA-IO-nanocage. G. Bar chart showing the size (nm) of the neat IO-nanocage, neat DNA constructs, and DNA-IO-nanocages, determined by DLS. Supplementary Figure S2. The stabilizing mRNA poly U sequences are 99% conserved on the 3′UTR of c-MYC across many cancers. A. Multi-alignment of poly U sequences of the MCF7, BT474, MDA MB231, and the T47D. Supplementary Figure S3. c-MYC 3′UTR cDNA converted to mRNA sequences with the stabilizing poly U sequences engineered to the destabilizing elements, underlined in black. A. The alignment of the 3′UTR of the WT c-MYC and the engineered destabilized c-MYC with sequences that were changed. Supplementary Figure S4. Design of destabilized c-MYC 3′UTR with DCP1A promoter, BstB1, BamH1 restriction sites, and plasmid vector design. A. Schematic illustration of component sequences used in the design of destabilized 3′UTR c-MYC. B. Schematic illustration of the plasmid vector components. C. Western blot of caspase 7 and GAPDH in the controls and in cells that received the destabilized constructs. D. Bar chart shows the quantification of caspase 7 protein expression normalized against GAPDH in the treated and control groups (*** *p* < 0.001, two-tailed *t*-test). E. Tumor sizes of the different groups: the controls and the treatment groups. Supplementary Figure S5. Sequence of cloned destabilized c -MYC constructs 3′UTRMYC2-3, 1-18, and 1-14. A. Schematic illustration of the cloned 3′UTRMYC2-3 mapped to the c-MYC 3′UTR. B. Schematic illustration of the cloned 3′UTRMYC1-18 mapped to the c-MYC 3′UTR. C. Schematic illustration of the cloned 3′UTRMYC1-14 mapped to the c-MYC 3′UTR. Supplementary Figure S6. The destabilized ARE 3′UTR of c-MYC specifically targets MYC-dependent kinases and transcription factors in TNBC. A. Bar charts show the intensity of p38a, JNK1/2/3, STAT5A/5B, GSK3A/B, PDGFRB, and LCK in BT474 clone 5 WT (black), BT474 clone 5 vectors (blue), BT474 clone 5 desARE3′UTRERBB2-3 (red), and BT474 clone 5 3′UTRMYC2-3 (green) **** *p*-value < 0.0001, two-tailed *t*-test. B. Western blot shows the c-MYC, STAT5A/5B, and the GAPDH protein expression in the control and the destabilized cells (N = 6). C. Quantification of the c-MYC and STAT5A/5B protein expression normalized against GAPDH (*** *p* < 0.001, two-tailed *t*-test). D. Heat map shows the expression levels of c-MYC and interaction partners in MDA MB231 WT, vector, 3′UTRMYC1-18, and normal female mammary gland gene expression from ENCODE. E. Bar charts show the ERBB2 mRNA expression in MDA MB231 WT, vector, and 3′UTRMYC1-18. F. Bar charts show the EGFR, VEGFA, ABL1, RELA, and RELB mRNA expression in MDA MB231 WT, vector, and 3′UTRMYC1-18. Supplementary Figure S7. The engineered destabilized c-MYC constructs do not degrade c-MYC or kill normal epithelial cells. A. Western blot shows c-MYC and GAPDH protein expression in normal epithelial RWPE1 WT, vector, 3′UTRMYC1-14, 3′UTRMYC1-18, and 3′UTRMYC2-3 treated cells for 4 days. B. Bar chart shows quantification of c-MYC normalized against GAPDH for gel image from A. C. Light microscopic image of normal epithelial cells, RWPE1 WT cells, and cells treated with vector, 3′UTRMYC1-14, 3′UTRMYC1-18, and 3′UTRMYC2-3 for 4 days (N = 4). D. Western blot shows c-MYC and GAPDH protein expression in normal epithelial cells, RWPE1 WT cells, and cells treated with vector, 3′UTRMYC1-14, 3′UTRMYC1-18, and 3′UTRMYC2-3 for 8 days. E. Bar chart shows quantification of c-MYC normalized against GAPDH for gel image from D. F. Light image of normal epithelial cells, RWPE1 WT cells, and cells treated with vector, 3′UTRMYC1-14, 3′UTRMYC1-18, and 3′UTRMYC2-3 for 8 days (N = 4). G. Western blot shows c-MYC and GAPDH protein expression in NCI H1975 vector, desARE3′UTRERBB2-1, desARE3′UTRERBB2-1, desARE3′UTRERBB2-3, desARE3′UTRERBB2-30, and NCI H1975 trastuzumab-treated cells. H. Bar chart shows quantification of c-MYC normalized against GAPDH for gel image from G. I. Bar chart shows quantification of c-MYC mRNA expression normalized against GAPDH in normal epithelial cells, RWPE1 WT cells, and cells treated with vector, 3′UTRMYC2-3, 3′UTRMYC1-18, and 3′UTRMYC1-14, for 8 days (N = 4). Supplementary Figure S8. Schematic depiction of the animal experiment. A. Schematic illustration of the animal experiment, tumor implantation, randomization, construct administration, and daily tumor measurement and weighing. B. Chart shows the daily summation of tumor volume recording for the control and treatment groups. C. Chart shows the monitoring of weight measurements of animals bearing tumors, both controls and the untreated group. Supplementary Figure S9. Gene expression profile of CD274 (PD-L1), MYC, and STAT5A/5B in (ER−/HR−/HER2−), (ER+/HR+/HER2+), and (ER+/HR−/HER2−). A. Bar chart shows the CD274 (PD-L1) expression in different subtypes of breast cancer (ER−/HR−/HER2−), (ER+/HR+/HER2+), and (ER+/HR−/HER2−). B. Bar chart shows the c-MYC expression in different subtypes of breast cancer (ER−/HR−/HER2−), (ER+/HR+/HER2+), and (ER+/HR−/HER2−). C. Bar chart shows the STAT5A expression in different subtypes of breast cancer (ER−/HR−/HER2−), (ER+/HR+/HER2+), and (ER+/HR−/HER2−). D. Bar chart shows the STAT5B expression in different subtypes of breast cancer (ER−/HR−/HER2−), (ER+/HR+/HER2+), and (ER+/HR−/HER2−). E. Kaplan–Meier survival curve for c-MYC (low) and c-MYC (high) expression in TNBC. Supplementary Figure S10. IO-nanocages delivered constructs and inhibited metastatic TNBC by downregulating c-MYC; however, the unresponsive tumors were driven by c-MYC-independent STAT5A/5B expression. A. Metastatic tumor images; sizes from the different controls and the treatment groups (N = 2 WT, N = 3 nanocage only, N = 4 IO-nanocage plus vector, N = 4 3′UTRMYC2-3, N = 1 3′UTRMYC1-18, N = 2 3′UTRMYC1-18). B. Quantification of metastatic tumors from the controls and the treated groups. C. Immunofluorescence images of metastatic tumors from the controls and the treatment groups stained with c-MYC (red), DAPI nuclei (blue), groups (N = 2 WT, N = 3 nanocage only, N = 4 IO-nanocage plus vector, N = 4 3′UTRMYC2-3, N = 1 3′UTRMYC1-18, N = 2 3′UTRMYC1-18). D. Quantification of c-MYC in metastatic tumors from the controls and the treated groups by immunofluorescence. * *p* < 0.044 (WT vs. 3′UTRMYC2-3), * *p* < 0.03 (WT vs. 3′UTRMYC1-18 and 1-14). E. Immunofluorescence images of metastatic tumors from the control and the treatment groups stained with STAT5A/5B (red), DAPI nuclei (blue), groups (N = 2 WT, N = 3 IO-nanocage only, N = 4 nanocage plus vector, N = 4 3′UTRMYC2-3, N = 1 3′UTRMYC1-18, N = 2 3′UTRMYC1-18). F. Quantification of STAT5A/5B in metastatic tumors from the controls and treated groups by immunofluorescence. *p* = ns. Supplementary Figure S11. Migration assay of the treated and untreated MDA MB231. A. Microscopic images of migration of the cells from the MDA MB231WT, vector, 3′UTRMYC2-3, 1-18, and 1-14 at 0 h, 24 h, 48 h, and 72 h. B. Bar charts show the quantification of wound healing in the control and treated groups (** *p* < 0.01, **** *p* < 0.0001, two-tailed *t*-test). Supplementary Figure S12. The engineered destabilized 3′UTR of c-MYC is sequence-specific and works through nonsense-mediated decay as designed. A. Upregulated GO terms in the destabilized cells carrying 3′UTRMYC1-18. B. Heat map of UPF1 mRNA expression in the WT, vector-, and 3′UTRMYC1-18-treated-cells. C. Western blot shows DCP1A and the GAPDH in the control and treated cells. D. Bar charts show the head-to-head comparison of DCP1A and c-MYC protein expression normalized against GAPDH in the same cells, both treated and untreated (*** *p* < 0.001, **** *p* < 0.0001, two-tailed *t*-test). E. DCP1A mRNA expression from the controls and the 3′UTRMYC1-18-treated cells. Supplementary Figure S13. The nonresponsive metastatic TNBC tumors are driven by STAT5A/5B signal independently of c-MYC. A. Immunohistochemistry staining of PD-L1 in metastatic tumors from the control and treated groups; PD-L1 (brown), DAPI nuclei (blue). (N = 2 WT, N = 3 nanocage only, N = 4 IO-nanocage plus vector, N = 4 3′UTRMYC2-3, N = 1 3′UTRMYC1-18, N = 2 3′UTRMYC1-14). B. Quantification of PD-L1 in metastatic tumors from the control and treated groups,* *p* < 0.01 (WT vs. 3′UTRMYC2-3 + nanocage, 1-18 and 1-14). C. Head-to-head quantification of PD-L1 and c-MYC in the same metastatic tumors from the control and treated groups. ** *p* < 0.001 (WT vs. 3′UTRMYC2-3 + nanocage, 1-18 and 1-14). D. Graph shows the correlation analysis between PD-L1 and c-MYC in the metastatic tumors, R^2^ = 0.42, *p* = ns. E. Head-to-head quantification of PD-L1 and STAT5A/5B in metastatic tumors from the control and treated groups, *p* = ns for STAT5A/5B (WT, nanocage + vector vs. 3′UTRMYC2-3 + nanocage, 1-18 and 1-14). F. Graph shows the correlation analysis between PD-L1 and STAT5A/5B in the metastatic tumors, R^2^ = −0.06, *p* = ns. G. Table shows the multiple correlation analysis between c-MYC, STAT5A, and 5B -PD-L1 in the metastatic tumors from the control and treated groups. Supplementary Figure S14. Nanocage detection in primary and metastatic treated tumors. A. WT no-intervention primary tumor group, no IO-nanocage detected (N = 5). B. IO-nanocage + vector-treated primary tumor group, IO-nanocage detected (red arrow) (N = 2). C. 3′UTRMYC2-3-treated primary tumor group, IO-nanocage detected (red arrow) (N = 4). D. 3′UTRMYC1-18-treated primary tumor group, IO-nanocage detected (red arrow) (N = 2). E. 3′UTRMYC1-14-treated primary tumor group, IO-nanocage detected (red arrow) (N = 2). F. Quantification of IO-nanocages detected in the primary tumor. G. WT clavicular bone metastatic tumor group, no IO-nanocage detected (N = 2). H. Nanocage-only metastatic tumor group, no IO-nanocage detected (N = 3). I. Nanocage + vector metastatic tumor group, no IO-nanocage detected (N = 4). J. 3′UTRMYC2-3-treated metastatic tumor group, IO-nanocage detected (red arrow) (N = 4). K. 3′UTRMYC1-18-treated metastatic tumor group, IO-nanocage detected (red arrow) (N = 1). L. 3′UTRMYC1-14-treated metastatic tumor group, IO-nanocage detected (red arrow) (N = 2). M. Quantification of nanocages detected in the metastatic tumor. Supplementary Figure S15. IO-nanocage package delivered destabilized c-MYC constructs to the lungs, targeting c-MYC-STAT5A/5B-PD-L1 in the lungs, and inhibited distant organ lung metastasis. A. H&E stain of the lung metastatic tumor from the MDA MB231 no-intervention group (N = 3). B. PD-L1 stain of lung metastatic tumor from the MDA MB231 no-intervention group (N = 3). C. Prussian blue stain of lung metastasis tumor from the MDA MB231 no-intervention group (N = 3). D. The quantification of PD-L1 and nanocages, no IO-nanocage detected in the MDA MB231 no-intervention group. E. H&E stain of the lung metastatic tumor from MDA MB231 of the vector plus IO-nanocage-only intervention group (N = 2). F. PD-L1 stain of lung metastatic tumor of IO-nanocage plus vector intervention group (N = 2). G. Prussian blue stain of lung metastatic tumor of IO-nanocage plus vector intervention group, IO-nanocages marked in red arrow (N = 2). H. Quantification of PD-L1 and nanocages. I. H&E stain of the lung metastatic tumor from the MDA MB231 of 3′UTRMYC2-3 intervention group (N = 4). J. PD-L1 stain of the lung metastatic tumor from MDA MB231 of 3′UTRMYC2-3 intervention group (N = 4). K. Prussian blue stain of lung metastatic tumor in the 3′UTRMYC2-3 intervention group, IO-nanocages marked in red arrow (N = 4). L. Quantification of PD-L1 and nanocages. M. H&E stain of the lung metastatic tumor from the MDA MB231 of 3′UTRMYC1-18 intervention group (N = 4). N. PD-L1 stain of the lung metastatic tumor stain from the MDA MB231 of 3′UTRMYC1-18 intervention group (N = 4). O. Prussian blue stain of the lung metastatic tumor in the 3′UTRMYC1-18 intervention group, IO-nanocages marked in red arrow (N = 4). P. Quantification of PD-L1 and nanocages. Q. H&E stain from the lung metastatic tumor from the MDA MB231 of 3′UTRMYC1-14 intervention group (N = 4). R. PD-L1 stain of the lung metastatic tumor stain from the MDA MB231 of 3′UTRMYC1-14 intervention group (N = 4). S. Prussian blue stain of lung metastasis tumor in the 3′UTRMYC1-14 intervention group, IO-nanocages marked in red arrow (N = 4). T. Quantification of PD-L1 and nanocages. Supplementary Figure S16. IO-nanocage robustly delivered destabilized c-MYC constructs to the liver and inhibited liver metastasis. A. H&E stain of the MDA MB231 no-intervention liver showing hyperchromasis and metastasis (N = 4). B. Prussian blue stain of IO-nanocages in MDA MB231 no-intervention group, no IO-nanocage found (N = 4). C. H&E stain of the MDA MB231 liver treated with IO-nanocage plus vector showing hyperchromasis and metastasis (N = 4). D. Prussian blue stain of IO-nanocages in IO-nanocage plus vector-treated group, IO-nanocage found, marked with red arrow (N = 4). E. H&E stain of the MDA MB231 3′UTRMYC1-18-treated liver showing no hyperchromasis and no metastasis (N = 4). F. Prussian blue stain of IO-nanocages in 3′UTRMYC1-18 plus IO-nanocages-treated group, IO-nanocages were robustly found, marked with red arrow (N = 4). Supplementary Figure S17. H&E stain of the kidney tissues. A. H&E stain of the MDA MB231 kidney no-treatment group (N = 4). B. H&E stain of the MDA MB231 IO-nanocage plus the vector-treated group (N = 4). C. H&E stain of the MDA MB231 IO-nanocage-only treated group (N = 4). D. H&E stain of the MDA MB231 IO-nanocage plus 3′UTRMYC2-3-treated group (N = 4). E. H&E stain of the MDA MB231 IO-nanocage plus 3′UTRMYC1-18-treated group; tissue has normal renal tubular cellular architecture (N = 4). F. H&E stain of the MDA MB231 IO-nanocage plus 3′UTRMYC1-14-treated group (N = 4). Supplementary Figure S18. The engineered destabilized 3′UTR overwrote endogenous MYC by upregulation of EEF2, XRN1, and CNOT1. A. Schematic depiction of 4SU pulse-chase nascent mRNA labeling of the control cell and the cells carrying the destabilized constructs. B. Graph shows time-dependent mRNA expression of the endogenous (blue) and destabilized 3′UTRMYC (orange) in the same cells from 0 h–24 h in the controls and destabilized cells. C. Venn diagrams show EEF2 as intercept between NCI H1975 desARE3′UTRERBB2-3, 30, MDA MB231 3′UTRMYC1-18, and DAOY 3′UTRMYC1-18. D. Western blot of EEF2 and GAPDH protein expression in time scale 0–24 h in wildtype MDA MB231. E. Western blot of EEF2 and GAPDH protein in time scale 0–24 h in 3′UTRMYC1-18. F. Quantification of EEF2 protein expression normalized against GAPDH in time scale 0-24hr in wildtype MDA MB231. G. Quantification of EEF2 protein expression normalized against GAPDH in time scale 0–24 h in 3′UTRMYC1-18. H. Quantification of XRN1 mRNA expression normalized against ActB in WT, vector, and 3′UTRMYC1-18. I. Quantification of CNOT1 mRNA expression normalized against ActB in WT, vector, and 3′UTRMYC1-18. Supplementary Figure S19. Detection of genome integration sites of the destabilized constructs. A. Schematic depiction of the vector constructs containing the destabilizing constructs; the arrows indicate primer positions used in the targeted sequencing. B. Gel image of the constructs amplified in the plasmid vector as well as in tumors treated with the constructs. C. Circos plot with green bars shows the genomic sites of constructs’ integration. Supplementary Figure S20. H&E and Prussian blue stain of the brain tissues. A. H&E stain of the MDA MB231 no-intervention brain tissue, metastasis pointed out by yellow arrow (N = 5). B. Prussian blue stain of MDA MB231 no-intervention brain tissue. No nanocages found (N = 5). C. H&E stain of the MDA MB231 vector-treated brain tissue, metastasis pointed out by yellow arrow (N = 5). D. Prussian blue stain of the MDA MB231 vector-treated brain tissue. No nanocages found (N = 5). E. H&E stain of the MDA MB231 IO-nanocages and vector-treated brain tissue, metastasis pointed out by yellow arrow (N = 5). F. Prussian blue stain of the MDA MB231 IO-nanocages and vector-treated brain tissue. No nanocages found (N = 5). G. H&E stain of the MDA MB231 IO-nanocages 3′UTRMYC2-3-treated brain tissue, no metastasis found (N = 5). H. Prussian blue stain of the MDA MB231 IO-nanocages 3′UTRMYC2-3-treated brain tissue. Nanocages found (N = 5). I. H&E stain of the MDA MB231 IO-nanocages 3′UTRMYC1-18-treated brain tissue; metastasis found marked in yellow arrow (N = 5). J. Prussian blue stain of the MDA MB231 IO-nanocages 3′UTRMYC1-18-treated brain tissue. Nanocages found (N = 5). K. H&E stain of the MDA MB231 IO-nanocages 3′UTRMYC1-14-treated brain tissue; metastasis found marked by yellow arrow (N = 5). L. Prussian blue stain of the MDA MB231 IO-nanocages 3′UTRMYC1-18-treated brain tissue. Nanocages found (N = 5). M. Bar chart shows the quantification of brain metastasis and nanocages in the brain tissues of the control and treated animals (N = 5 mouse brains per group). N. Bar chart shows quantification of kidney metastasis in the treated and control groups (** *p* < 0.01, *** *p* < 0.001, **** *p* < 0.0001, two-tailed *t*-test). Table S1. List of reagents, bacteria strains, cell lines, oligonucleotides and softwares used in the study. File S1: Original western blots.
