# Supplementary material for: The Engineered Drug 3′UTRMYC1-18 Degrades the c-MYC-STAT5A/B-PD-L1 Complex In Vivo to Inhibit Metastatic Triple-Negative Breast Cancer
Source: Cancers (Basel). 2024 Jul 26;16(15):2663. doi: 10.3390/cancers16152663 (PMC11311709; doi:10.3390/cancers16152663)
Supplement: Supplementary file 1 [file cancers-16-02663-s001.zip › File S1. original WB figures.pdf]

2A uncropped

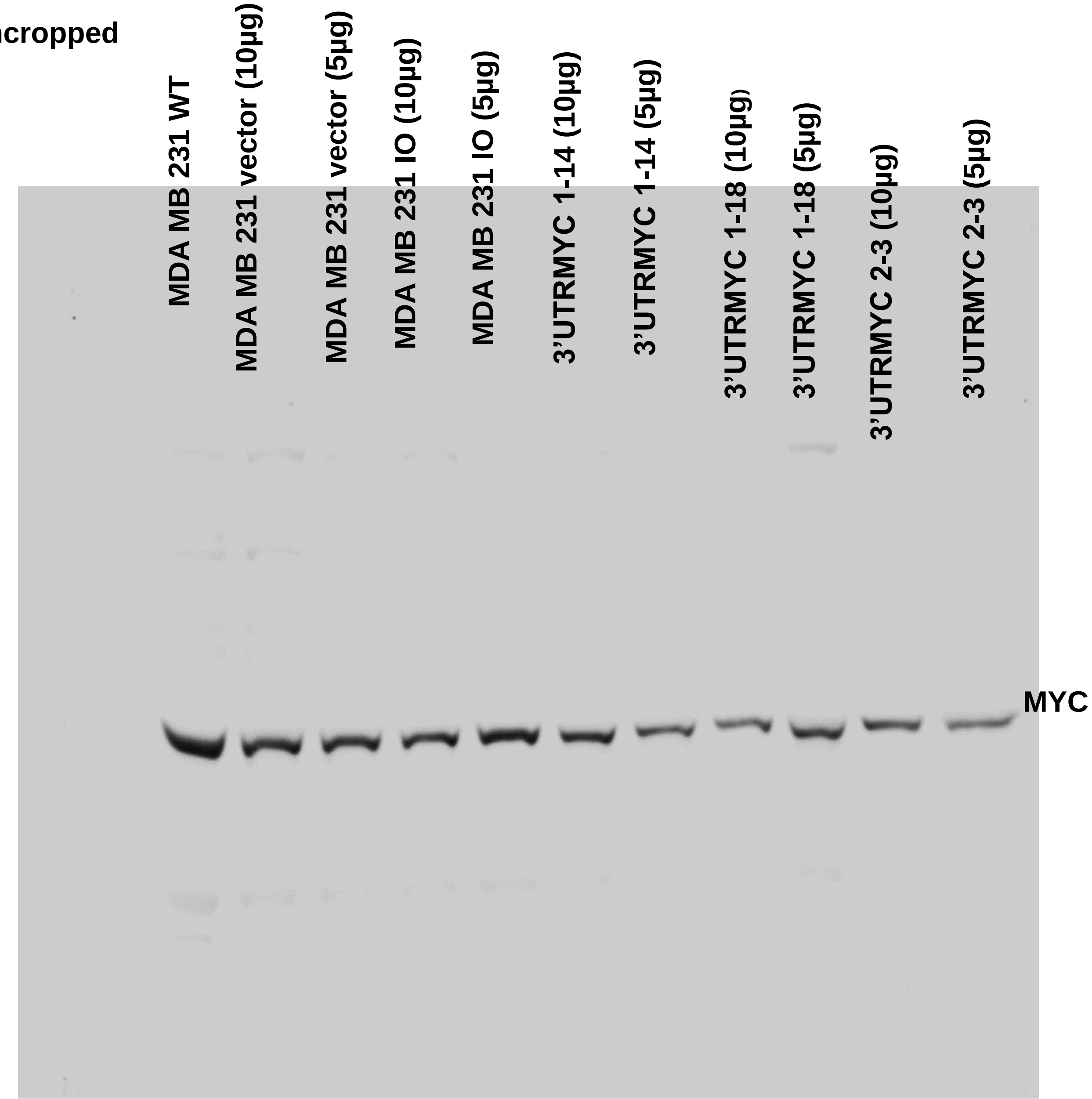

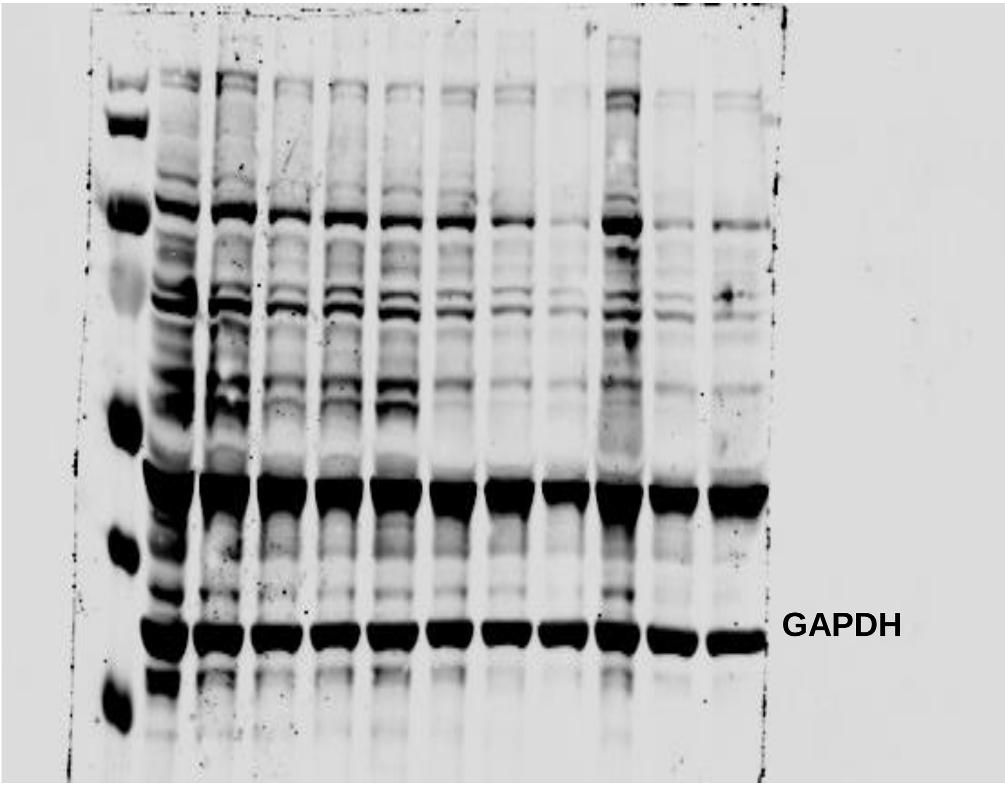

21 uncropped

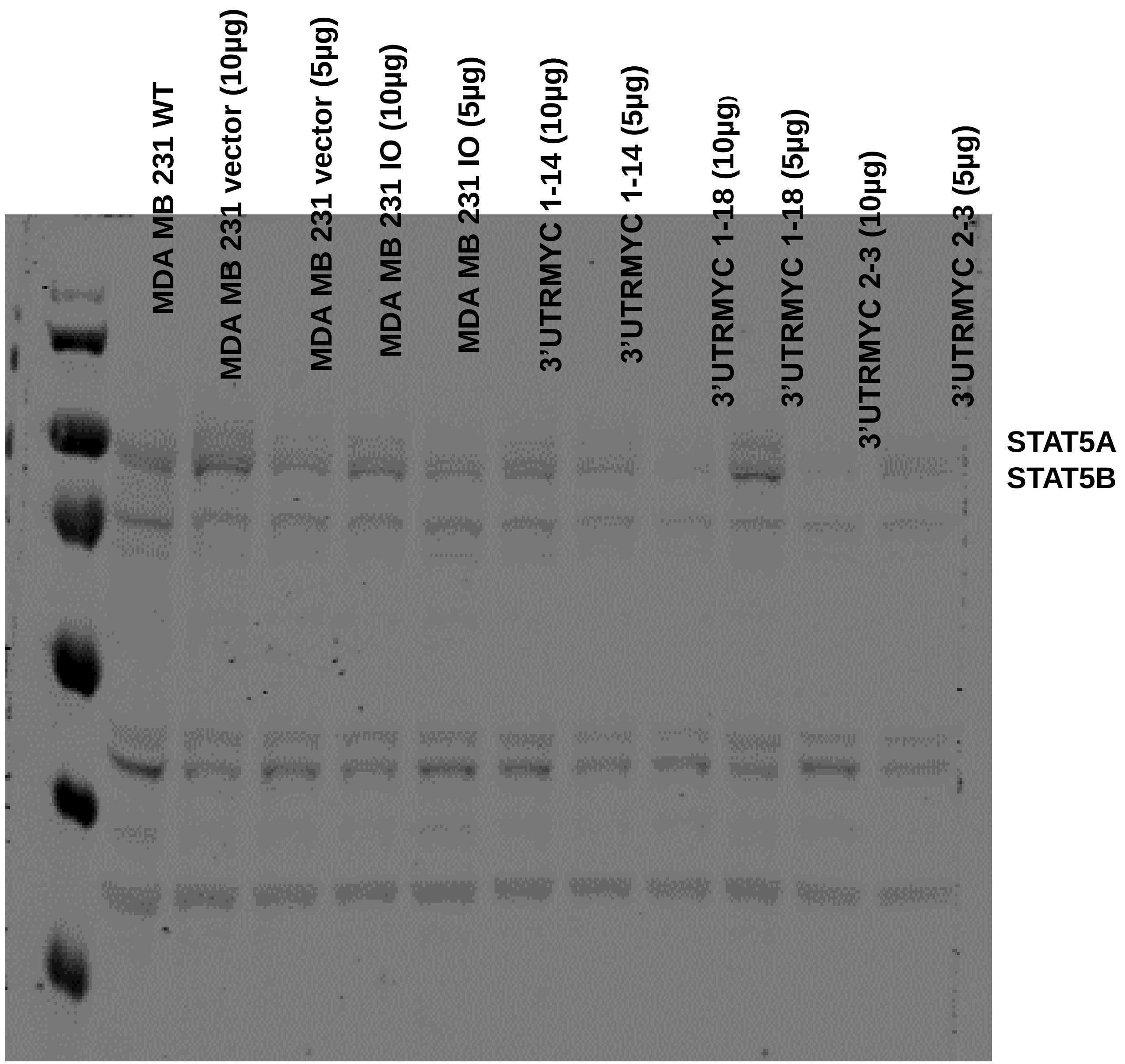

3A uncropped

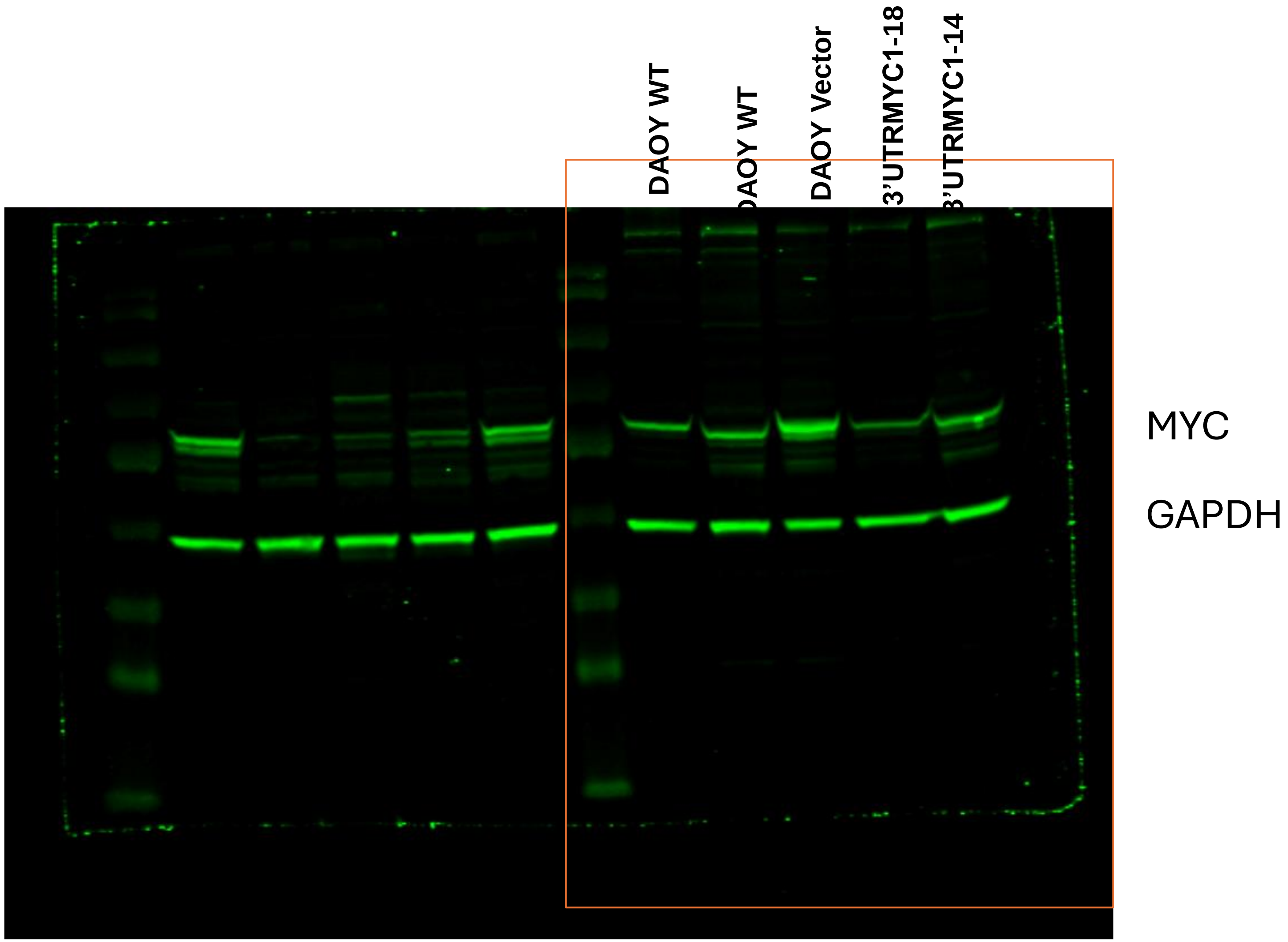

3J uncropped

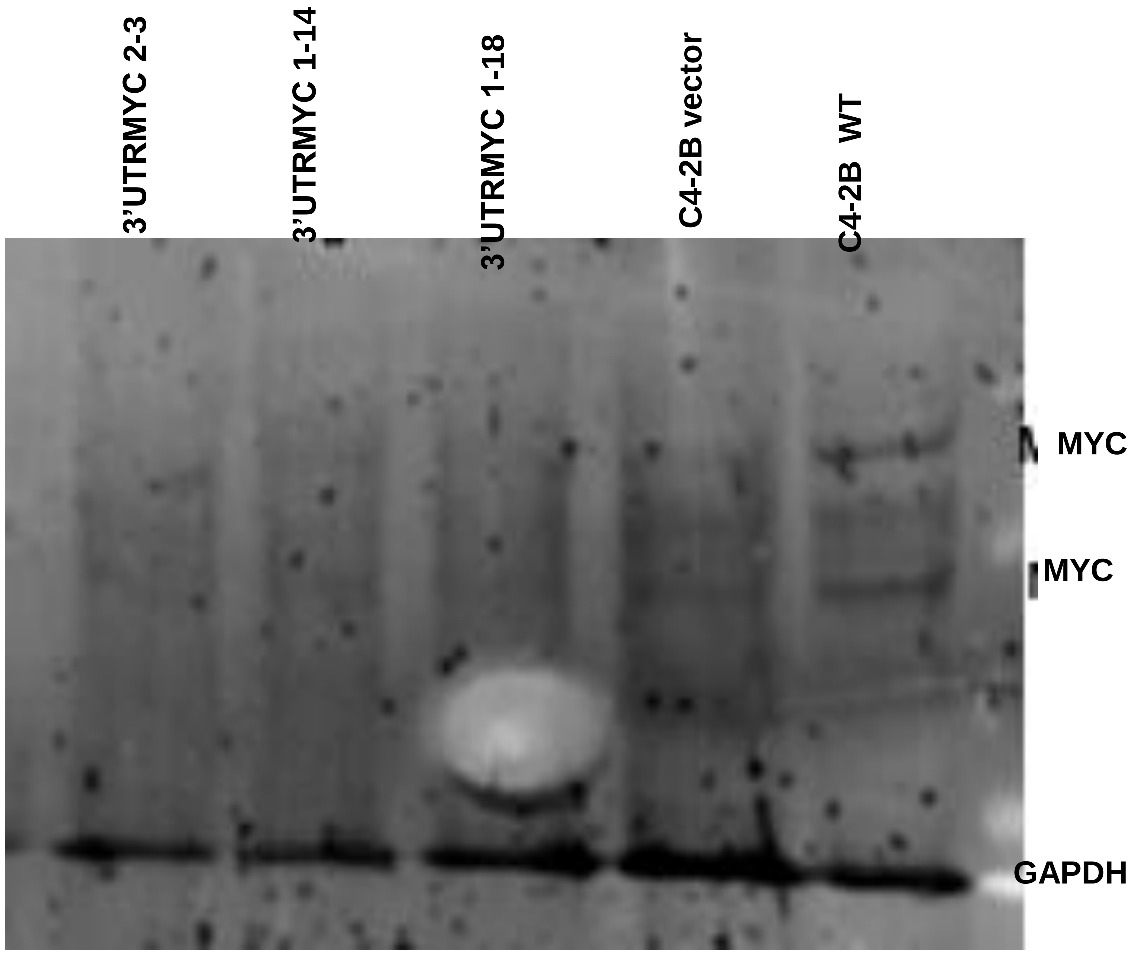

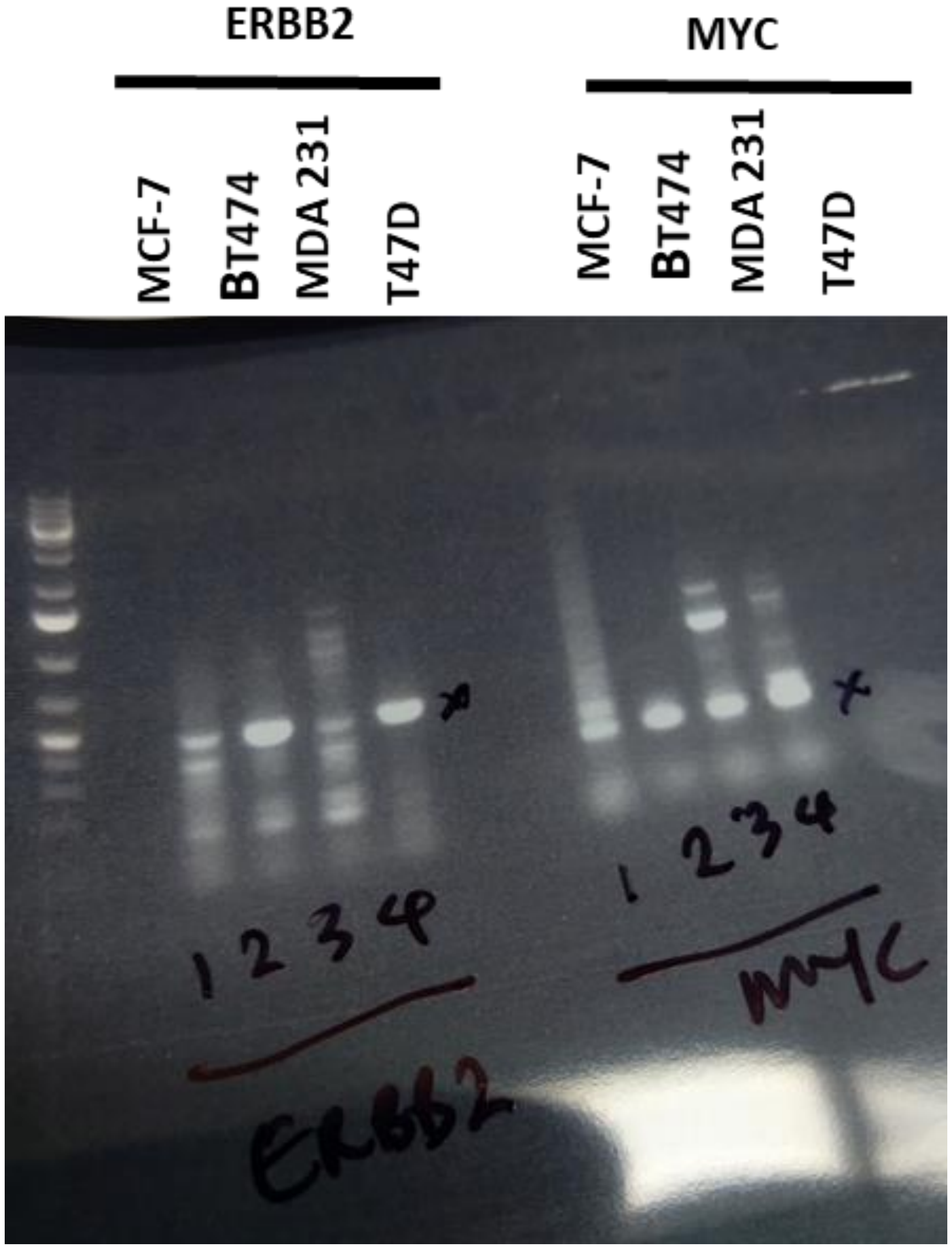

Supplementary Fig 1A uncropped

Supplementary 7A, G uncropped

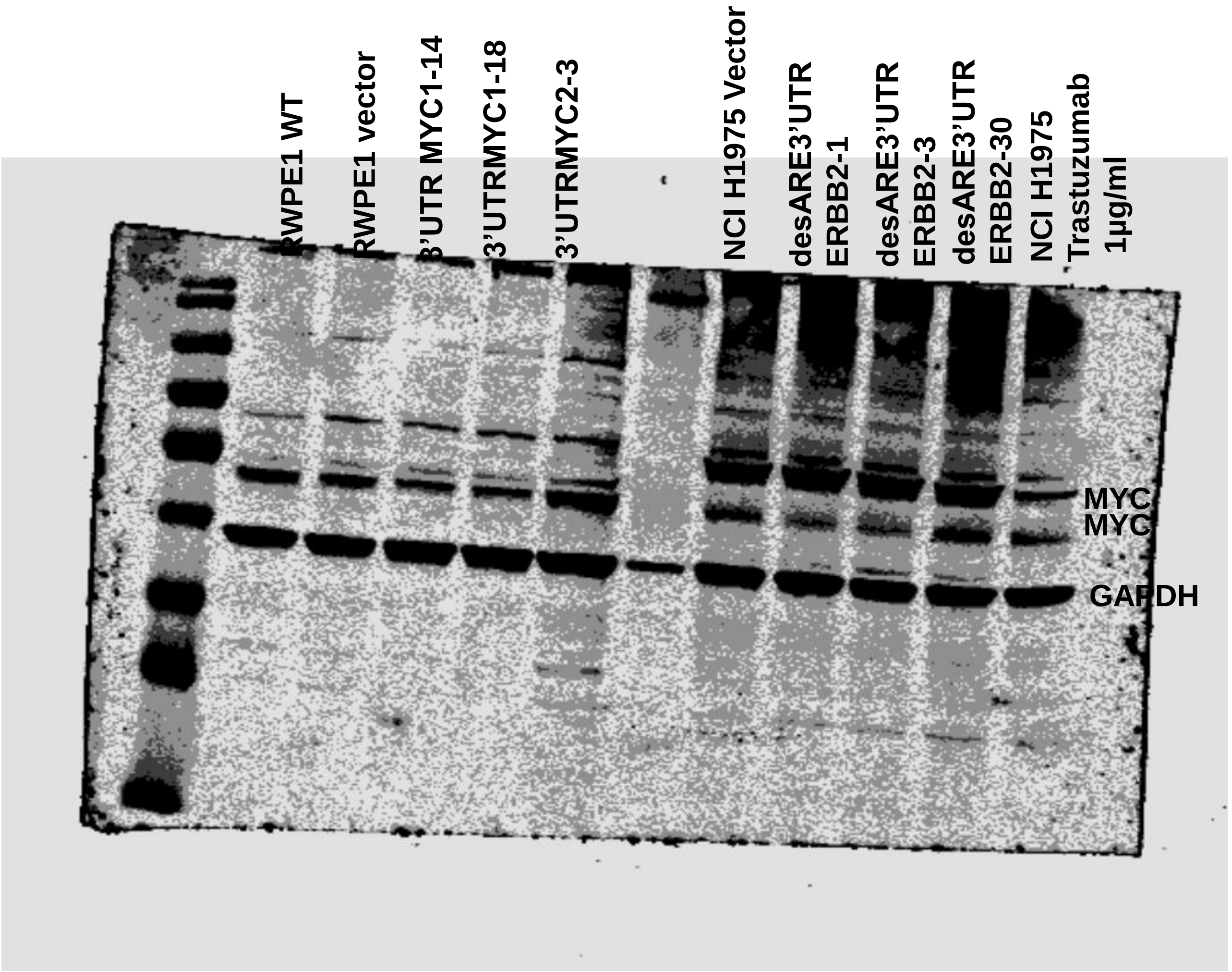

Supplementary 7A, G uncropped

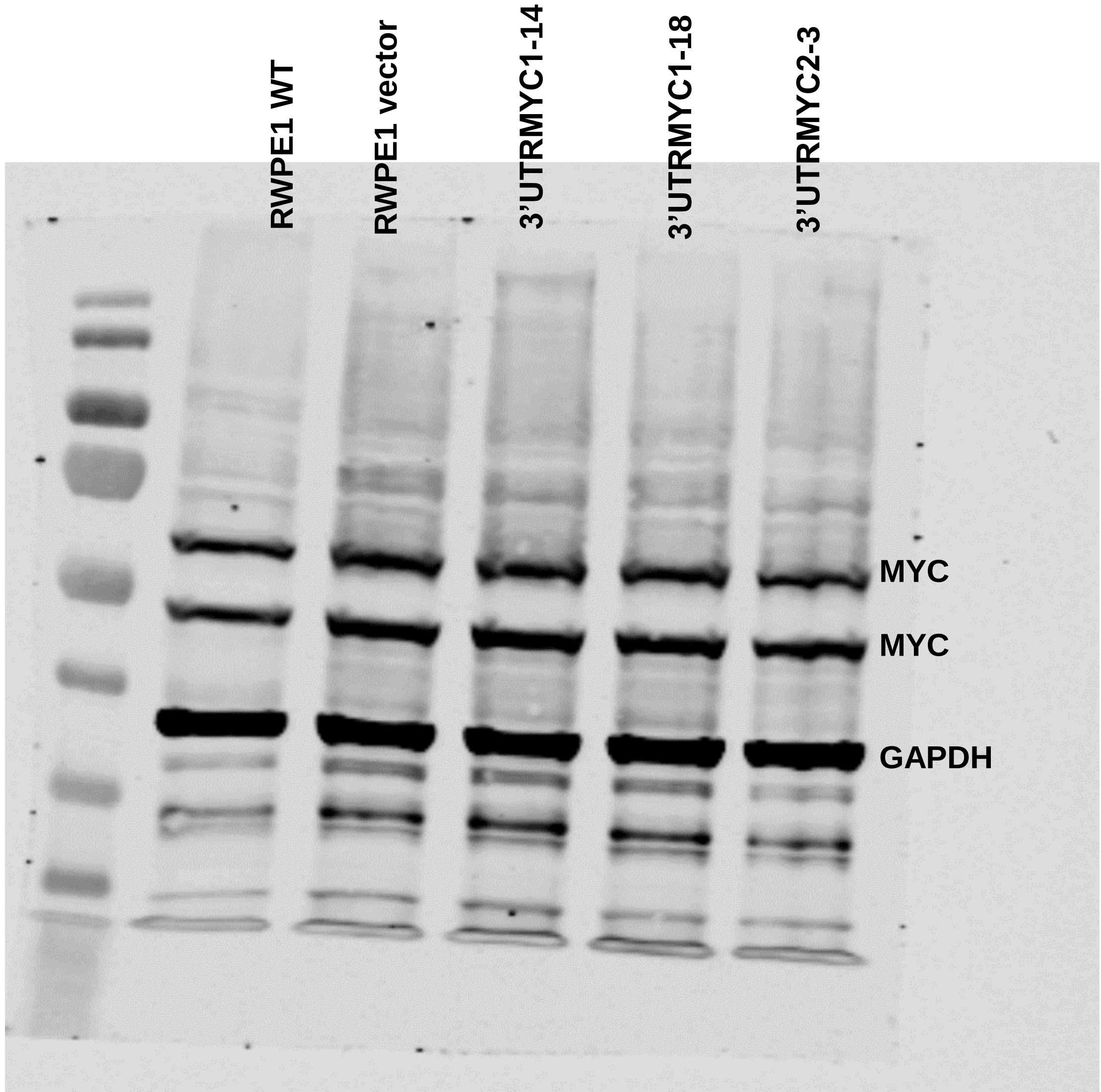

Supplementary 12 C uncropped

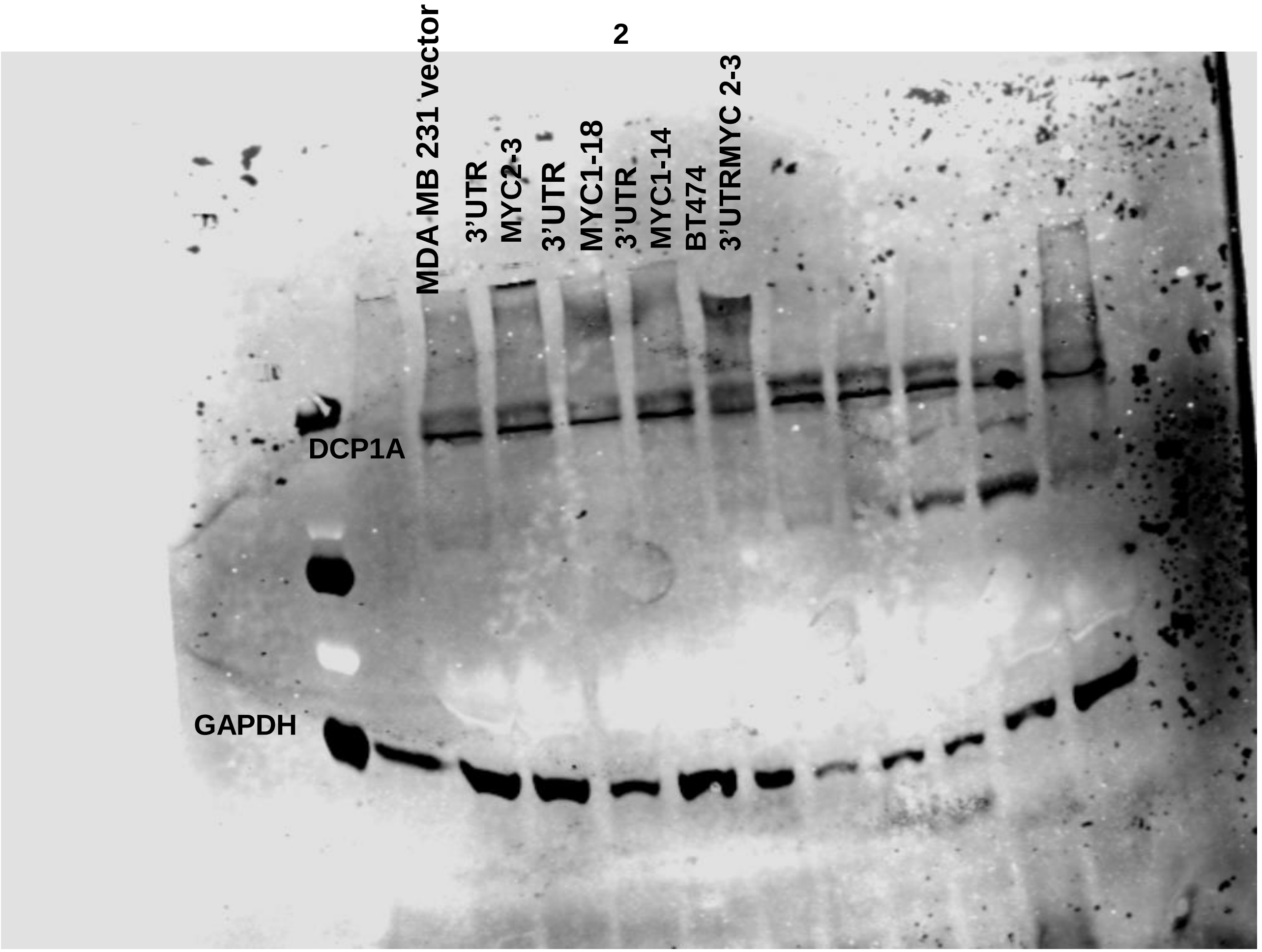

Supplementary 6B uncropped

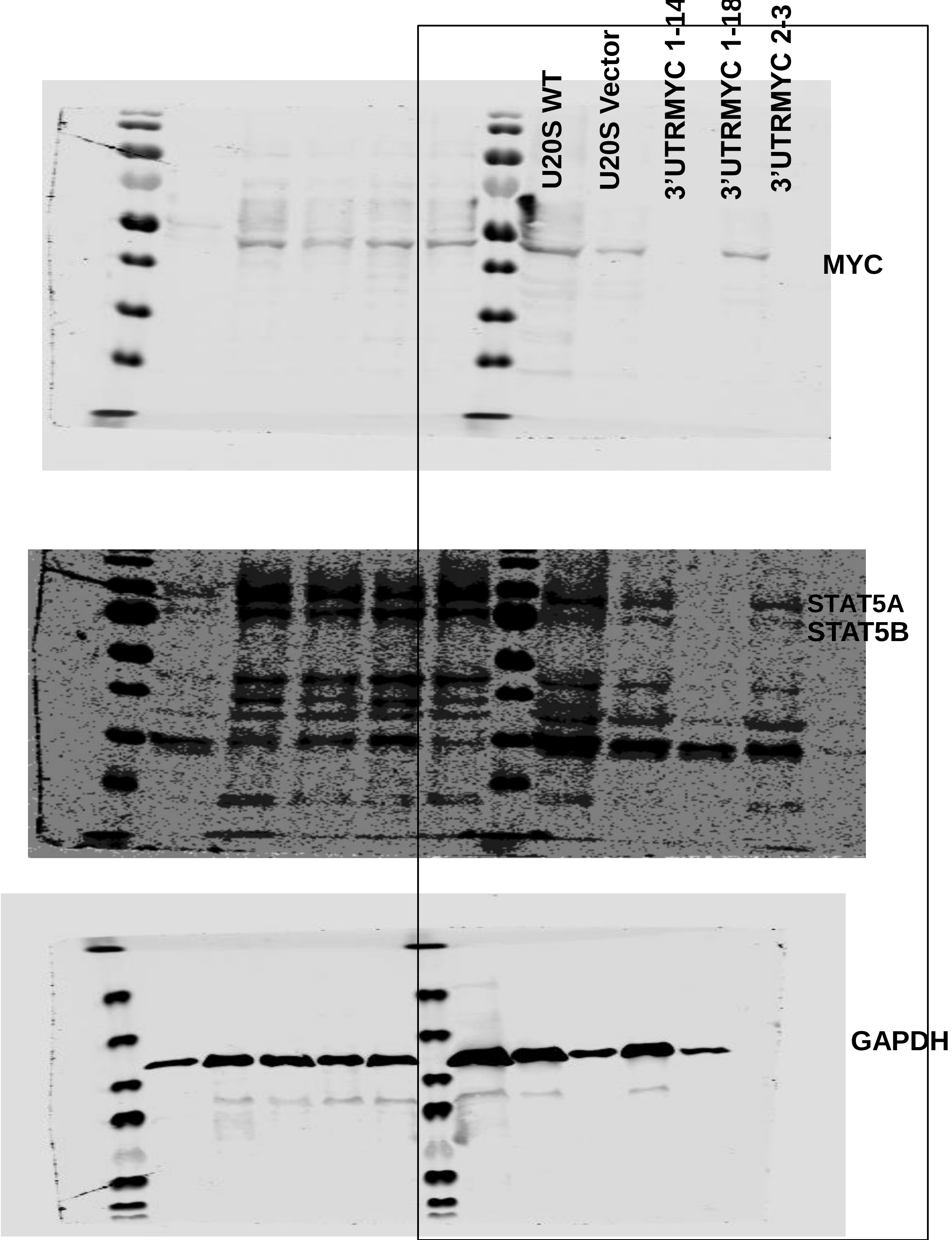

Supplementary Figure 18D E, **Uncropped**

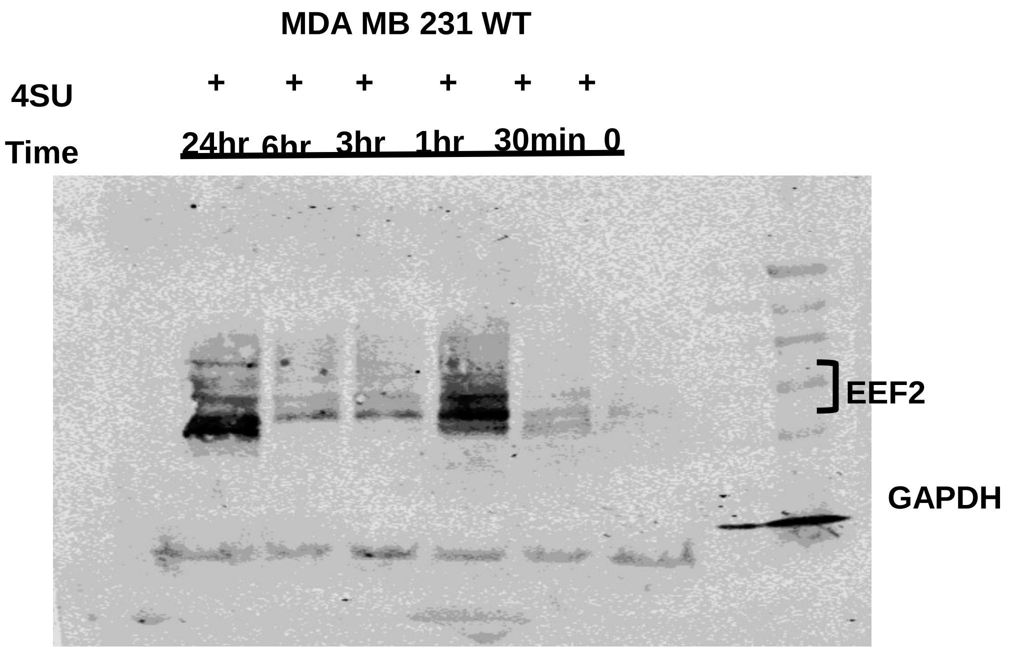

MDA MB 231 WT

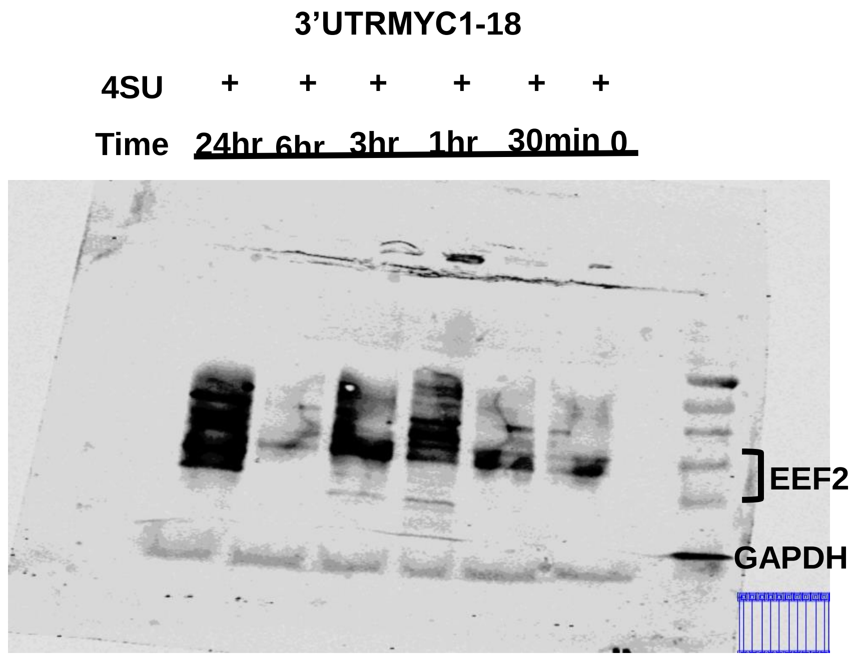

3’UTRMYC1-18
