# Supplementary material for: The Engineered Drug 3′UTRMYC1-18 Degrades the c-MYC-STAT5A/B-PD-L1 Complex In Vivo to Inhibit Metastatic Triple-Negative Breast Cancer
Source: Cancers (Basel). 2024 Jul 26;16(15):2663. doi: 10.3390/cancers16152663 (PMC11311709; doi:10.3390/cancers16152663)
Supplement: Supplementary file 1 [file cancers-16-02663-s001.zip › supplementary figures.pdf]

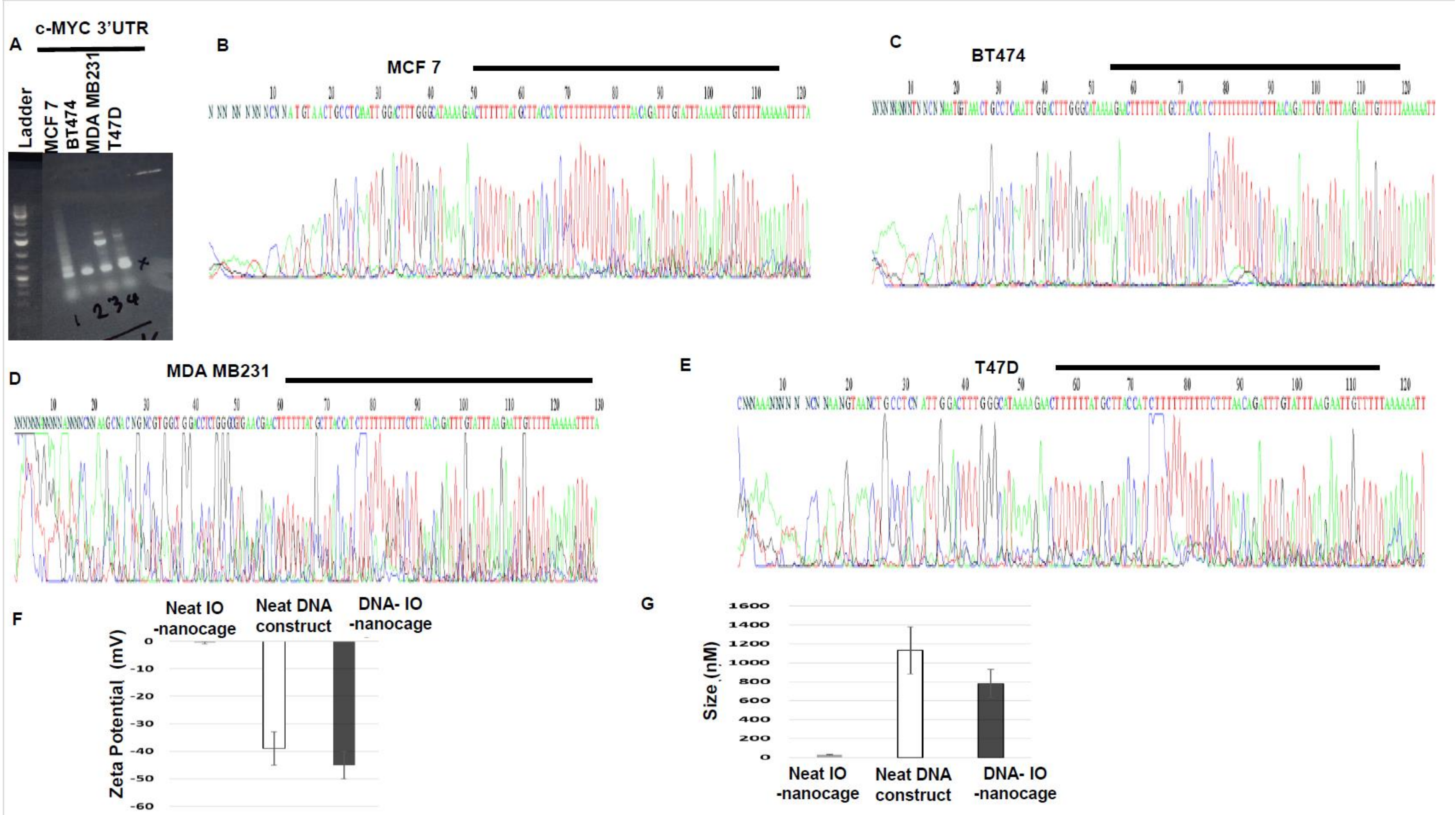

Figure S1. Identification of the stabilizing mRNA poly U sequences on the 3′UTR of c-MYC across many cancers. A. Gel image of c-MYC 3′UTR amplified from the cDNA of the MCF7, BT474, MDA MB231, and the T47D, marked in black asterisks. B. Electropherogram of MCF7 3′UTR with poly U sequences marked in black. C. Electropherogram of BT474 3′UTR with poly U sequences marked in black. D. Electropherogram of MDA

MB231 3′UTR with poly U sequences marked in black. E. Electropherogram of T47D 3′UTR with poly U sequences marked in black. F. Bar chart showing the zeta potential (mV) of the neat IO-nanocage, neat DNA constructs, and DNA-IO-nanocage. G. Bar chart showing the size (nm) of the neat IO-nanocage, neat DNA constructs, and DNA-IO-nanocages, determined by DLS.

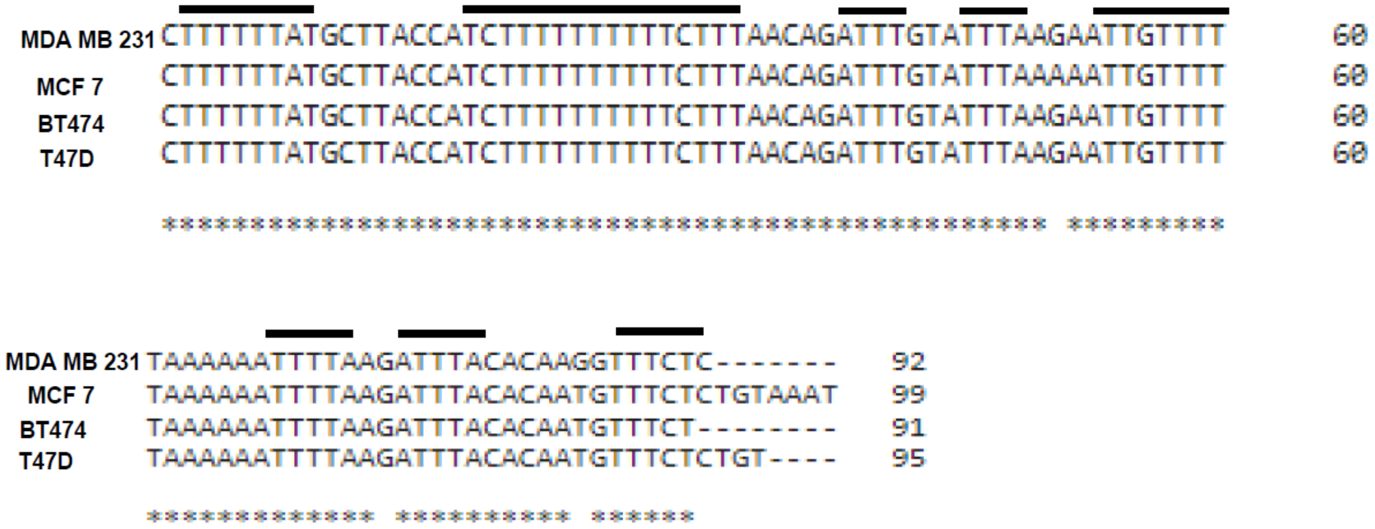

Figure S2. The stabilizing mRNA poly U sequences are 99% conserved on the 3′UTR of c-MYC across many cancers. A. Multi-alignment of poly U sequences of the MCF7, BT474, MDA MB231, and the T47D.

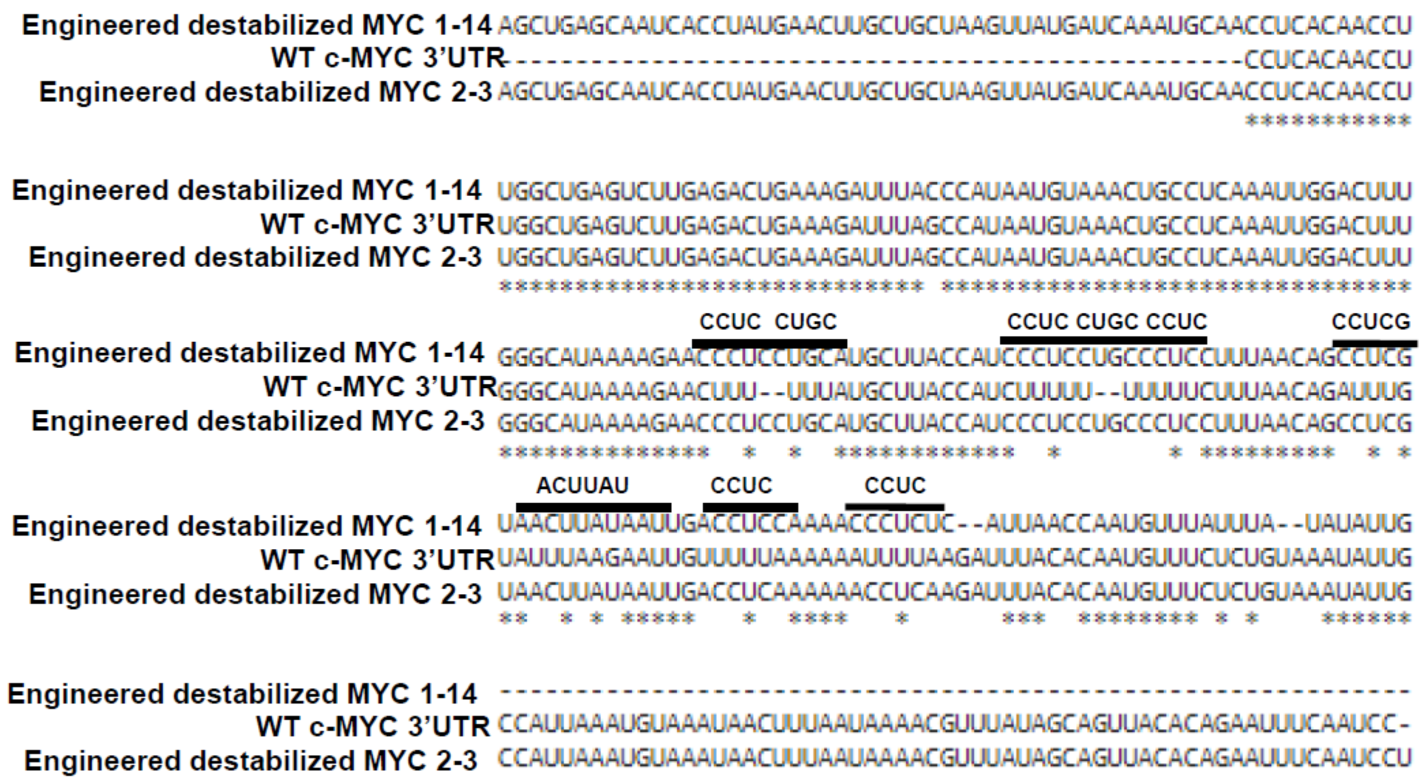

Figure S3. c-MYC 3′UTR cDNA converted to mRNA sequences with the stabilizing poly U sequences engineered to the destabilizing elements, underlined in black. A. The alignment of the 3′UTR of the WT c-MYC and the engineered destabilized c-MYC with sequences that were changed.

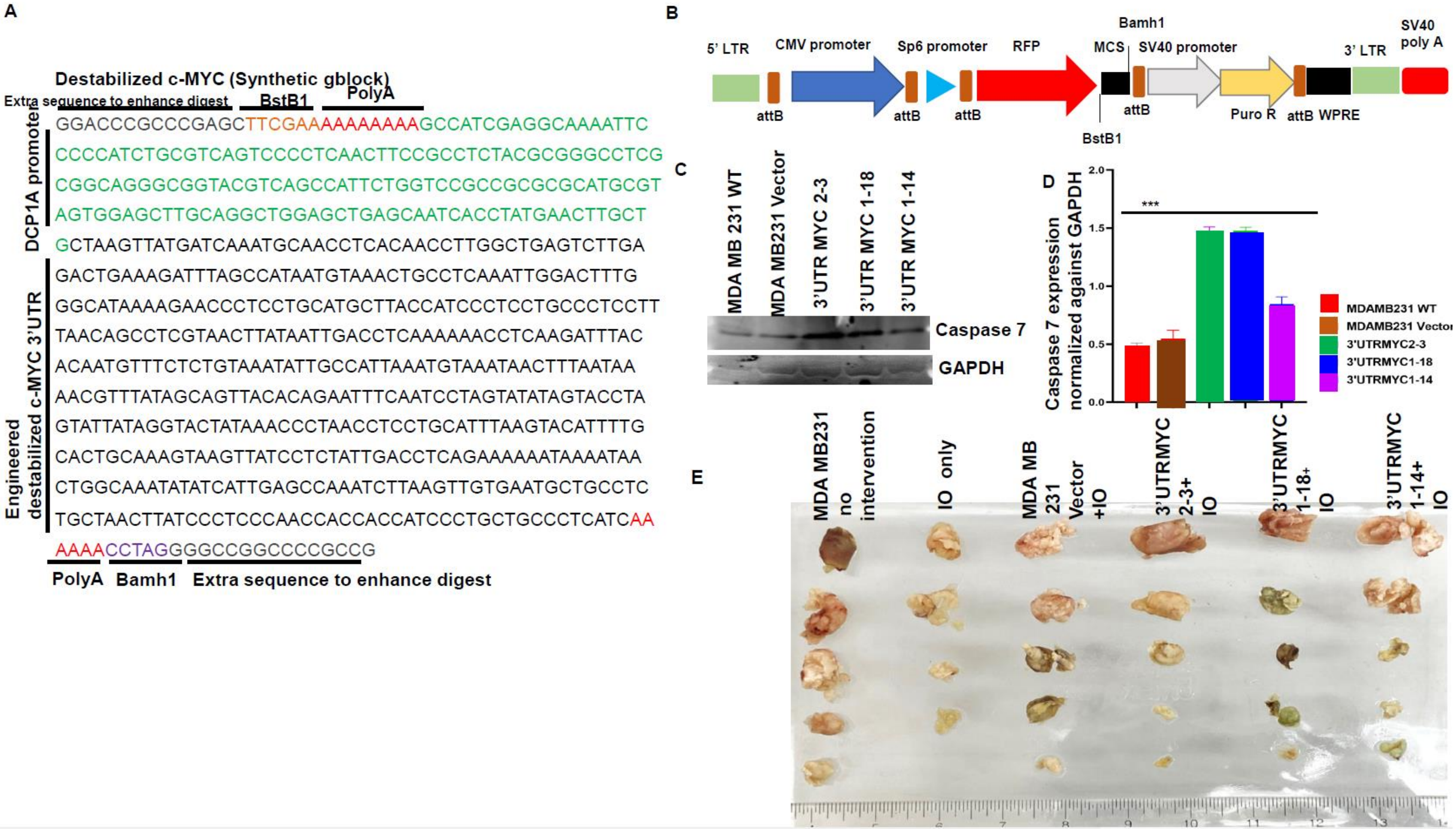

Figure S4. Design of destabilized c-MYC 3′UTR with DCP1A promoter, BstB1, BamH1 restriction sites, and plasmid vector design. A. Schematic illustration of component sequences used in the design of destabilized 3′UTR c-MYC. B. Schematic illustration of the plasmid vector components. C.

Western blot of caspase 7 and GAPDH in the controls and in cells that received the destabilized constructs. D. Bar chart shows the quantification of caspase 7 protein expression normalized against GAPDH in the treated and control groups (***$p < 0.001$, two-tailed t-test). E. Tumor sizes of the different groups: the controls and the treatment groups.

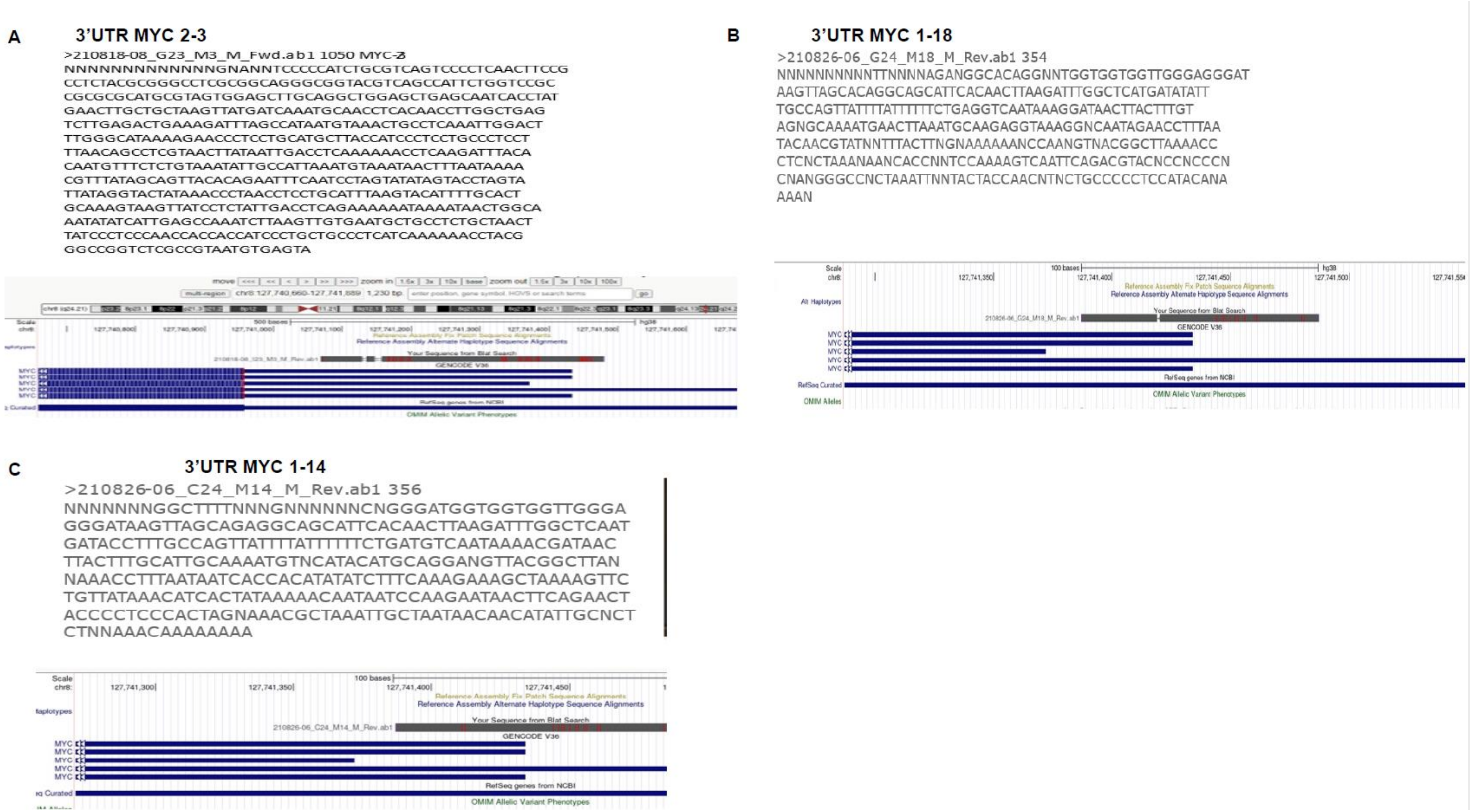

Figure S5. Sequence of cloned destabilized c -MYC constructs 3′UTRMYC2-3, 1-18, and 1-14. A. Schematic illustration of the cloned 3′UTRMYC2-3 mapped to the c-MYC 3′UTR. B. Schematic illustration of the cloned 3′UTRMYC1-18 mapped to the c-MYC 3′UTR. C. Schematic illustration of the cloned 3′UTRMYC1-14 mapped to the c-MYC 3′UTR.

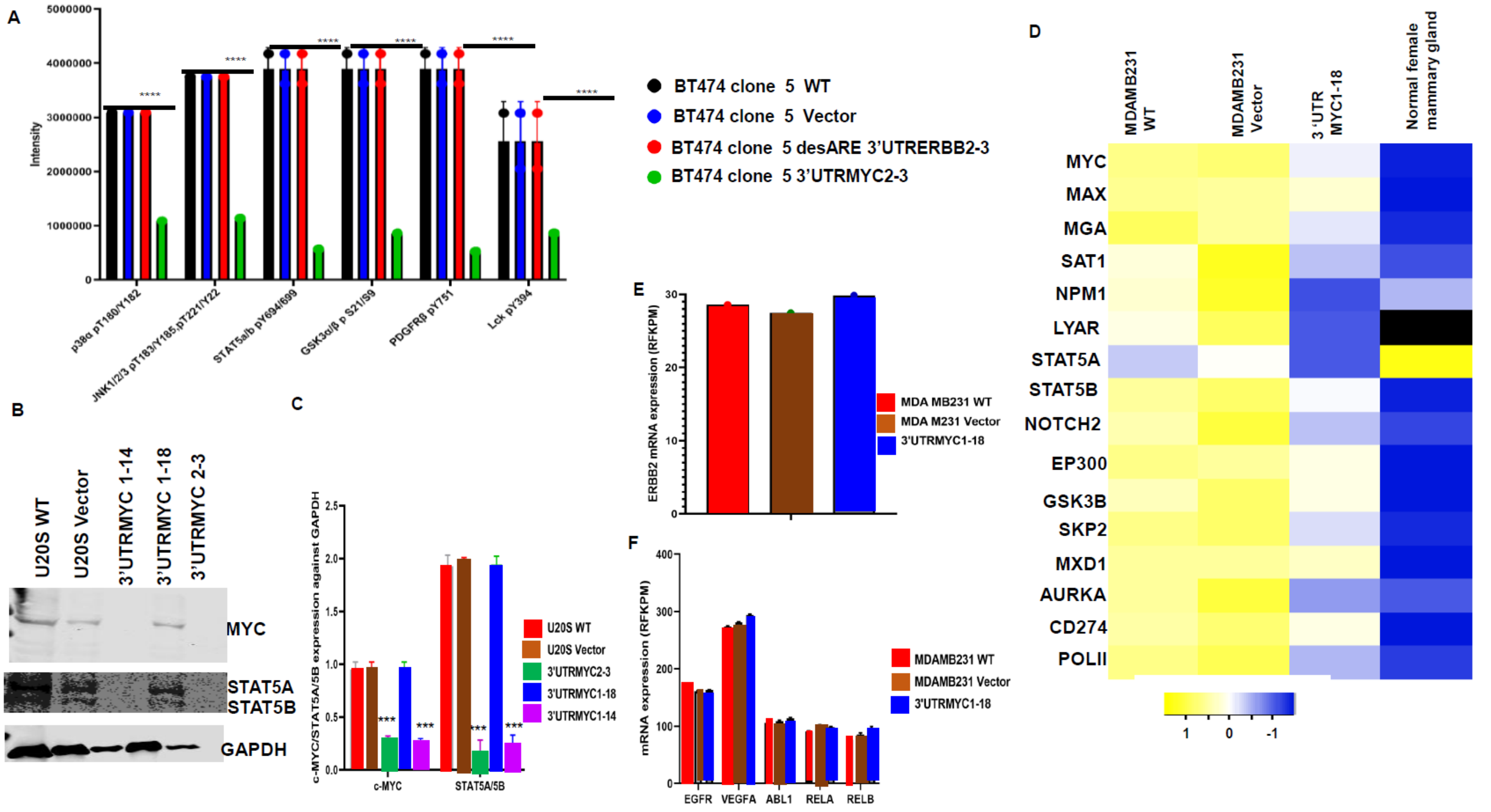

Figure S6. The destabilized ARE 3′UTR of c-MYC specifically targets MYC-dependent kinases and transcription factors in TNBC. A. Bar charts show the intensity of p38a, JNK1/2/3, STAT5A/5B, GSK3A/B, PDGFRB, and LCK in BT474 clone 5 WT (black), BT474 clone 5 vectors (blue), BT474 clone 5 desARE3′UTRERBB2-3 (red), and BT474 clone 5 3′UTRMYC2-3 (green) **** *p*-value < 0.0001, two-tailed *t*-test. B. Western blot shows the c-MYC,

STAT5A/5B, and the GAPDH protein expression in the control and the destabilized cells (N = 6). C. Quantification of the c-MYC and STAT5A/5B protein expression normalized against GAPDH (*** $p < 0.001$, two-tailed *t*-test). D. Heat map shows the expression levels of c-MYC and interaction partners in MDA MB231 WT, vector, 3′UTRMYC1-18, and normal female mammary gland gene expression from ENCODE. E. Bar charts show the ERBB2 mRNA expression in MDA MB231 WT, vector, and 3′UTRMYC1-18. F. Bar charts show the EGFR, VEGFA, ABL1, RELA, and RELB mRNA expression in MDA MB231 WT, vector, and 3′UTRMYC1-18.

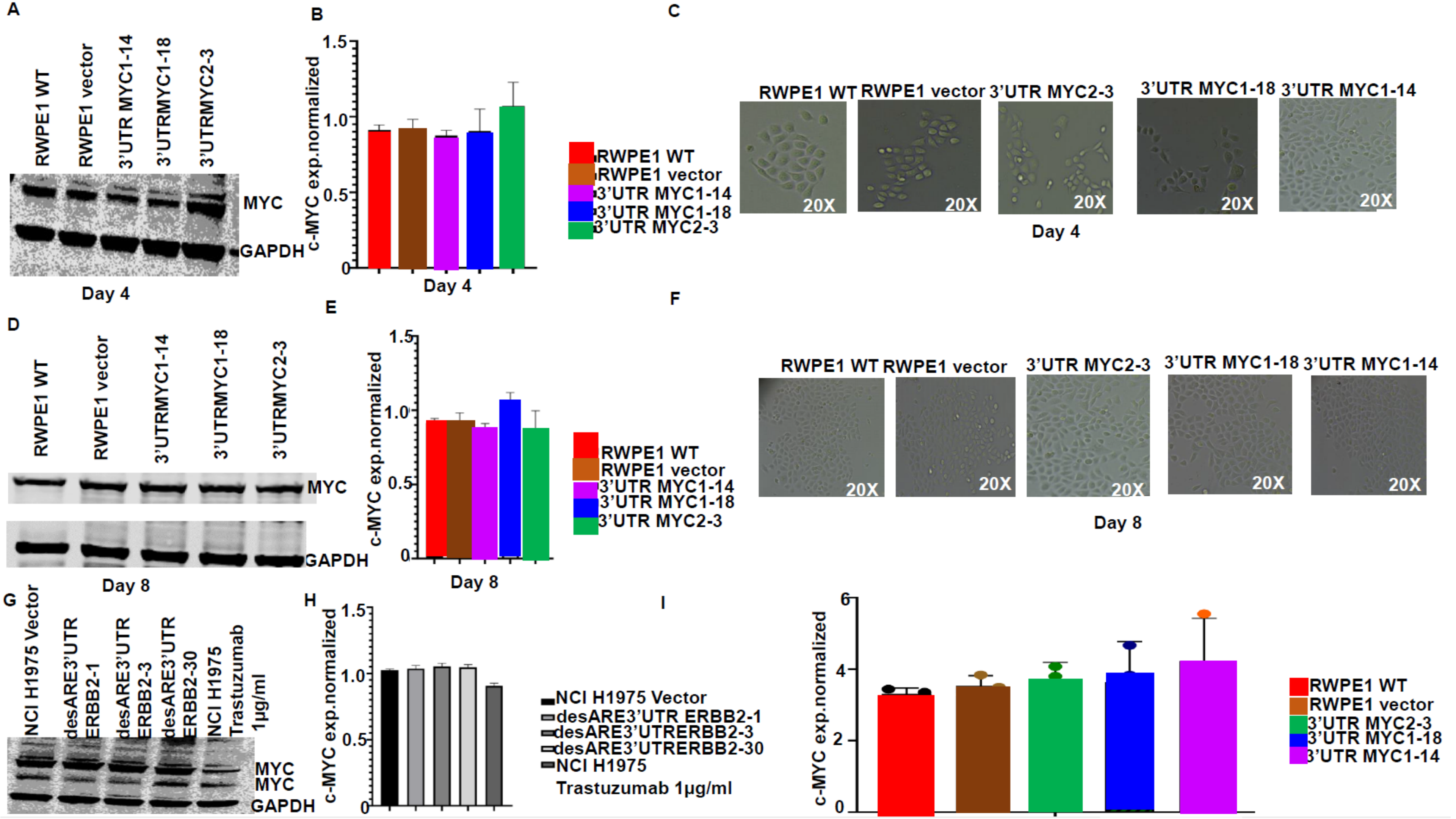

Figure S7. The engineered destabilized c-MYC constructs do not degrade c-MYC or kill normal epithelial cells. A. Western blot shows c-MYC and GAPDH protein expression in normal epithelial RWPE1 WT, vector, 3′UTRMYC1-14, 3′UTRMYC1-18, and 3′UTRMYC2-3 treated cells for 4 days. B.

Bar chart shows quantification of c-MYC normalized against GAPDH for gel image from A. C. Light microscopic image of normal epithelial cells, RWPE1 WT cells, and cells treated with vector, 3′UTRMYC1-14, 3′UTRMYC1-18, and 3′UTRMYC2-3 for 4 days (N = 4). D. Western blot shows c-MYC and GAPDH protein expression in normal epithelial cells, RWPE1 WT cells, and cells treated with vector, 3′UTRMYC1-14, 3′UTRMYC1-18, and 3′UTRMYC2-3 for 8 days. E. Bar chart shows quantification of c-MYC normalized against GAPDH for gel image from D. F. Light image of normal epithelial cells, RWPE1 WT cells, and cells treated with vector, 3′UTRMYC1-14, 3′UTRMYC1-18, and 3′UTRMYC2-3 for 8 days (N = 4). G. Western blot shows c-MYC and GAPDH protein expression in NCI H1975 vector, desARE3′UTRERBB2-1, desARE3′UTRERBB2-1, desARE3′UTRERBB2-3, desARE3′UTRERBB2-30, and NCI H1975 trastuzumab-treated cells. H. Bar chart shows quantification of c-MYC normalized against GAPDH for gel image from G. I. Bar chart shows quantification of c-MYC mRNA expression normalized against GAPDH in normal epithelial cells, RWPE1 WT cells, and cells treated with vector, 3′UTRMYC2-3, 3′UTRMYC1-18, and 3′UTRMYC1-14, for 8 days (N = 4).

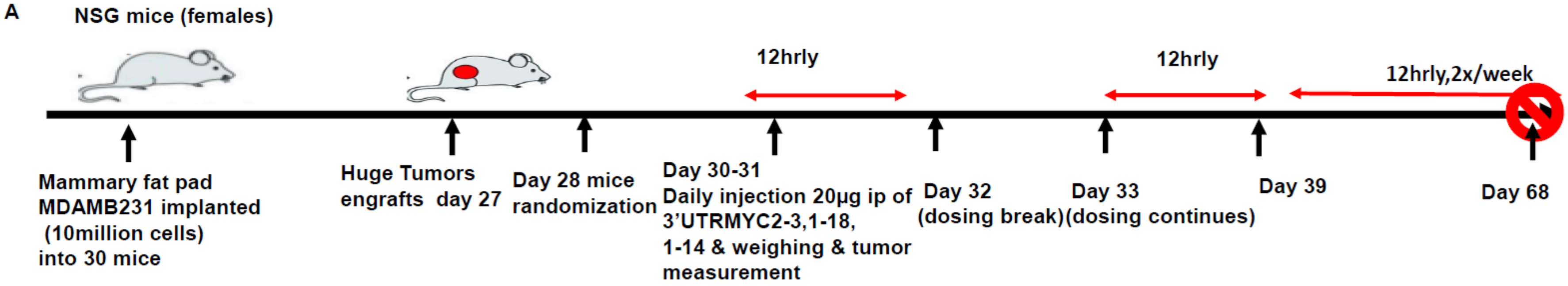

B

Days	Tumor volume (mm3) Nanocage+Vector	Tumor volume (mm3) 3'UTR MYC2-3+Nanocage	Tumor volume(mm3) 3'UTR MYC1-18+ Nanocage	Tumor volume (mm3) 3' MYC1-14+Nanocage
0	66	47	62.5	71.9
1	112.2	47	36.2125	30.9
2	107.4	30.5	49.40625	23.9
5	199.5075	148.3	178.3333	105.625
8	295.786	230.4235	103.9	175.4767
9	403.1	189.8	128.8333	122.6
12	736.1	371.4472	198	262.2344
15	836.8125	434.515	178.875	259.125
19	1290	517.0781	361.2938	328.145
21	1548.775	472	299.5	377.6
23	1413.25	527.5	867.5	1079
25	2419	864	1184	1106.5
27	2286.2	1524	1372	1284
30	2992	720	1687	1529.5
32	3177	1920	2178	1578.75
34	3965.5	2178	3249	2456.5
37	4044.5	2048	2167	2759.5

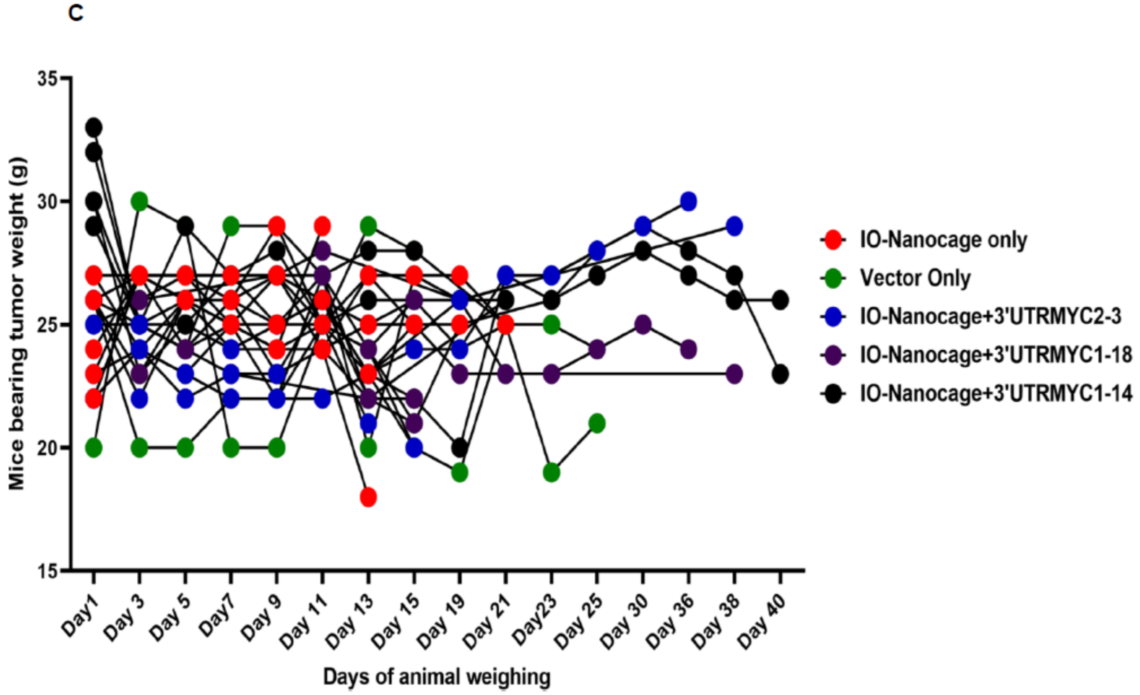

Figure S8. Schematic depiction of the animal experiment. A. Schematic illustration of the animal experiment, tumor implantation, randomization, construct administration, and daily tumor measurement and weighing. B. Chart shows the daily summation of tumor volume recording for the

control and treatment groups. C. Chart shows the monitoring of weight measurements of animals bearing tumors, both controls and the untreated group.

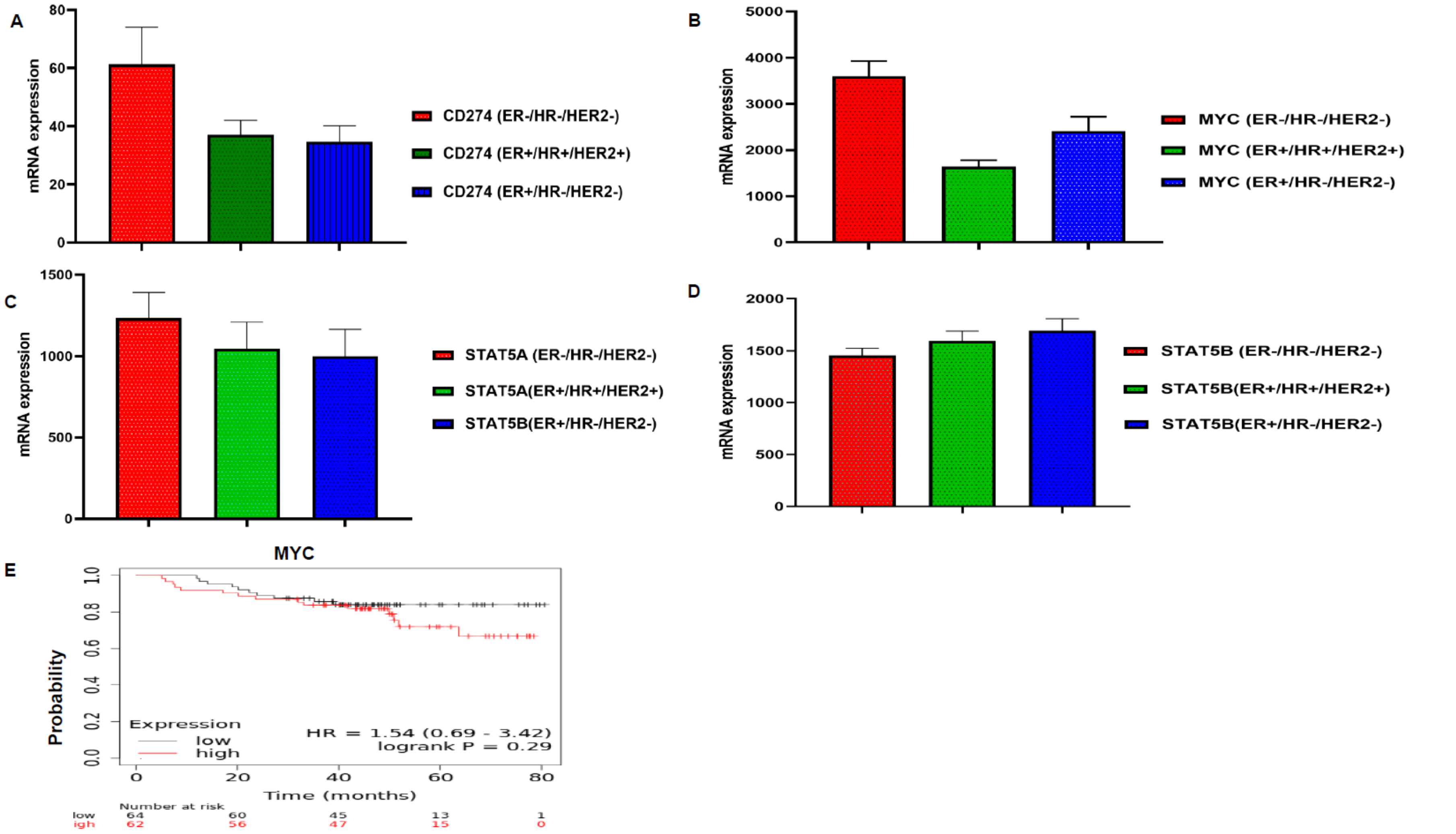

Figure S9. Gene expression profile of CD274 (PD-L1), MYC, and STAT5A/5B in (ER−/HR−/HER2−), (ER+/HR+/HER2+), and (ER+/HR-/HER2-). A. Bar chart shows the CD274 (PD-L1) expression in different subtypes of breast cancer (ER-/HR-/HER2-), (ER+/HR+/HER2+), and (ER+/HR-/HER2-) .B. Bar chart shows the c-MYC expression in different subtypes of breast cancer (ER-/HR-/HER2-), (ER+/HR+/HER2+), and (ER+/HR-/HER2-). C. Bar chart shows the STAT5A expression in different subtypes of breast cancer (ER-/HR-/HER2-), (ER+/HR+/HER2+), and (ER+/HR-/HER2-). D. Bar chart shows the STAT5B expression in different subtypes of breast cancer (ER-/HR-/HER2-), (ER+/HR+/HER2+), and (ER+/HR-/HER2-). E. Kaplan–Meier survival curve for c-MYC (low) and c-MYC (high) expression in TNBC.

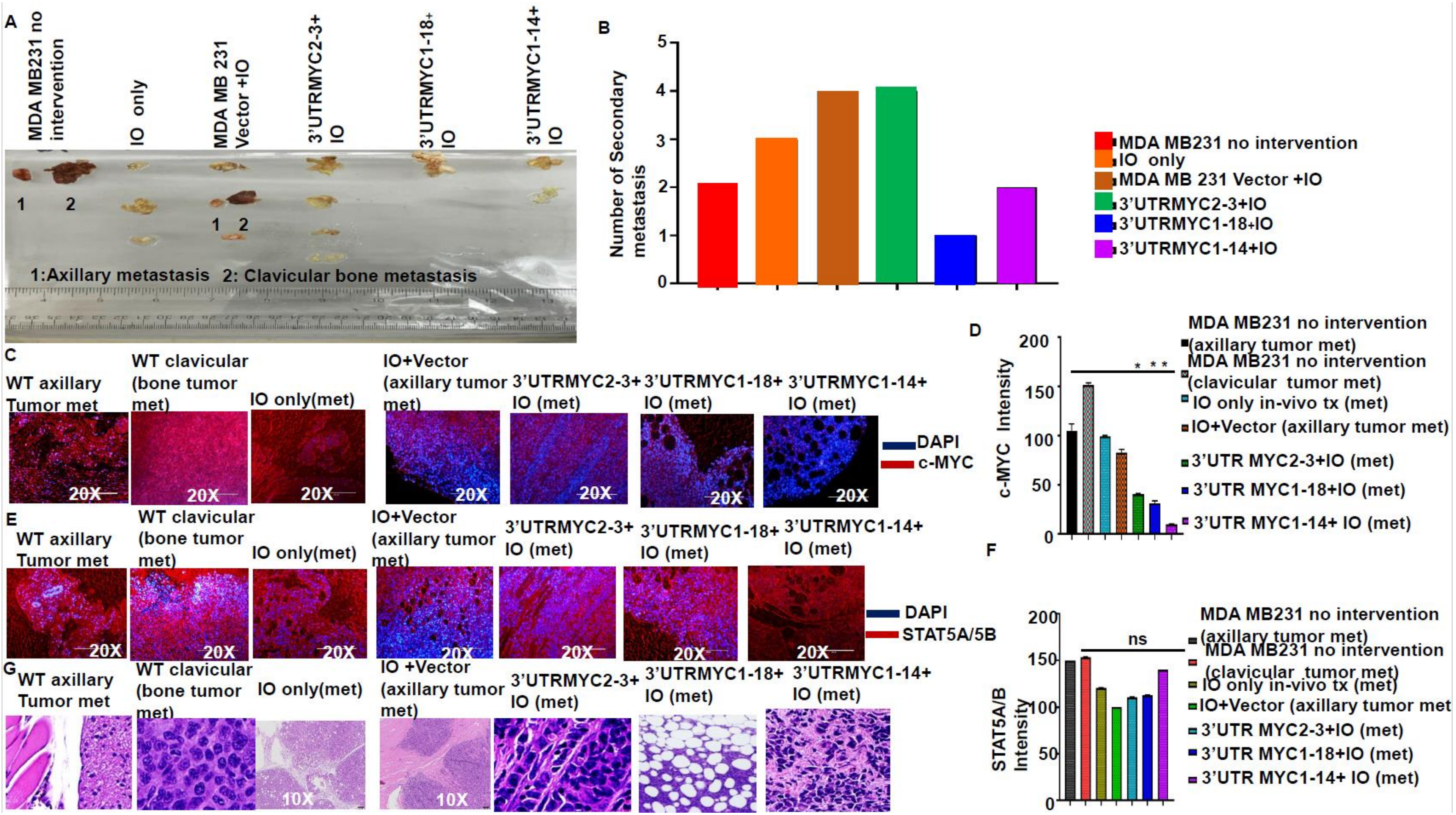

Figure S10. IO-nanocages delivered constructs and inhibited metastatic TNBC by downregulating c-MYC; however, the unresponsive tumors were driven by c-MYC-independent STAT5A/5B expression. A. Metastatic tumor images; sizes from the different controls and the treatment groups (N = 2 WT, N = 3 nanocage only, N = 4 IO-nanocage plus vector, N = 4 3'UTRMYC2-3, N = 1 3'UTRMYC1-18, N = 2 3'UTRMYC1-18). B. Quantification of

metastatic tumors from the controls and the treated groups. C. Immunofluorescence images of metastatic tumors from the controls and the treatment groups stained with c-MYC (red), DAPI nuclei (blue), groups (N = 2 WT, N = 3 nanocage only, N = 4 IO-nanocage plus vector, N = 4 3′UTRMYC2-3, N = 1 3′UTRMYC1-18, N = 2 3′UTRMYC1-18). D. Quantification of c-MYC in metastatic tumors from the controls and the treated groups by immunofluorescence. * $p < 0.044$ (WT vs. 3′UTRMYC2-3), * $p < 0.03$ (WT vs. 3′UTRMYC1-18 and 1-14). E. Immunofluorescence images of metastatic tumors from the control and the treatment groups stained with STAT5A/5B (red), DAPI nuclei (blue), groups (N = 2 WT, N = 3 IO-nanocage only, N = 4 nanocage plus vector, N = 4 3′UTRMYC2-3, N = 1 3′UTRMYC1-18, N = 2 3′UTRMYC1-18). F. Quantification of STAT5A/5B in metastatic tumors from the controls and treated groups by immunofluorescence. p = ns.

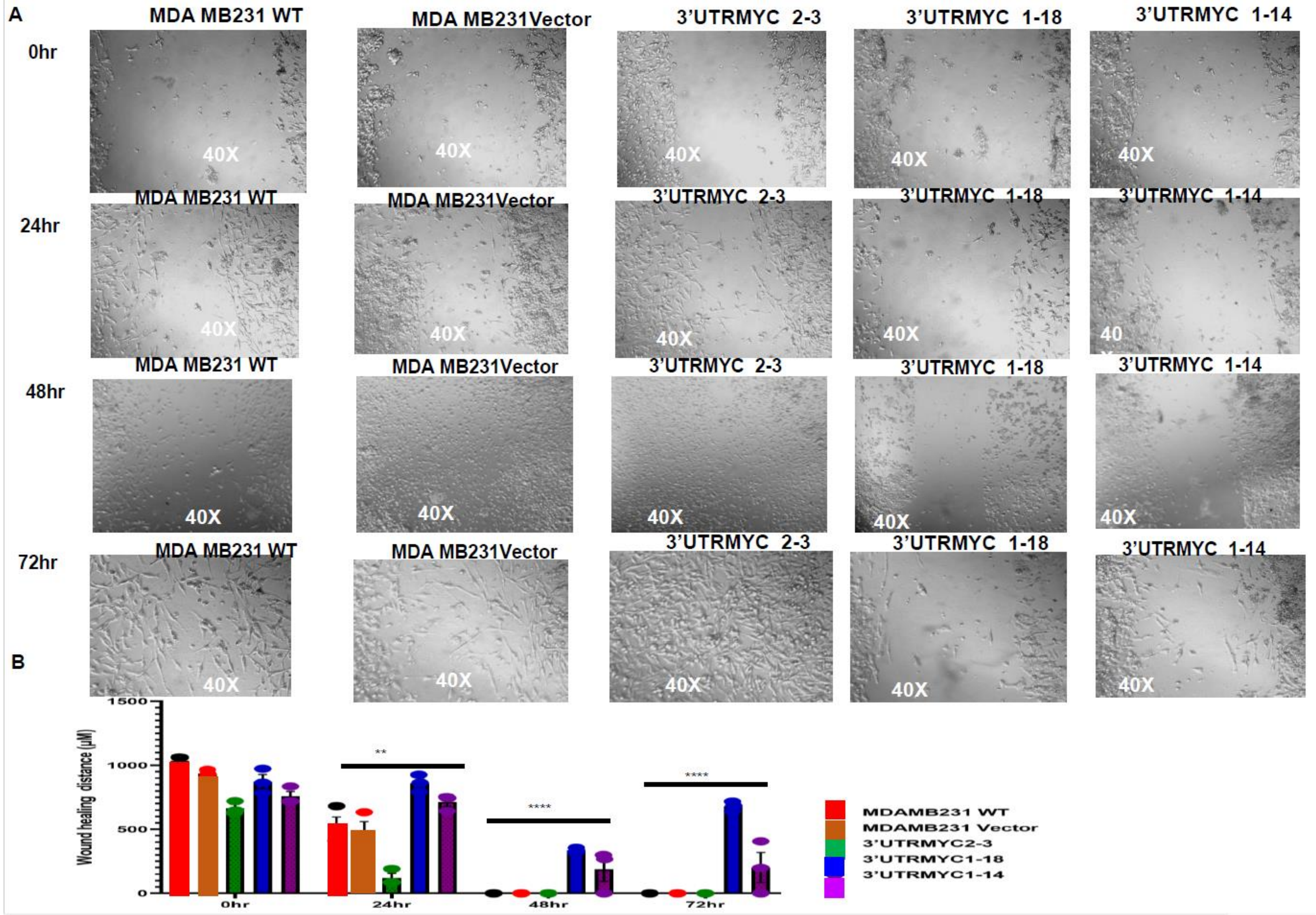
A
MDA MB231 WT
MDA MB231Vector
3'UTRMYC 2-3
3'UTRMYC 1-18
3'UTRMYC 1-14
0hr
24hr
48hr
72hr
40X
B
Wound healing distance (μM)
1500
1000
500
0
0hr
24hr
48hr
72hr
**
****
****
MDAMB231 WT
MDAMB231 Vector
3'UTRMYC2-3
3'UTRMYC1-18
3'UTRMYC1-14

Figure S11. Migration assay of the treated and untreated MDA MB231. A. Microscopic images of migration of the cells from the MDA MB231WT, vector, 3′UTRMYC2-3, 1-18, and 1-14 at 0 h, 24 h, 48 h, and 72 h. B. Bar charts show the quantification of wound healing in the control and treated groups (** $p < 0.01$, **** $p < 0.0001$, two-tailed *t*-test).

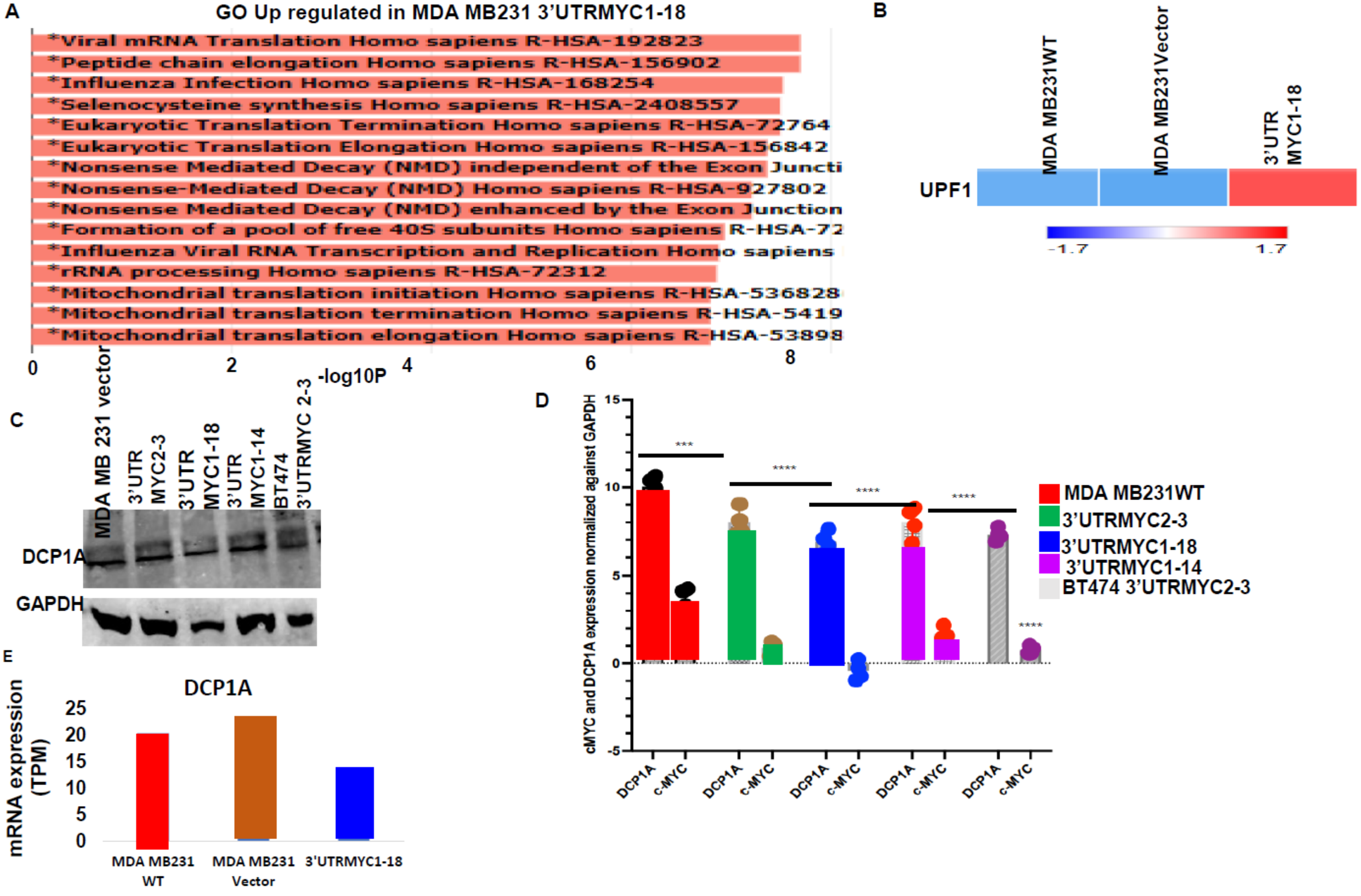
A
GO Up regulated in MDA MB231 3'UTRMYC1-18
*Viral mRNA Translation Homo sapiens R-HSA-192823
*Peptide chain elongation Homo sapiens R-HSA-156902
*Influenza Infection Homo sapiens R-HSA-168254
*Selenocysteine synthesis Homo sapiens R-HSA-2408557
*Eukaryotic Translation Termination Homo sapiens R-HSA-72764
*Eukaryotic Translation Elongation Homo sapiens R-HSA-156842
*Nonsense Mediated Decay (NMD) independent of the Exon Juncti
*Nonsense-Mediated Decay (NMD) Homo sapiens R-HSA-927802
*Nonsense Mediated Decay (NMD) enhanced by the Exon Junction
*Formation of a pool of free 40S subunits Homo sapiens R-HSA-72
*Influenza Viral RNA Transcription and Replication Homo sapiens
*rRNA processing Homo sapiens R-HSA-72312
*Mitochondrial translation initiation Homo sapiens R-HSA-536828
*Mitochondrial translation termination Homo sapiens R-HSA-5419
*Mitochondrial translation elongation Homo sapiens R-HSA-53898
0
2
4
6
8
-log10P
B
MDA MB231WT
MDA MB231Vector
3'UTR MYC1-18
UPF1
-1.7
1.7
C
MDA MB 231 vector
3'UTR MYC2-3
3'UTR MYC1-18
3'UTR MYC1-14
BT474 3'UTRMYC 2-3
DCP1A
GAPDH
D
cMYC and DCP1A expression normalized against GAPDH
15
10
5
0
-5
***
****
****
****
****
DCP1A
c-MYC
MDA MB231WT
3'UTRMYC2-3
3'UTRMYC1-18
3'UTRMYC1-14
BT474 3'UTRMYC2-3
E
DCP1A
mRNA expression (TPM)
25
20
15
10
5
0
MDA MB231 WT
MDA MB231 Vector
3'UTRMYC1-18

Figure S12. The engineered destabilized 3′UTR of c-MYC is sequence-specific and works through nonsense-mediated decay as designed. A. Upregulated GO terms in the destabilized cells carrying 3′UTRMYC1-18. B. Heat map of UPF1 mRNA expression in the WT, vector-, and 3′UTRMYC1-18-treated-cells. C. Western blot shows DCP1A and the GAPDH in the control and treated cells. D. Bar charts show the head-to-head comparison of DCP1A and c-MYC protein expression normalized against GAPDH in the same cells, both treated and untreated (*** $p < 0.001$, **** $p < 0.0001$, two-tailed t-test). E. DCP1A mRNA expression from the controls and the 3′UTRMYC1-18-treated cells.

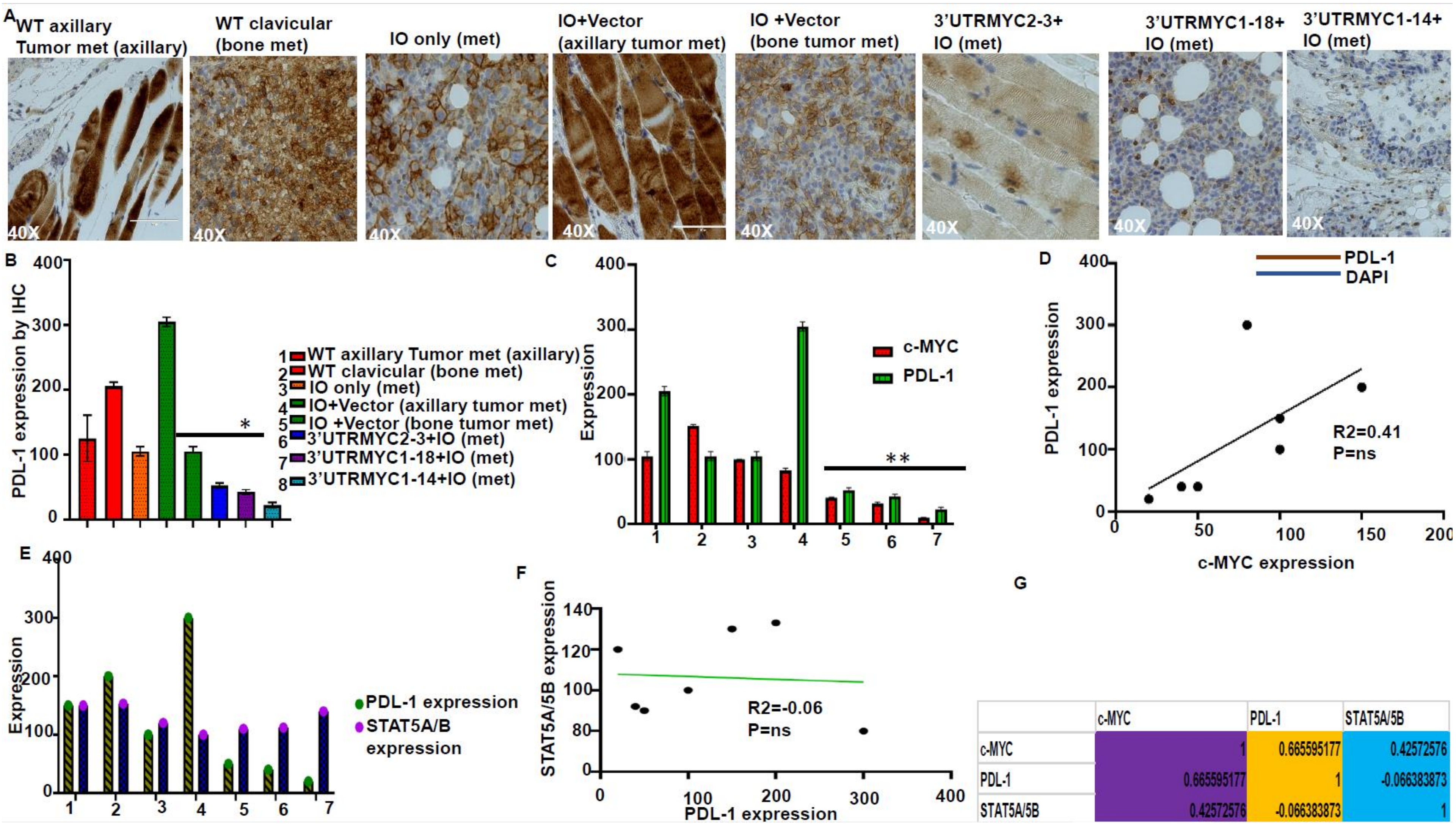

Figure S13. The nonresponsive metastatic TNBC tumors are driven by STAT5A/5B signal independently of c-MYC. A. Immunohistochemistry staining of PD-L1 in metastatic tumors from the control and treated groups; PD-L1 (brown), DAPI nuclei (blue). (N = 2 WT, N = 3 nanocage only, N

= 4 IO-nanocage plus vector, N = 4 3′UTRMYC2-3, N = 1 3′UTRMYC1-18, N = 2 3′UTRMYC1-14). B. Quantification of PD-L1 in metastatic tumors from the control and treated groups,* $p < 0.01$ (WT vs 3′UTRMYC2-3 + nanocage, 1-18 and 1-14). C. Head-to-head quantification of PD-L1 and c-MYC in the same metastatic tumors from the control and treated groups. ** $p < 0.001$ (WT vs. 3′UTRMYC2-3 + nanocage, 1-18 and 1-14). D. Graph shows the correlation analysis between PD-L1 and c-MYC in the metastatic tumors, $R^2 = 0.42$, p = ns. E. Head-to-head quantification of PD-L1 and STAT5A/5B in metastatic tumors from the control and treated groups, p = ns for STAT5A/5B (WT, nanocage + vector vs. 3′UTRMYC2-3+nanocage, 1-18 and 1-14). F. Graph shows the correlation analysis between PD-L1 and STAT5A/5B in the metastatic tumors, $R^2 = -0.06$, p = ns. G. Table shows the multiple correlation analysis between c-MYC, STAT5A, and 5B -PD-L1 in the metastatic tumors from the control and treated groups.

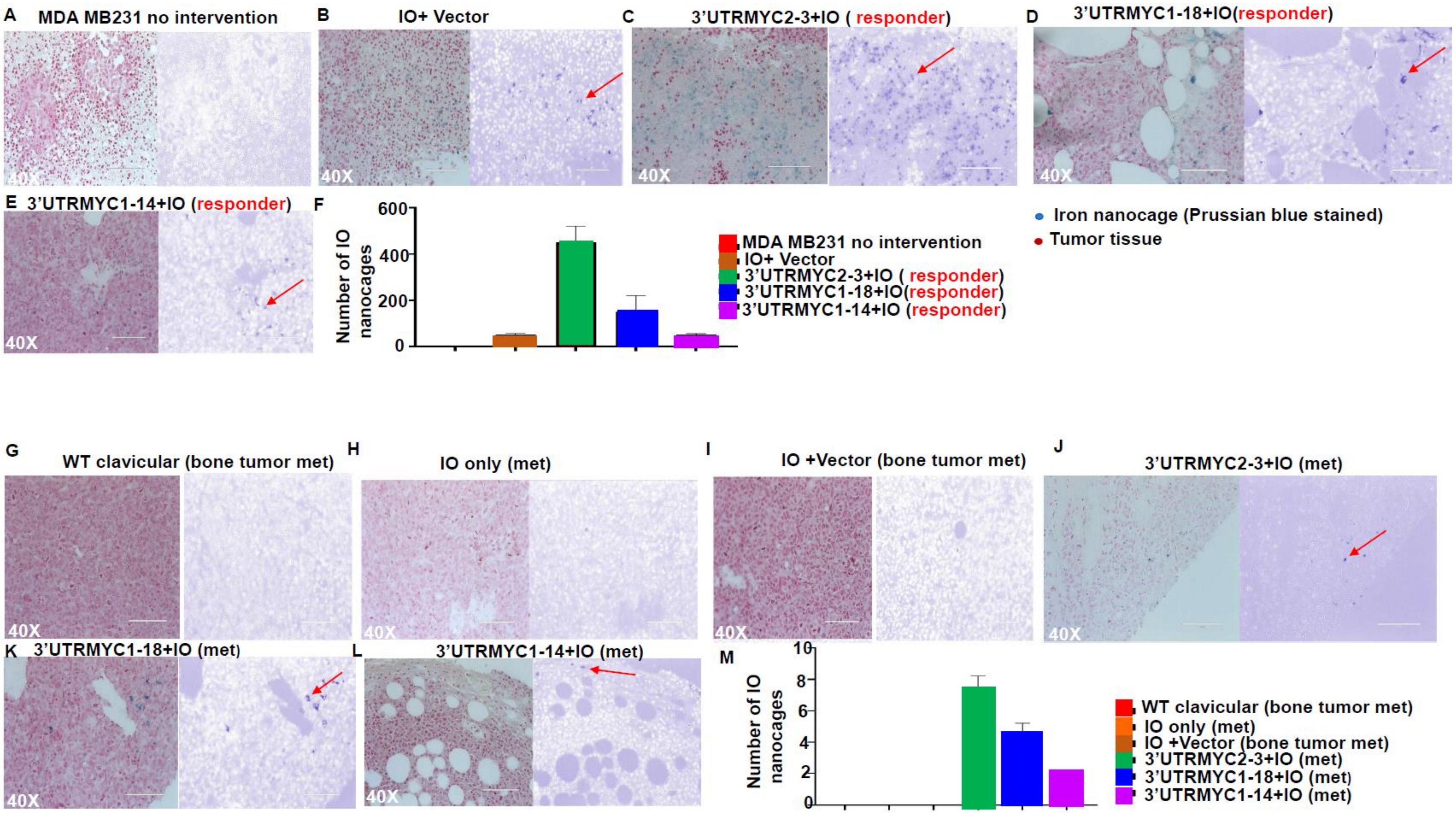

Figure S14. Nanocage detection in primary and metastatic treated tumors. A. WT no-intervention primary tumor group, no IO-nanocage detected (N = 5). B. IO-nanocage + vector-treated primary tumor group, IO-nanocage detected (red arrow) (N = 2). C. 3′UTRMYC2-3-treated primary tumor

group, IO-nanocage detected (red arrow) (N = 4). D. 3′UTRMYC1-18-treated primary tumor group, IO-nanocage detected (red arrow) (N = 2). E. 3′UTRMYC1-14-treated primary tumor group, IO-nanocage detected (red arrow) (N = 2). F. Quantification of IO-nanocages detected in the primary tumor. G. WT clavicular bone metastatic tumor group, no IO-nanocage detected (N = 2). H. Nanocage-only metastatic tumor group, no IO-nanocage detected (N = 3). I. Nanocage + vector metastatic tumor group, no IO-nanocage detected (N = 4). J. 3′UTRMYC2-3-treated metastatic tumor group, IO-nanocage detected (red arrow) (N = 4). K. 3′UTRMYC1-18-treated metastatic tumor group, IO-nanocage detected (red arrow) (N = 1). L. 3′UTRMYC1-14-treated metastatic tumor group, IO-nanocage detected (red arrow) (N = 2). M. Quantification of nanocages detected in the metastatic tumor.

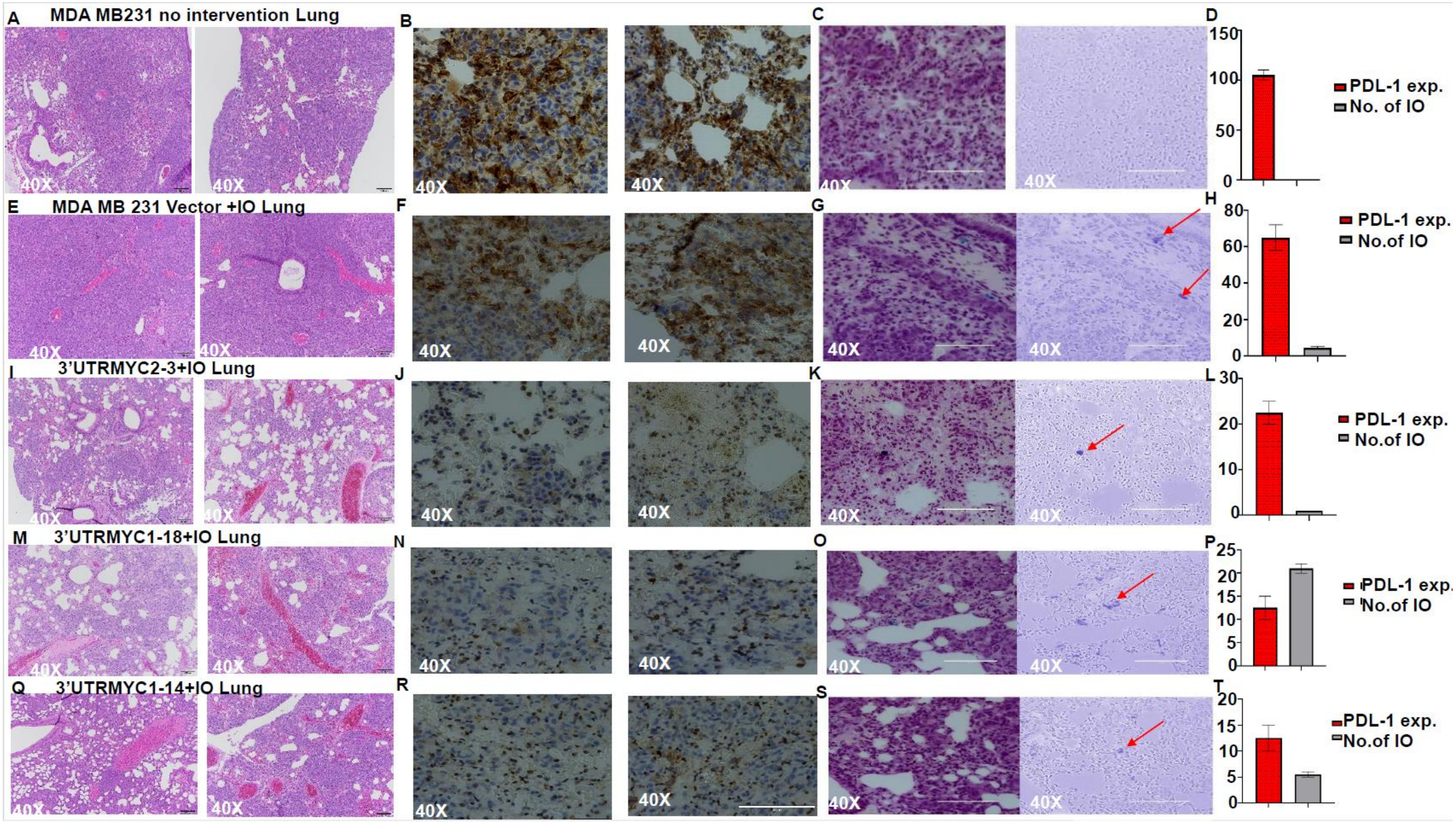

Figure S15. IO- nanocage package delivered destabilized c-MYC constructs to the lungs, targeting c-MYC-STAT5A/5B-PD-L1 in the lungs, and inhibited distant organ lung metastasis. A. H&E stain of the lung metastatic tumor from the MDA MB231 no-intervention group (N = 3). B. PD-L1

stain of lung metastatic tumor from the MDA MB231 no-intervention group (N = 3). C. Prussian blue stain of lung metastasis tumor from the MDA MB231 no-intervention group (N = 3). D. The quantification of PD-L1 and nanocages, no IO-nanocage detected in the MDA MB231 no-intervention group. E. H&E stain of the lung metastatic tumor from MDA MB231 of the vector plus IO-nanocage-only intervention group (N = 2). F. PD-L1 stain of lung metastatic tumor of IO-nanocage plus vector intervention group (N = 2). G. Prussian blue stain of lung metastatic tumor of IO-nanocage plus vector intervention group, IO-nanocages marked in red arrow (N = 2). H. Quantification of PD-L1 and nanocages. I. H&E stain of the lung metastatic tumor from the MDA MB231 of 3′UTRMYC2-3 intervention group (N = 4). J. PD-L1 stain of the lung metastatic tumor from MDA MB231 of 3′UTRMYC2-3 intervention group (N = 4). K. Prussian blue stain of lung metastatic tumor in the 3′UTRMYC2-3 intervention group, IO-nanocages marked in red arrow (N = 4). L. Quantification of PD-L1 and nanocages. M. H&E stain of the lung metastatic tumor from the MDA MB231 of 3′UTRMYC1-18 intervention group (N = 4). N. PD-L1 stain of the lung metastatic tumor stain from the MDA MB231 of 3′UTRMYC1-18 intervention group (N = 4). O. Prussian blue stain of the lung metastatic tumor in the 3′UTRMYC1-18 intervention group, IO-nanocages marked in red arrow (N = 4). P. Quantification of PD-L1 and nanocages. Q. H&E stain from the lung metastatic tumor from the MDA MB231 of 3′UTRMYC1-14 intervention group (N = 4). R. PD-L1 stain of the lung metastatic tumor stain from the MDA MB231 of 3′UTRMYC1-14 intervention group (N = 4). S. Prussian blue stain of lung metastasis tumor in the 3′UTRMYC1-14 intervention group, IO-nanocages marked in red arrow (N = 4). T. Quantification of PD-L1 and nanocages.

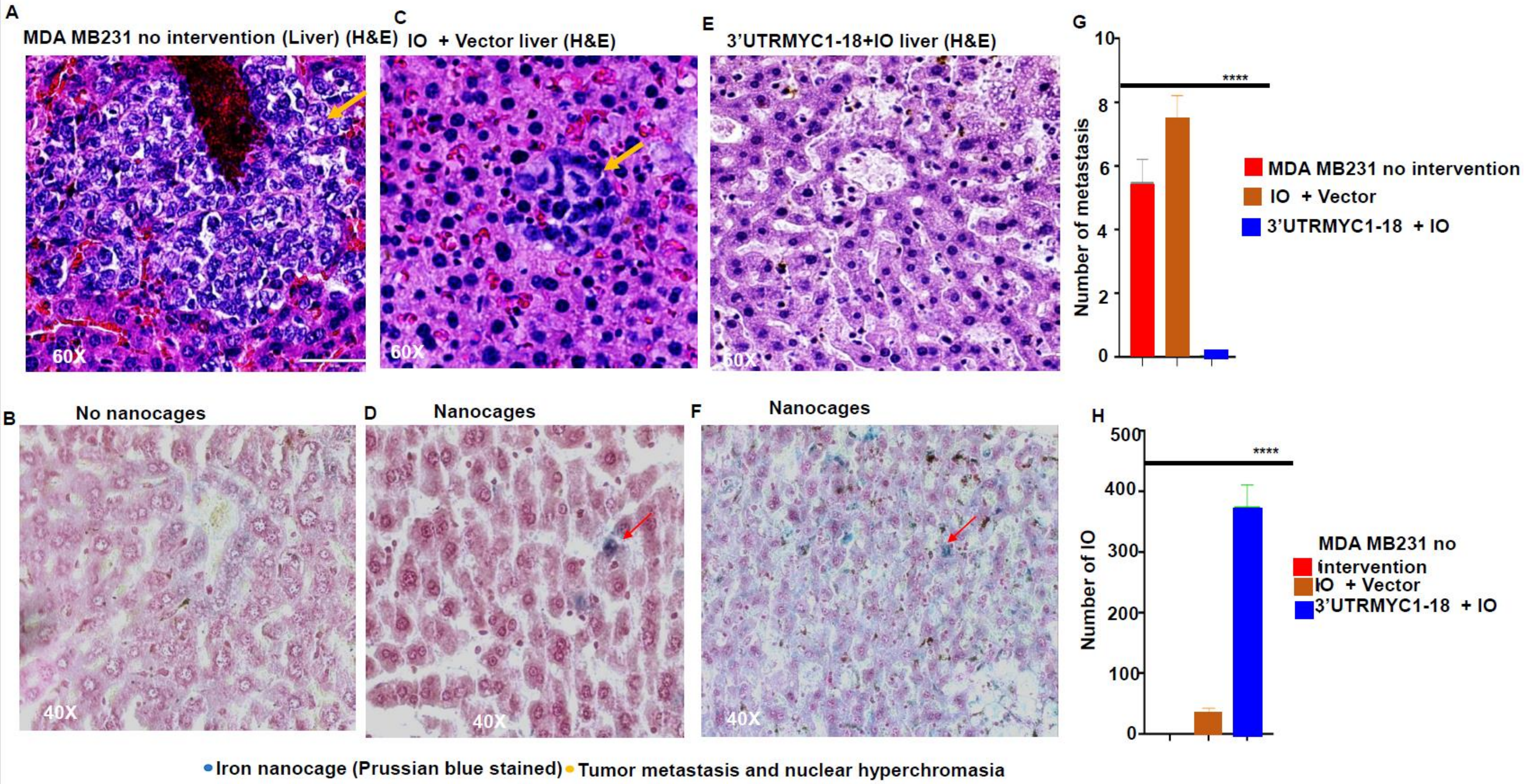

Figure S16. IO-nanocage robustly delivered destabilized c-MYC constructs to the liver and inhibited liver metastasis. A. H&E stain of the MDA MB231 no-intervention liver showing hyperchromasis and metastasis (N = 4). B. Prussian blue stain of IO-nanocages in MDA MB231 no-intervention group, no IO-nanocage found (N = 4). C. H&E stain of the MDA MB231 liver treated with IO-nanocage plus vector showing hyperchromasis and metastasis (N = 4). D. Prussian blue stain of IO-nanocages in IO-nanocage plus vector-treated group, IO-nanocage found, marked with red arrow (N

= 4). E. H&E stain of the MDA MB231 3′UTRMYC1-18-treated liver showing no hyperchromasis and no metastasis (N = 4). F. Prussian blue stain of IO-nanocages in 3′UTRMYC1-18 plus IO-nanocages-treated group, IO-nanocages were robustly found, marked with red arrow (N = 4).

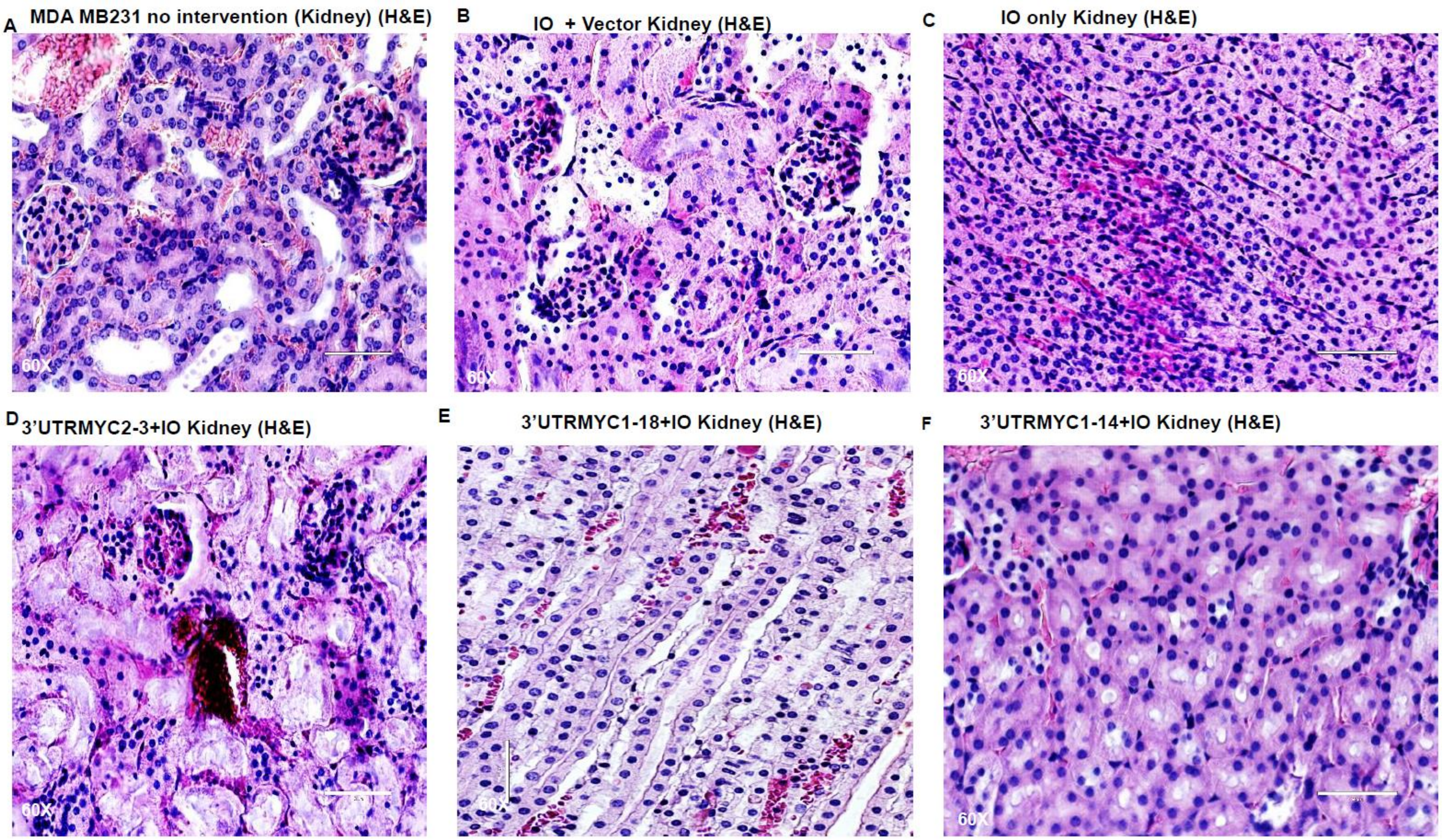

A MDA MB231 no intervention (Kidney) (H&E)
B IO + Vector Kidney (H&E)
C IO only Kidney (H&E)
D 3'UTRMYC2-3+IO Kidney (H&E)
E 3'UTRMYC1-18+IO Kidney (H&E)
F 3'UTRMYC1-14+IO Kidney (H&E)
60X
60X
60X
60X
60X
60X

Figure S17. H&E stain of the kidney tissues. A. H&E stain of the MDA MB231 kidney no-treatment group (N = 4). B. H&E stain of the MDA MB231 IO-nanocage plus the vector-treated group (N = 4). C. H&E stain of the MDA MB231 IO-nanocage-only treated group (N = 4). D. H&E stain of the MDA MB231 IO-nanocage plus 3′UTRMYC2-3-treated group (N = 4). E. H&E stain of the MDA MB231 IO-nanocage plus 3′UTRMYC1-18-treated group; tissue has normal renal tubular cellular architecture (N = 4). F. H&E stain of the MDA MB231 IO-nanocage plus 3′UTRMYC1-14-treated group (N = 4).

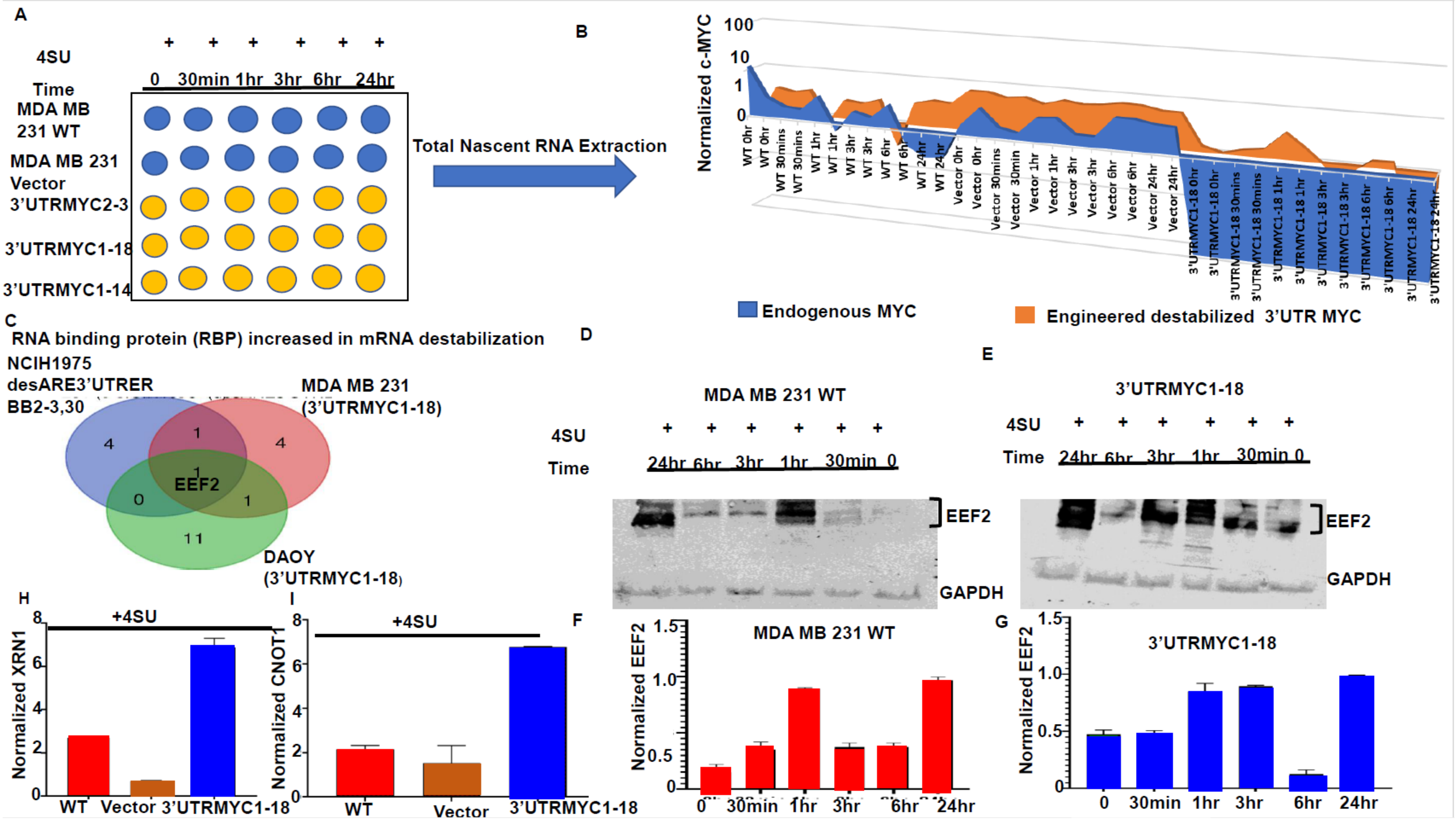

Figure S18. The engineered destabilized 3′UTR overwrote endogenous MYC by upregulation of EEF2, XRN1, and CNOT1. A. Schematic depiction of 4SU pulse-chase nascent mRNA labeling of the control cell and the cells carrying the destabilized constructs. B. Graph shows time-dependent

mRNA expression of the endogenous (blue) and destabilized 3′UTRMYC (orange) in the same cells from 0hr–24hrs in the controls and destabilized cells. C. Venn diagrams show EEF2 as intercept between NCI H1975 desARE3′UTRERBB2-3, 30, MDA MB231 3′UTRMYC1-18, and DAOY 3′UTRMYC1-18. D. Western blot of EEF2 and GAPDH protein expression in time scale 0-24hr in wildtype MDA MB231. E. Western blot of EEF2 and GAPDH protein in time scale 0-24hr in 3′UTRMYC1-18. F. Quantification of EEF2 protein expression normalized against GAPDH in time scale 0-24hr in wildtype MDA MB231. G. Quantification of EEF2 protein expression normalized against GAPDH in time scale 0-24hr in 3′UTRMYC1-18. H. Quantification of XRN1 mRNA expression normalized against ActB in WT, vector, and 3′UTRMYC1-18. I. Quantification of CNOT1 mRNA expression normalized against ActB in WT, vector, and 3′UTRMYC1-18.

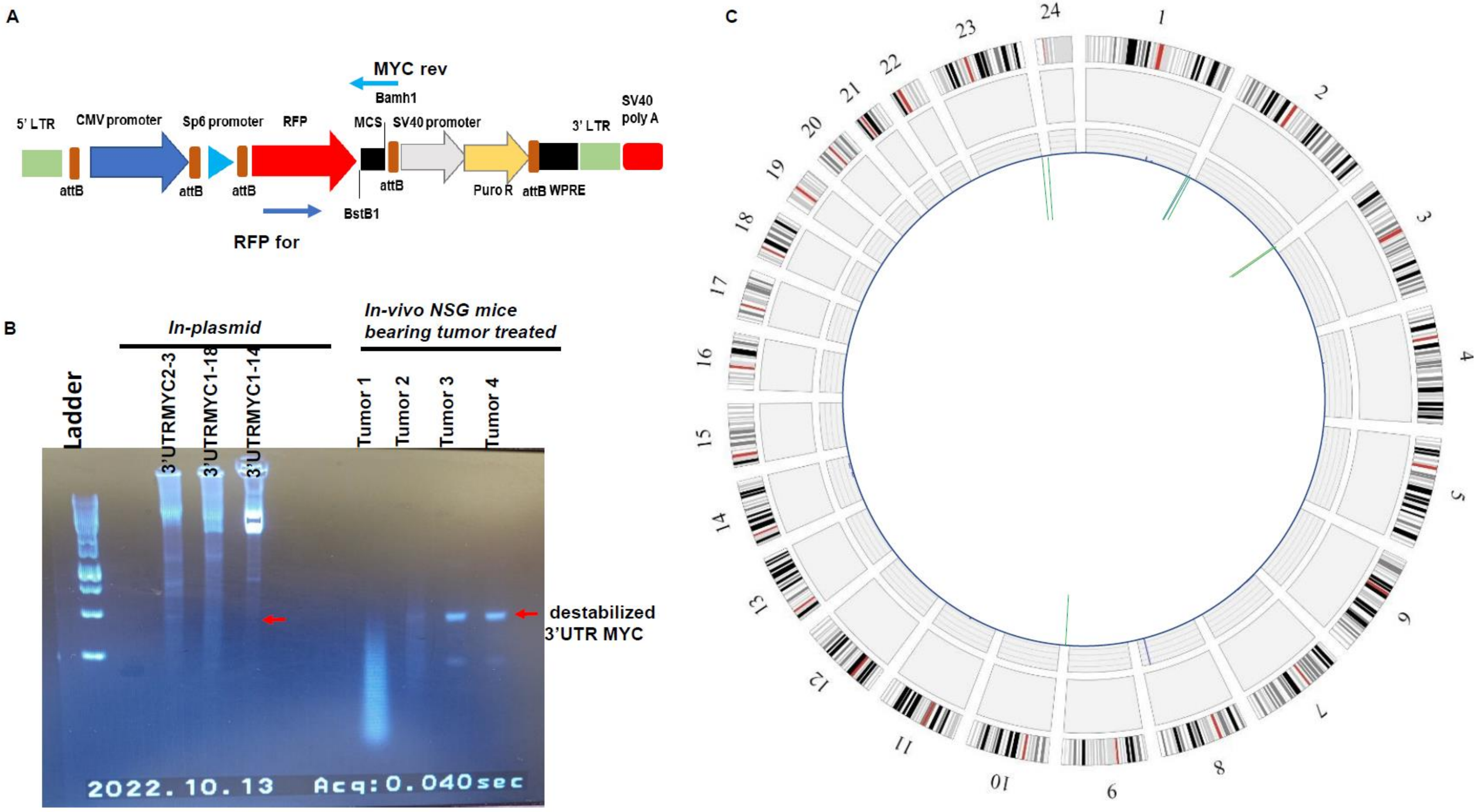

Figure S19. Detection of genome integration sites of the destabilized constructs. A. Schematic depiction of the vector constructs containing the destabilizing constructs; the arrows indicate primer positions used in the targeted sequencing. B. Gel image of the constructs amplified in the plasmid vector as well as in tumors treated with the constructs. C. Circos plot with green bars shows the genomic sites of constructs' integration.

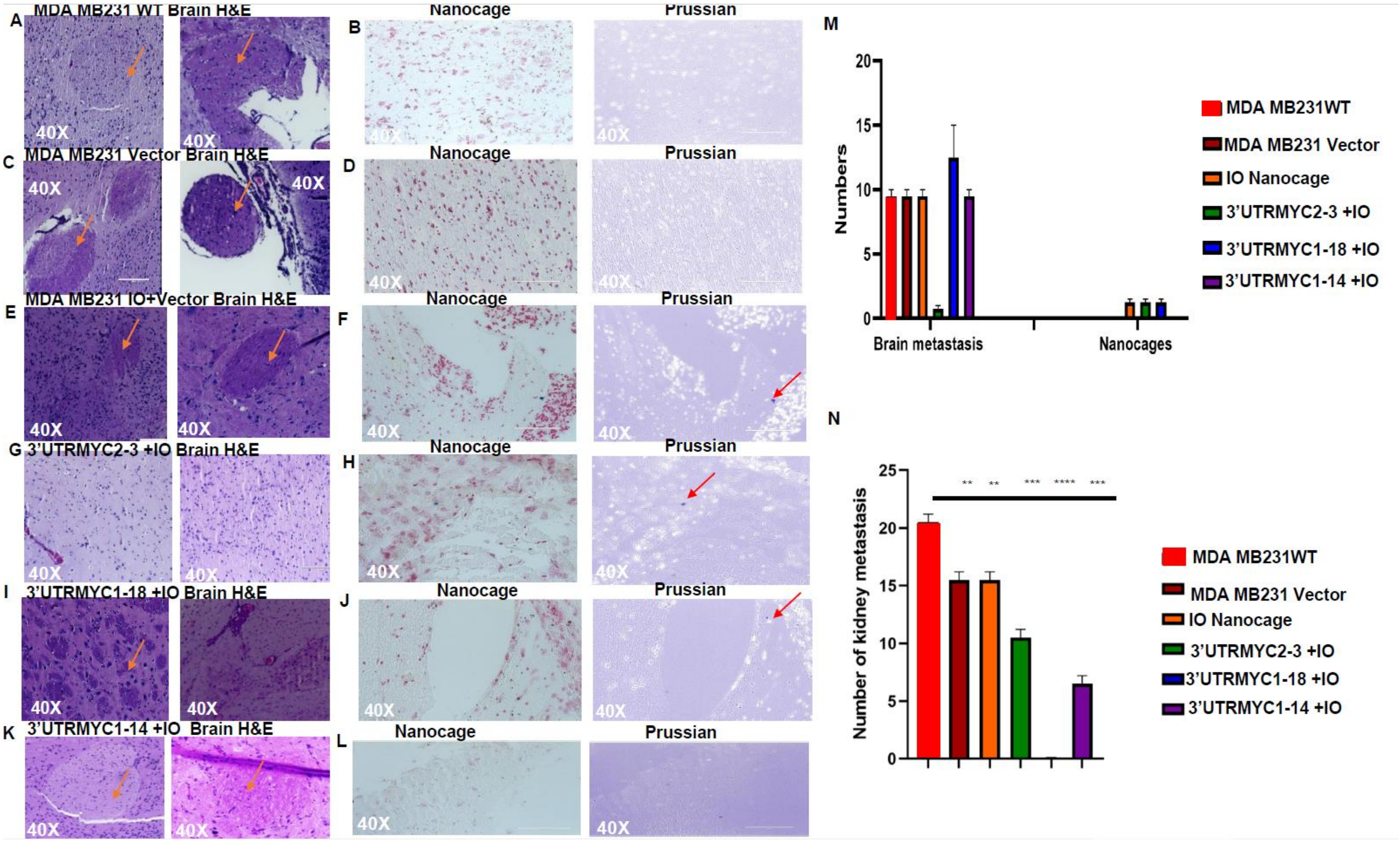

A MDA MB231 WT Brain H&E
B Nanocage
Prussian
M
C MDA MB231 Vector Brain H&E
D Nanocage
Prussian
E MDA MB231 IO+Vector Brain H&E
F Nanocage
Prussian
G 3'UTRMYC2-3 +IO Brain H&E
H Nanocage
Prussian
I 3'UTRMYC1-18 +IO Brain H&E
J Nanocage
Prussian
K 3'UTRMYC1-14 +IO Brain H&E
L Nanocage
Prussian
40X
Numbers
Brain metastasis
Nanocages
MDA MB231WT
MDA MB231 Vector
IO Nanocage
3'UTRMYC2-3 +IO
3'UTRMYC1-18 +IO
3'UTRMYC1-14 +IO
N
Number of kidney metastasis

Figure S20. H&E and Prussian blue stain of the brain tissues. A. H&E stain of the MDA MB231 no-intervention brain tissue, metastasis pointed out by yellow arrow (N = 5). B. Prussian blue stain of MDA MB231 no-intervention brain tissue. No nanocages found (N = 5). C. H&E stain of the MDA MB231 vector-treated brain tissue, metastasis pointed out by yellow arrow (N = 5). D. Prussian blue stain of the MDA MB231 vector-treated brain tissue. No nanocages found (N = 5). E. H&E stain of the MDA MB231 IO-nanocages and vector-treated brain tissue, metastasis pointed out by yellow arrow (N = 5). F. Prussian blue stain of the MDA MB231 IO-nanocages and vector-treated brain tissue. No nanocages found (N = 5). G. H&E stain of the MDA MB231 IO-nanocages 3′UTRMYC2-3-treated brain tissue, no metastasis found (N = 5). H. Prussian blue stain of the MDA MB231 IO-nanocages 3′UTRMYC2-3-treated brain tissue. Nanocages found (N = 5). I. H&E stain of the MDA MB231 IO-nanocages 3′UTRMYC1-18-treated brain tissue; metastasis found marked in yellow arrow (N = 5). J. Prussian blue stain of the MDA MB231 IO-nanocages 3′UTRMYC1-18-treated brain tissue. Nanocages found (N = 5). K. H&E stain of the MDA MB231 IO-nanocages 3′UTRMYC1-14-treated brain tissue; metastasis found marked by yellow arrow (N = 5). L. Prussian blue stain of the MDA MB231 IO-nanocages 3′UTRMYC1-18-treated brain tissue. Nanocages found (N = 5). M. Bar chart shows the quantification of brain metastasis and nanocages in the brain tissues of the control and treated animals (N = 5 mouse brains per group). N. Bar chart shows quantification of kidney metastasis in the treated and control groups (** $p < 0.01$, *** $p < 0.001$, **** $p < 0.0001$, two-tailed t-test).
