# Supplementary material for: The Engineered Drug 3′UTRMYC1-18 Degrades the c-MYC-STAT5A/B-PD-L1 Complex In Vivo to Inhibit Metastatic Triple-Negative Breast Cancer
Source: Cancers (Basel). 2024 Jul 26;16(15):2663. doi: 10.3390/cancers16152663 (PMC11311709; doi:10.3390/cancers16152663)
Supplement: Supplementary file 1 [file cancers-16-02663-s001.zip › Table S1.pdf]

Table S1: List of reagents, bacteria strains, cell lines, oligonucleotides and softwares used in the study.

REAGENT or RESOURCE	SOURCE	IDENTIFIER
**Antibodies**		
anti-c-MYC	Proteintech	67447-1-lg
anti-STAT5A/5B	Abcam	Ab20034
anti-Caspase 7	Thermofisher Scientific	9492
DAPI (Nuceloblue -nuclear stain)	Thermofisher Scientific	D3571
anti-GAPDH	Abcam	Ab8245
anti-EEF2	Abcam	Ab75748
anti-mouse and Rabbit IR 800CW dye (Li-COR),	LICOR	92632213, 92632212
Anti-PDL1	Roche	Ventana SP142
Alexa 488 (green)	Thermofisher Scientific	A30052
Alexa 610 (red)	Thermofisher Scientific	A32729
**Bacterial Strain**		
Transformation Recombination deficient E. coli	New England Biolab (NEB)	C3019H
pLenti-CMVSP6-nEGFP-SV40-PURO	Addgene	138364
**Chemicals, peptides, and recombinant proteins**		
SYBR™ Select Master Mix	Applied Biosystem	4472908
4SU Thiouridine	Sigma Aldrich	13957-31-8
3,4-dihydroxyhydrocinnamic acid (DHCA)	Sigma Aldrich	1078-61-1
Manganese (II) acetate, oleylamine, oleic acid, and iron(II) perchlorate)	Sigma Aldrich	638-38-0, 112-90-3, 749079, 335159-18-7

Table S1: List of reagents, bacteria strains, cell lines, oligonucleotides and softwares used in the study.

p-Xylene, 1-ethyl-3- [3-(dimethylamino)propyl] carbodiimide (EDC), and N-hydroxysuccinimide (NHS)	Sigma Aldrich	296333, 6066-82-6
Dimethylsulfoxide (DMSO), ferric chloride hexahydrate, and phosphate buffer saline (PBS)	Sigma Aldrich	67-68-5, 10025-77-1, 806552
Cy7.5 dye	Lumiprobe	16080
**Critical commercial assays**		
RNeasy Plus Mini Kit	Qiagen	74034
NEB HIFI DNA assembly	NEB	E2621S
Qiagen Midikit	Qiagen	12943
Qiagen gel extraction kit	Qiagen	28704
Human phospho- kinase array kit (Cat no: ARY003C	R&D Systems	ARY003C
Sanger sequencing	Psomagen	
Qiagen reverse transcription kit	Qiagen	205311
MPER Buffer	Thermofisher Scientific	78501
NEBNext® Ultra™ II Q5® Master Mix	NEB	M0544S
NEB® 5-alpha Competent E. coli (High Efficiency)	NEB	C2987H
Engineered destabilized c-MYC 3'UTR gblock	This Study<br>Synthesized by IDT	Awah CU et al, 2024
**Deposited data**		
Sanger Sequencing of c-MYC 3'UTR identifying mRNA poly U stabilizing sequences.	This Study	**10.5281/zenodo.10030728**
RNA Seq of MDAMMB231 WT, Vector, 3'UTRMYC1-18	This Study	**10.5281/zenodo.10030728**

Table S1: List of reagents, bacteria strains, cell lines, oligonucleotides and softwares used in the study.

**Experimental models: Cell lines**		
MDAMB231	ATCC	RRID:CVCL_0062
BT474	ATCC	RRID:CVCL_0179
MCF7	ATCC	RRID:CVCL_0031
T47D	ATCC	RRID:CVCL_0553
C4-2B	ATCC	RRID:CVCL_4784
RWPE1	ATCC	RRID:CVCL_3791
**Experimental models: Organisms/strains**		
Female NSG mice	Jackson Laboratory	RRID:BCBC_4142
**Oligonucleotides**		
c-MYC forward (**Gibson Assembly primer**)<br><br>gcaacgcaatGGACCCGCCCGAGCTTCGAAAAAAAAAGCCATCG	This Study<br><br>Synthesized by IDT Inc	Awah CU et al,2024
c-MYC reverse (**Gibson Assembly Primer**)<br><br>actcacattaCGGCGGGGCCGGCCCCTA	This Study<br><br>Synthesized by IDT Inc	Awah CU et al, 2024
pLenti CMVSp6 Vector forward (**Gibson Assembly Primer**)<br><br>ggccccgccgTAATGTGAGTTAGCTCACTCATTAGG	This Study<br><br>Synthesized by IDT Inc	Awah CU et al, 2024
pLentiCMVSp6 Vector reverse (**Gibson Assembly primer**)<br><br>gggcgggtccATTGCGTTGCGCTCA CTG	This Study<br><br>Synthesized by IDT Inc	Awah CU et al, 2024
DCP1A promoter forward (**Gibson Assembly primer**)<br><br>CCCTCAACTTCCGCCTCTAC	This Study<br><br>Synthesized by IDT Inc	Awah CU et al, 2024

Table S1: List of reagents, bacteria strains, cell lines, oligonucleotides and softwares used in the study.

DCP1A promoter reverse **(Gibson Assembly primer)**<br><br>CAGCCTGCAAGCTCCACTAC	This Study<br><br>Synthesized by IDT Inc	Awah CU et al, 2024
c-MYC3'UTR Forward primers (**RTPCR**)<br><br>CCTCACAACCTTGGCTGAGT	This Study<br><br>Synthesized by IDT Inc	Awah CU et al, 2024
c-MYC3'UTR Reverse primer (**RTPCR**)<br><br>GGATTGAAATTCTGTGTAACTGC	This Study<br><br>Synthesized by IDT Inc	Awah CU et al, 2024
c-MYC forward primers (**qPCR**)<br><br>GTCACACCCTTCTCCCTTCG	This Study<br><br>Synthesized by IDT Inc	Awah CU et al,2024
c-MYC reverse primers (**qPCR**)<br><br>CAGGTACAAGCTGGAGGTGG	This Study<br><br>Synthesized by IDT Inc	Awah CU et al, 2024
EEF2 Forward primer (**qPCR**)<br><br>ACGTTCACGTTCTCCACGAT	This Study<br><br>Synthesized by IDT	Awah CU et al, 2024
EEF2 Reverse primer (**qPCR**)<br><br>TGTCAGGTAAGCAGTGGGTC	This Study<br><br>Synthesized by IDT	Awah CU et al, 2024
CNOT1 Forward primer (**qPCR**)<br><br>TCTAGTGCAGCAATCCCGAA	This Study<br><br>Synthesized by IDT	Awah CU et al,2024
CNOT1 Reverse primer (**qPCR**)<br><br>GGACTGGTCACTTACATGGC	This Study<br><br>Synthesized by IDT	Awah CU et al, 2024
XRN1 Forward primer (**qPCR**)<br><br>TCCATAAAGAAGAGGCAGTGC	This Study<br><br>Synthesized by IDT	Awah CU et al, 2024
XRN1 Reverse Primer (**qPCR**)<br><br>AGATGATTGGATTTTGATGGGGT	This Study<br><br>Synthesized by IDT	Awah CU et al, 2024

Table S1: List of reagents, bacteria strains, cell lines, oligonucleotides and softwares used in the study.

**Recombinant DNA**		
3'UTRMYC1-18	This Study	Awah CU et al, 2024
3'UTRMYC1-14	This Study	Awah CU et al, 2024
3'UTRMYC2-3	This Study	Awah CU et al 2024
**Software and Algorithms**		
MyImageAnalysis	Thermofisher Scientific	https://www.thermofisher.com/us/en/home/global/forms/myimageanalysis-software.html
ENCODE Data	ENCODE	https://www.encodeproject.org/
Clustalw Omega	EMBL-EBI	https://www.ebi.ac.uk/Tools/msa/clustalo/)
DNA to mRNA translator		http://biomodel.uah.es/en/lab/cybertory/analysis/trans.htm
BioJupies	Mayan Lab	https://maayanlab.cloud/biojupies/upload/reads

Table S1: List of reagents, bacteria strains, cell lines, oligonucleotides and softwares used in the study.

GraphPad Prism	Graph Pad	www.graphpad.com
Image J	NIH	https://ImageJ.nih.gov/ij/
NEB builder	NEB	https://nebuilder.neb.com/#!/
